# Molecular Machine Learning Approach to Enantioselective C–H Bond Activation Reactions: From Generative AI to Experimental Validation†

**DOI:** 10.1039/d5sc01098e

**Published:** 2025-06-10

**Authors:** Ajnabiul Hoque, Taiwei Chang, Jin-Quan Yu, Raghavan B. Sunoj

**Affiliations:** a Department of Chemistry, Indian Institute of Technology Bombay Powai Mumbai 400076 India sunoj@chem.iitb.ac.in; b Department of Chemistry, The Scripps Research Institute La Jolla California 92037 USA yu200@scripps.edu; c Centre for Machine Intelligence and Data Science, Indian Institute of Technology Bombay Powai Mumbai 400076 India

## Abstract

Molecular machine learning (ML) has gained considerable attention in recent years. Developing ML algorithms for chemical reaction prediction is a formidable task, due to the small-sized reaction data it often presents, besides the sparsity and skewed distribution. While previous ML studies offered effective predictions on known reactions, efforts in using deep generative models for guiding new reactions and their prospective validation are rare. We harness both predictive and explorative abilities of deep learning on an important catalytic asymmetric β-C(sp^3^)–H activation reaction, consisting of 220 experimentally reported examples that differs primarily in terms of the substrate, catalyst, and coupling partner. A transfer learning approach using a chemical language model, pretrained on 1 million unlabeled molecules followed by fine-tuning on this reaction data set, is adopted. Our ensemble prediction (EnP) model, where 30 fine-tuned CLMs concurrently predict the %*ee* of test set reactions, is highly reliable. Another language model, fine-tuned on the 77 known chiral ligands as used in the above reactions, is employed for generating novel ligands of high validity and novelty. A proof of concept wet-lab experimental validation reveals that most of the ML-generated reactions are in excellent agreement with the EnP predictions. Results also caution the prospects of ML-driven reaction development for ligand design and emphasize the importance of domain experts in key decisions.

## Abstract 2

The Generative-AI for novel chiral ligand design and an ensemble prediction for %*ee* can be used in developing asymmetric catalysis, as evident from our successful prospective experimental validation.

## Graphical abstract

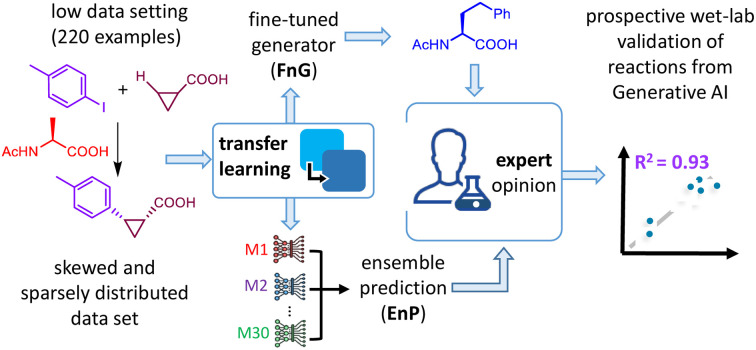

## Introduction

Empirical efforts are inevitable in chemical reaction development when one works within a relatively smaller set of controllable variables toward realizing a desired objective.^1,2^ During these empirical loops, reaction conditions are typically altered while keeping the reactants the same.^3,4^ In the developmental stages, such as in the contemporary practice of homogeneous catalysis, a good amount of data is generated, but it largely remains underutilized.^5,6^ Recent trends suggest an increasing interest in tapping the potential of data-driven reaction development.^7–9^ For instance, ML algorithms are now available that harness the available data on molecular properties and reactions.^10–12^ The deployment of ML for predicting the site selectivity in an Ir-catalyzed borylation reaction is particularly noteworthy as the predictions were substantiated through experimental validation.^13^ Similarly, a graph-based deep learning (DL) model with multi-objective capabilities could predict the yield and selectivity of borylation reactions.^14^ Another avenue in reaction development has been to combine robotics and automation to minimize time and material resources.^15^ The availability of a high-throughput experimental setup has also helped in providing good-quality reaction data sets.^16,17^ All these trends point to a growing need for robust ML models suitable for chemical reactions.

A good number of molecular ML models for chemical reactions have already become available, many of them offering impressive performances.^18,19^ These studies tend to recommend their best-trained model for different scenarios found in reaction outcome prediction tasks.^20–22^ Predictions of yield, selectivity, prospective target identification in reactions, *etc.*, have become feasible and affordable. However, relying on one fully trained ML model might limit model generalizability when predicting on unseen reactions. It is worth reckoning that the idea of weak and strong learners is effectively incorporated in ML models such as the random forest (RF). The very use of several decision trees in RF models, or even an extended version such as ensemble RF, have been in use.^23,24^ These methods provide multiple predicted values for every sample in the test set. In a conceptually different approach, such as in DL, a fully trained model is generally used for predicting on unseen samples. We envisaged using multiple independent DL models built on different training sets. We denote this as ensemble prediction model, EnP (*vide infra*).^25,26^ The proposed ensemble prediction method assumes additional significance in this work as it comprises of a generative ML task.

**Fig. 1:**
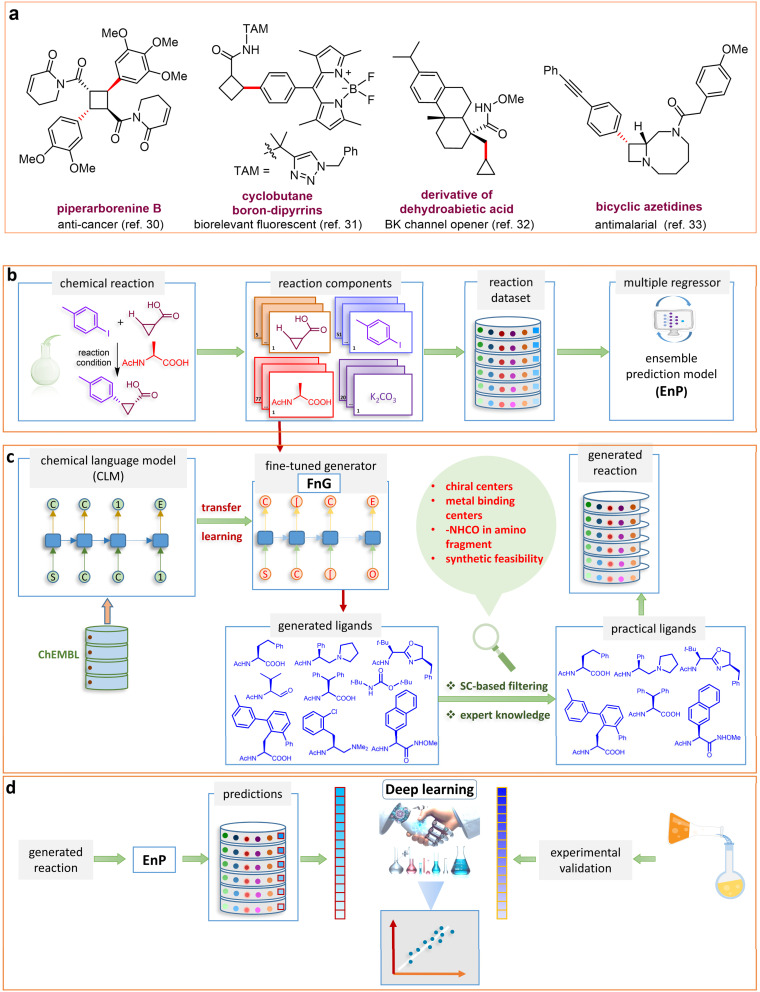
(a) Biologically important compounds whose synthesis involves C(sp^3^)–H bond activation reactions. Key elements of our ensemble prediction (EnP) model workflow and prospective wet-lab validations. (b) An example of an asymmetric β-C(sp^3^)–H reaction [*chemical reaction*], with reactant variations through their respective quantities denoted in the left corner [*reaction component*], forming the reaction data set [*reaction data set*]. This data set is used in developing multiple regression models, leading to the EnP model, (c) training of an LSTM-based chemical language model (CLM) on the ChEMBL data set, followed by fine-tuning on the target task containing 77 chiral ligands to develop FnG [*fine-tuned generator*]. The fine-tuned generator is employed to generate novel chiral ligands [*generated ligands*], which are then filtered based on criteria such as the presence of a chiral center and the –NH(C=O) fragment, to make a practically meaningful set of chiral ligands [*practical ligands*]. These ligands are then concatenated with relevant reaction components to form a complete reaction set [*generated reaction*]. (d) The EnP model predicts %*ee* of the generated reactions, which are subsequently compared with the values obtained from our new wet-lab experiments.

It is timely that the capabilities of ML are put to immediate use for emerging classes of reactions. For example, the unprecedented popularity of catalytic C–H bond activation reactions makes them an ideal research problem for examining the efficacy of ML.^27–29^ A broad array of applications of C–H bond activation reactions in obtaining high-value target compounds, such as drugs and biologically active compounds, are known (Fig. 1, panel-a).^30–33^ It should be acknowledged that the real experimental data accrued over decades of work in this domain are sparse and imbalanced.^34,35^ Such data sets are likely to have more samples (reactions) in the low or high enantiomeric excess/yield regime, giving rise to class imbalance.^36^ Similarly, a lot of instances may form clusters around some of the most frequently used reactants or catalysts, rendering an overall sparse distribution in the data set.^37^ Besides the complex and non-linear relationship between the labels (*i.e.*, %*ee*) and feature space representing the samples, these distribution characteristics are likely to make the development of ML models a challenging task. While DL models generally require a large pool of data, generating them could be resource-intensive and time-consuming. In this context, the use of transfer learning (TL) could become an effective approach that transfers knowledge from related tasks to a data-scarce target task, applicable both in generative and predictive settings.^38^

One of the vital questions at this juncture is to ask whether or not the prospects of DL-based methods could be effectively scrutinized. In other words, would DL models for reaction outcome prediction hold good, when subjected to prospective wet-lab validation? Addressing such questions within the context of a small data regime assumes high significance as it represents the real-world situations found in reaction development. Should the DL-driven reaction development become viable, the model should be able to learn from limited, sparse, and imbalanced data. These very aspects constitute the major objectives of this work.

Given our continued research efforts in C–H bond activation reactions^39–43^ as well as the need for a timely evaluation of DL-driven reaction development, we became interested in (i) developing robust DL models for enantioselectivity predictions in a catalytic asymmetric β-C(sp^3^)–H bond activation reaction, (ii) demonstrating the capabilities of generative-AI in discovering novel reactions within this class, (iii) subjecting them to prospective experimental validation, and (iv) analyzing the prospects of our approach in a self-critical manner to evaluate the role of domain experts in such endeavors.

## Results and discussion

We have organized the results and discussion into four sections, which begin with data set details and the introduction of our EnP model, followed by the development of a transfer learning-based fine-tuned generator (FnG) for chiral (amino acid) ligands. Generative DL for novel chiral amino acid ligands and their wet-lab experimental validation are subsequently presented. Finally, we emphasize the importance of the active participation of domain experts and caution about the liberal forward pass of generated candidates for experimental validation.

### Data set and DL model

Here, we provide an overview of our data set and the DL model-building protocol. First, we describe the important characteristics of our manually curated data set from the most recent literature on catalytic asymmetric β-C(sp^3^)–H bond activation comprising a total of 220 reactions.^44–47^ A given sample (*i.e.*, a reaction) is a concatenated representation of its participating entities, such as the catalyst precursor, chiral ligand, substrate, coupling partner, solvent, base, and reaction condition. Fig. 1 (panel-b) provides an overview of these reaction constituents. A representative reaction between cyclopropyl carboxylic acid and *p*-iodotoulene is shown in the inset. The full data set consists of 5 cyclopropyl motifs undergoing β-C(sp^3^)–H functionalization with any of the 51 aryl halides in the presence of one among the 77 chiral amino acid ligands and involves one of the 20 bases. The fact that the participating molecules are diverse and only a few combinations between them were reported to date makes the data set inherently sparse. Affording decent accuracies in DL predictions is, therefore, expected to be a challenge. More importantly, the DL model should remain sufficiently generalizable when faced with unseen samples so as to render it a practically useful tool for guiding new experiments.

In view of the above-mentioned expectations on the DL model and the nature of the data set, we have employed the ULMFiT-based chemical language model (CLM) in this work. We trained an RNN-based ULMFiT language model^48^ to learn the molecular representations from the SMILES (simplified molecular input line entry system) input of reactions given in the form of concatenated SMILES of individual reactants.^49^ During training, the model learns to predict the probability distribution of the next character from a given sequence of strings, similar to that in natural language processing. SMILES strings encode atomic connectivity as well as atom and bond types, thus offering a comprehensive representation of all participating molecules in the reaction. Since the DL models, such as the ULMFiT, require large data for effective learning, first, we pretrained the model using a large library of unlabeled molecules drawn from the ChEMBL database (Fig. 1b).

It is important to note that the pretraining of the language model in this work is utilized for two major downstream tasks. As in a TL setting, the pretrained weights and biases are first used for fine-tuning the target task reaction data set to predict the %*ee* as the output of our regression model, which is termed as EnP model.^49–51^ In a separate task, we fine-tuned a target task data set consisting of 77 chiral (amino acid) ligands as used in the asymmetric β-C(sp^3^)–H bond activation reaction earlier. The idea here is to exploit the model for subsequent generative tasks (*vide infra*) (Fig. 1c). This fine-tuned generator is denoted as FnG.^52^ Here, we endeavor to generate novel chiral ligands suitable for this class of reaction, predict their efficacy using the EnP, and subject them to prospective wet-lab experimental validation.

### TL-based ensemble prediction (EnP) regressor

**Fig. 2:**
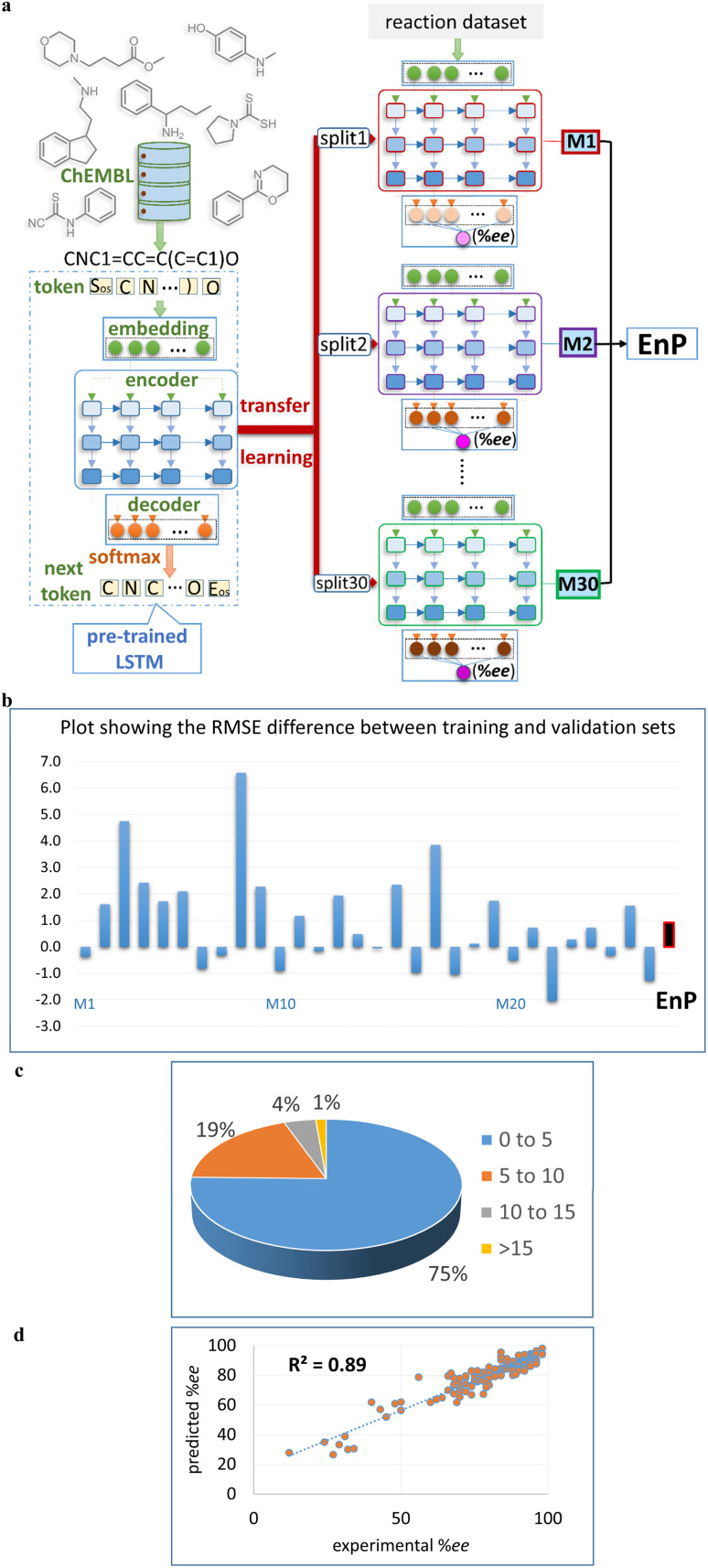
(a) An overview of the TL model employed in fine-tuning a regression task. Here, molecules are encoded as SMILES strings, which are then tokenized into individual elements representing atoms (shown in light-yellow colored boxes). These tokens serve as input for the CLM model. The fine-tuning involves transferring pre-learned weights to a series of regression models (M1 to M30, as shown using red colored arrows). (b) The bar plot showing the difference between the train and validation RMSE (root mean squared error) across all the regressors and the EnP model. Visualization of the absolute difference between experimental and the EnP predicted %*ee* for the test samples across all 30 runs using (c) pie chart as well as (d) the corresponding parity plot.

As stated in the objectives of this work, we want to examine the capabilities of generative-AI for new chiral ligand identification and their prospective wet-lab experimental verification. To achieve this goal, a good quality regressor for predicting the %*ee* of the reactions due to the novel chiral ligands generated by our fine tuned generator (FnG) should be used. To this end, we designed a transfer learning based ensemble prediction regressor, denoted as EnP, to provide robust predictions on these unseen reactions. Since the training data is sparse and imbalanced, the extent of over/under-fitting could become an issue if only one fully trained DL model is employed for predicting on such reactions involving the new chiral ligands.^25,26^ Hence, multiple fine-tuned models were separately trained on randomly selected training sets to make predictions on the test samples, which are unseen reactions as far as the model is concerned. In this approach, 70% of random samples form a training set for model M1, and thirty such independently trained models give M1 to M30 (Fig. 2a).^53,54^ The key motivation for adopting such an approach can be appreciated by comparing the train and validation performances across these regression models, M1 to M30, as shown in Fig. 2b. It can be gathered that several models (M3, M9, *etc.*) exhibit notable differences in RMSE between the training and validation set, suggesting over-fitting if we were to use just one model to predict the unseen samples. On the other hand, the difference between the training and validation RMSEs obtained for the EnP protocol is very small (shown at the far right end). A robust regression model is crucial to this study, as our objective includes predicting %*ee* of the generated reactions, which are naturally out-of-bag samples.^55^ It is desired that the predictions should help in guiding subsequent wet-lab validation, where an informed choice of substrates suitable for the generated catalysts can be made prior to experimental validation.^56^

The quality of predictions can also be gleaned both from theparity plot and the pie chart provided in Fig. 2c and d. It can be noted that about 94% of predictions across all 30 runs remain within 10 units of the previously reported experimental %*ee* values. Similarly, the parity plot conveys a very good correlation between the %*ee* predicted by the EnP model and the corresponding experimental values a coefficient of determination (*R*^2^) of 0.89. More importantly, our EnP model could perform better than the other regression models such as RF, deep neural networks (DNN), and AttentiveFP.^57^ All these are good indicators of the effectiveness and reliability of our model in the %*ee* prediction task.^58^ A more complex transformer-based architecture, such as the T5Chem, could offer a test RMSE of 9.95±1.81, which is inferior to our EnP regressor with an RMSE of 7.57±1.31.^59^ This is particularly interesting, given that T5Chem is pre-trained on ∼97 million molecules and fine-tuned for %*ee* prediction tasks. Comprehensive details of model architecture, hyperparameter selection, and validation procedures for all these baseline models are well documented in Section 10 of the ESI.†

**Fig. 3:**
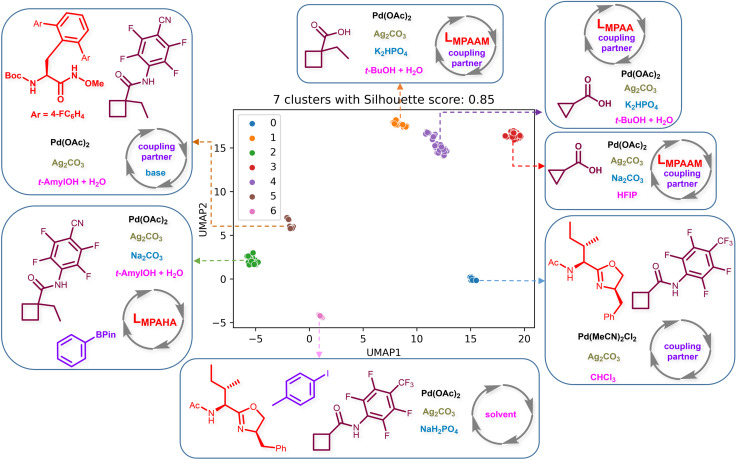
The UMAP plot of the encoder output of a representative regression model (M2 which has a similar performance as that of the EnP model) and *k*-means clustering on the first two principal components that shows that the model learns chemically meaningful details. The chiral ligands in the inset are denoted using L_MPAA_ (mono-N-protected amino acid), L_MPAHA_ (mono-N-protected α-amino-*O*-alkyl hydroxamic acid), L_MPAAM_ (mono-N-protected amino-alkyl amine), and L_APAO_ (N-acyl-protected amino oxazoline).

After having developed a TL-based EnP model, we became interested in probing the key characteristics of what the model could learn from the input data. To facilitate visualization of the complex and high dimensional encoding vector, we have used the UMAP (uniform manifold approximation and projection) plots that project it onto a reduced space (see Section 5 in ESI† for more details of the UMAP plots).^60,61^ Seven distinct clusters (labeledas 0 to 6) with high Silhouette scores are discernible in Fig. 3. Interestingly, most of these clusters could be generally characterized as belonging to different substrates and chiral ligands. For instance, clusters 0, 1, 2, and 4, respectively, represent reactions involving chiral ligands L_APAO_, L_MPAAM_, L_MPAHA_, and L_MPAA_. Clusters 1 and 3, although they share the same ligand (L_MPAAM_), the substrates involved are found to be different (cyclopropanes and cyclobutanes). Similarly, clusters 0 and 6 contain different coupling partners/solvents, while the chiral ligand belongs to the L_APAO_ family. Identification of these chemically meaningful clusters from the latent space of the encoding vector engenders considerable confidence in our model as being able to learn from the given representation of chemical reaction. We plan to utilize this knowledge acquired by the DL model in our generative tasks wherein we sample new chiral ligands from disparate regions of the latent space (*vide infra*). This would ensure sufficient diversity among the generated reactions employing such chiral ligands.

### Latent space generation using TL

Next, we set out to develop a TL-based fine-tuned generator (FnG) wherein we aim to explore the latent space of the DL model by identifying novel catalysts for the asymmetric β-C(sp^3^)–H bond activation reaction. As discussed in the previous paragraphs, our DL model is pretrained using a large number of unlabeled molecules from the ChEMBL database and fine-tuned on the catalytic reaction of interest. The model does well,both in regression as well as in learning reaction-specific details. Motivated by these, we have now fine-tuned another target task, denoted as FnG, comprising 77 known chiral mono-protected amino acid ligands, such that the sampling of the latent space of such a DL model could be utilized for generating new chiral ligands.

We were pleased to note that the FnG could generate chiral (amino acid) ligands with a high validity of 99% besides excellent uniqueness (98%) and novelty (98%). While these ligands are all valid molecules, our main goal is to expand the ligand library in such a way that they become useful, to the extent possible, when deployed in real-world wet lab validation. We have therefore considered certain chemically relevant criteria to choose from the pool of 490 generated molecules. These filters mandate the presence of (i) at least one, but not more than two, chiral center(s), (ii) N and O-donor sites for its binding to transition metals to enhance their likelihood of being a catalyst in our reaction, and (iii) the NH(C=O) moiety near the N donor to facilitate β-C(sp^3^)–H bond activation.^62,63^ With these mechanistically informed filters in place, we could identify 73 chiral amino acid ligands from among the 490 candidates generated by the model. We consider it important to sample novel chiral ligands from the neighborhood of the experimentally known ligands. Such ligands are more likely to follow a similar mechanism to those of the known reactions, thereby acting as an implicit safeguard toward rendering the predictions more realistic.

**Fig. 4:**
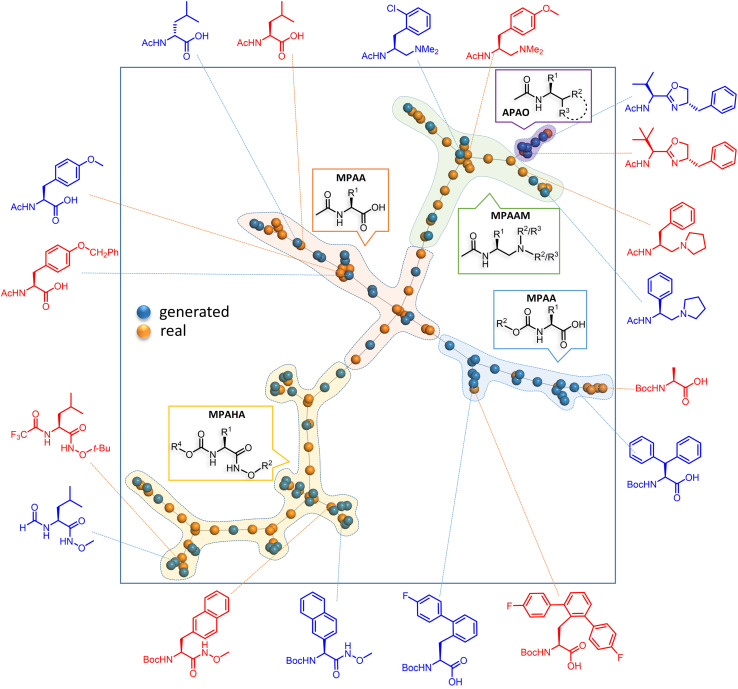
Representative examples of the generated chiral ligands, along with their closest experimentally known analogues, are shown along the periphery of the square box. The training and generated sets are respectively shown in orange blue dots to convey their structural similarities. The fingerprints in TMAP (Tree MAP) undergo min hashing with a weighted scheme to ensure compatibility with the LSH (locality-sensitive hashing) forest (see Section 8 in the ESI† for more details).

We were further pleased to learn that our FnG model offered better performance than the other SOTA models deployed in molecular generation, including genetic algorithm,^64^ graph-based generative models,^65^ and virtual screening (VS) for the generation/filtering of chiral ligands. The TL-based ULMFiT method emerged as the top-performing model, as indicated by the percentage of novel and practically useful molecules generated, besides their Fréchet ChemNet Distance (FCD).^66^ A detailed comparison between these generative models is provided in Section 11 of the ESI.† Specifically, our FnG model could achieve a significantly lower FCD score of 4.1 compared to VS, whose FCD is as high as 32.3. This highlights the superior ability of our model in generating chemically similar ligands to those in the training set, implicitly offering higher similarity in their catalytic mechanism. The success of this approach also indicates the potential of a complementary role generative models can play in catalyst discovery. By integrating domain expertise into the training data selection (in the present case, a smaller sizedthe target data set is manually curated) and the use of TL to guide the generation of chiral ligands (as illustrated in Fig. 4), the FnG model effectively reduces the search space for identifying promising catalysts. It is important to consider generative models as tools that augment, rather than replace, domain knowledge. This TL-based model serves as a potent platform for combining human expertise with data-driven techniques, allowing for efficient navigation of realistic chemical spaces and addressing the limitations of small data sets.

The TMAP plot, as given in Fig. 4, helps in assessing the similarities between the generated chiral amino acid ligands and those in the training set (experimentally known ligands).^67^ The spread of the orange and blue dots in the plot and the proximity between them indicate a couple of chemically interesting aspects. The generated chiral ligands span sufficiently wider regions of the chemical space while maintaining structural similarity to the training examples, both suggestive of efficient exploration of the latent space of the DL model. It is interesting to note that one of the generated APAO ligands, shown in the upper right region, is somewhat similar to the corresponding training set analogue. However, the critical substituent at the chiral center in the generated ligand is a *tert*-butyl as opposed to an isopropyl group in the training set. Similarly, one of the generated MPAHA ligands shown in the lower left side bears a –CF_3_ group in place of –H in the training set. These changes noticed in the generative task, as explored by the model, are indeed chemically reasonable.

**Fig. 5:**
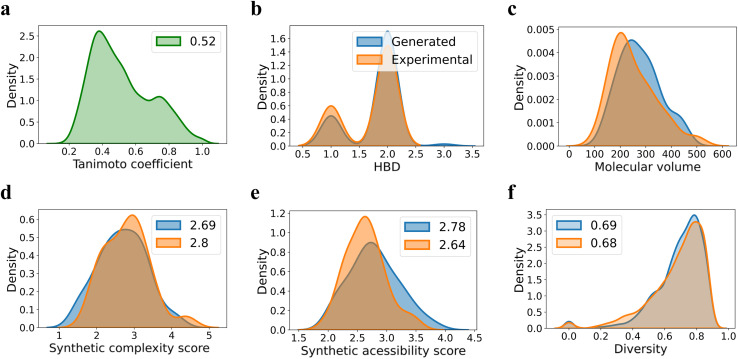
Comparison of the generated and training set ligands using (a) Tanimoto coefficient, (b) hydrogen bond donor (HBD) count, and (c) molecular volume. Other relevant metrics of importance such as (d) synthetic complexity (SC) score, (e) synthetic accessibility (SA) score, and (f) chemical diversity. The Tanimoto coefficient for each of the generated ligand is computed with respect to every ligand in the training set and the distribution of the pairwise similarity scores is plotted. The diversity is calculated in a pairwise manner within the generated ligands as well as within those in the training set.

Since quite a few new chiral ligands are generated, a comparison of each of them with those in the training set might not be desirable. Hence, a widely used metric such as the Tanimoto coefficient is employed for quantitative comparisons^68^ (see Section 8 in the ESI†). A mean similarity score of 0.52 between the generated and training set chiral amino acid ligands indicates a close structural resemblance. At the same time, the diversity of 0.42 among the generated ligands suggests their reasonable spread in the chemical space. The plots shown in Fig. 5 convey similarity, diversity, and a few other desirable molecular properties relevant to catalysis. Physicochemical properties such as the number of H-bond donors, molecular volume, and steric characteristics around the donor sites can influence the nature of binding of chiral ligands to the transition metal.^69^ It can be discerned from Fig. 5 that most of these properties of the generated set remain similar to those in the training set. We consider these aspects an advantage of our FnG in keeping the novel design space within chemically manageable regions.

A set of additional but more valuable metrics for evaluation of the likely utility of the generated chiral ligands is to consider synthetic accessibility score (SAS) and synthetic complexity score (SCS).^70,71^ The SAS, ranging from 1 (easy) to 10 (difficult), considers molecular size, substructures, and complexity, while SCS (varies from 1 to 5) measures structural complexity, including functional groups, ring systems, and stereocenters. Given that a lower SA score implies the ease of synthesis, one could employ these to choose the right candidate for prospective experimental validation (*vide infra*). It can be gathered from panels d and e in Fig. 5 that the mean SA(2.78) and SC(2.69) of the generated chiral ligands are comparable tothat of the corresponding values of the training set, which are respectively 2.64 and 2.80. While these values provide an initial confidence in the synthetic feasibility of the generated ligands, the use case scenarios might become tricky as exemplified in the later section where we discuss our experimental validation efforts.

With the ML-generated chiral amino acid ligands with us, we became interested in evaluating their efficacies in the asymmetric β-C(sp^3^)–H bond activation reaction. We use our EnP regression model to afford good quality %*ee* predictions for every new reaction due to the use of these novel ligands. It is known from our training set with 220 experimentally known reactions that the %*ee* depends predominantly on the nature of the chiral ligand used in conjunction with a transition metal. Each reaction in the training set involves a catalyst consisting of a chiral ligand bound to a transition metal (Fig. 1b). There are 77 unique chiral ligands in the training set giving rise to a total of 220 known reactions. Any one of these chiral ligands could be replaced with the generated ligand while keeping the other reaction components, such as the cycloalkane and coupling partner, the same. Through such replacements, we get 9855 possible reactions (73 new chiral ligands multiplied by 135 known combinations of reaction partners other than the chiral ligands) and their predicted %*ee*, facilitating quick identification of promising ligands (or even the choice of substrates/coupling partner) from among the generated set (see Section 9 in ESI† for more details).

**Fig. 6:**
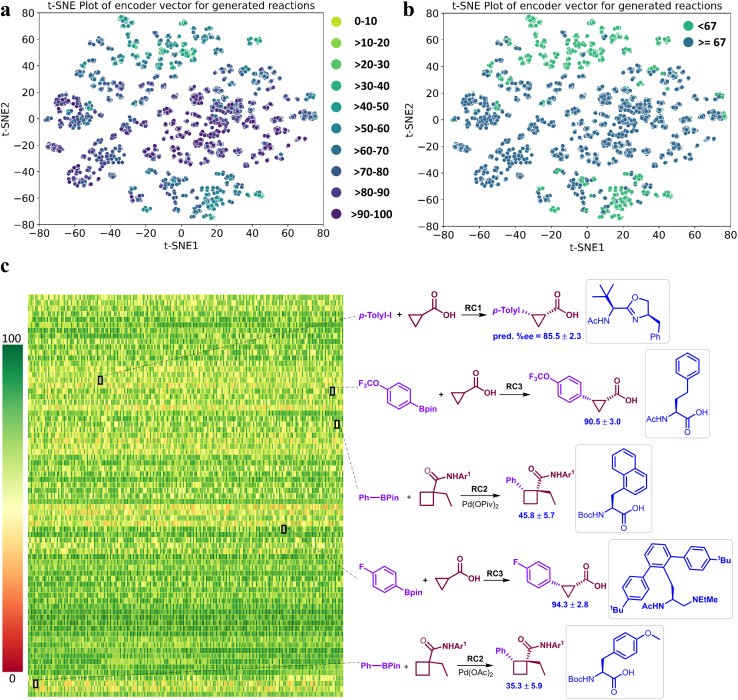
The t-SNE plots obtained using the latent vectors of a representative regression model, which bears a comparable performance as that of the EnP model, for asymmetric β-C(sp^3^)–H bond activation reactions due to the generated chiral amino acid ligands. (a) Lighter to darker color gradient represents low to high %*ee* as predicted by model M2. (b) A binary class representation using the mean of the predicted %*ee* as the class boundary to elicit quicker identification of good or poorly performing reactions, (c) A heatmap of the predicted %*ee* by the EnP model of 9855 reactions corresponding to the 73 newly generated chiral ligands, where each cell/pixel corresponds to a particular combination of substrate, coupling partner, generated chiral ligand (in the inset), additive, and other reacting components. A select set of new reactions and their predicted %*ee* are shown on the right side. Reaction conditions (RC): RC1 = [Pd(MeCN)_2_Cl_2_ (10 mol%), ligand (10 mol%), Ag_2_CO_3_ (2.0 equiv.), Li_3_PO_4_ (2.0 equiv.), CHCl_3_ (1.0 mL), 80 ^0^C, 24h]. RC2 = [Pd(OAc)_2_ (10 mol%), ligand (11 mol%), Ag_2_CO_3_ (1.5 equiv.), Na_2_CO_3_ (2.0 equiv.), BQ (0.5 equiv.), H_2_O (5.0 equiv.), *t*-AmylOH (0.5 mL), 70 ^0^C, 24h]. RC3 = [Pd(OAc)_2_ (10 mol%), ligand (20 mol%), Ag_2_CO_3_ (1.5 equiv.), K_2_HPO_4_ (1.5 equiv.), *t*-BuOH (1.0 mL), H_2_O (10.0 equiv.), BQ (0.5 equiv.), 80 ^0^C, 12h].

It is important to reckon that the DL model has learned chemically significant characteristics from the training set (Fig. 4) and has also been able to generate new chiral ligands for the reaction of interest. Motivated by the fact that our DL model effectively learns from the reaction encoding (Fig. 3), we desired to make ML-based recommendations for new reactions. This can be done either by directly predicting the %*ee* for any newly generated chiral ligand or by choosing them from the t-SNE projections of the latent vectors of the reactions due to such ligands. For instance, a prospective higher %*ee* reaction can be located from Fig. 6a/b or from the heat map in Fig. 6c. Alternatively, a simpler and approximate measure of the expected outcome, higher or lower than the mean %*ee* of 67, can also be gathered from the t-SNE plots in Fig. 6b.^72^ The region of reactions above the mean %*ee* can be readily identified between 20 and -40 along the t-SNE2 axis. Similarly, one can also choose several high %*ee* reactions from the central region enclosed between (20,-20) in t-SNE2 and (-40,60) in t-SNE1 of Fig. 6a.^73^

The heat map depiction of the predicted %*ee* of new reactions (Fig. 6c) can be analyzed in different ways. For example, it can help make an informed choice as to which among the new reactions would be of greater interest. Each color pixel in this plot represents the predicted %*ee* for one of the generated chiral ligands in a reaction, and each row conveys the performance of the same ligand across all 135 unique combinations of reacting partners. Some of the pixels are shown expanded to the right to explicitly display a set of representative reactions belonging to both high and low %*ee* ranges. It is important to recollect that the training data is skewed toward the higher %*ee*, leaving very few training samples in the lower %*ee* region. Hence, it is important to consider the generated reactions even from the lower end of the output for wet-lab validations. This would serve as a good demonstration of the robustness of our model across all likely outcomes.

### Prospective experimental validation

As we move on to the wet-lab validation phase of this work, we wish to highlight that our recommendation to the Yu lab contained only the details of the reactions (primarily the generated catalyst/chiral ligand and substrates), but not what the predicted %*ee* were. This approach was to minimize a potential human bias of having known the predicted %*ee*, or clues even as broadly as high or low values. Upon completion of these experiments, %*ee* values were mutually disclosed in a joint meeting wherein the last two columns of Table 1 were filled simultaneously. To begin with, we present the most successful reactions compiled into Table 1, where the cycloalkane carboxylic acid or *N*-aryl amides (substrate) undergoing the asymmetric β-C(sp^3^)–H bond activation and the corresponding coupling partner are shown respectively in columns 2 and 3. In the subsequent columns, the name of the chiral ligand and reaction conditions are provided. The last two columns enable a direct comparison of the DL-predicted %*ee* of each of these reactions with those obtained through the wet lab experiments.

**Table 1:** Comparison of the ML-Predicted %*ee* of the Generated Reactions with Those Obtained from Wet-lab Experiments

entry	substrate^a^	coupling partner	ligand	reaction condition^b^	pred.^c^ %*ee*	exp. %*ee*
1	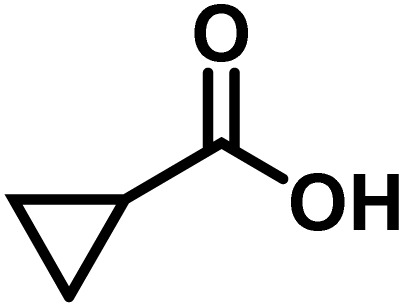	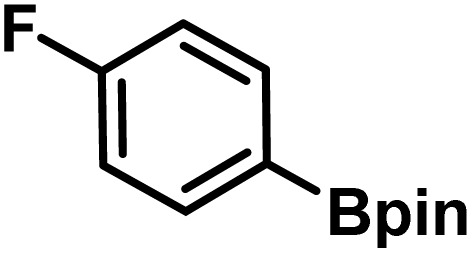	L5	RC3	94±3	94
2	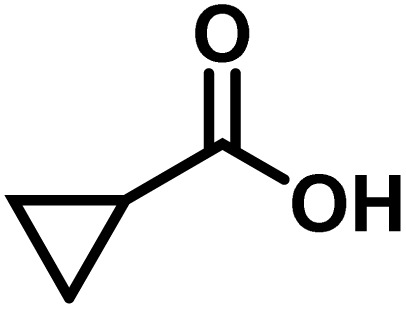	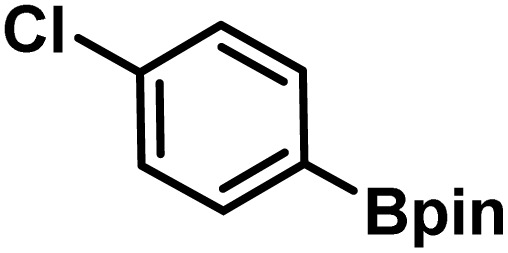	L5	RC3	94±3	94
3	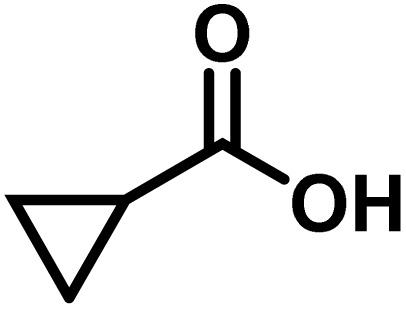	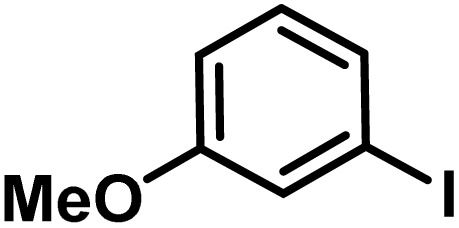	L5	RC4	93±3	86
4	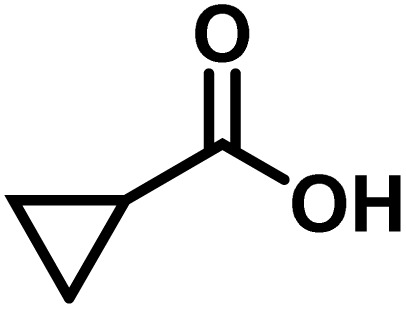	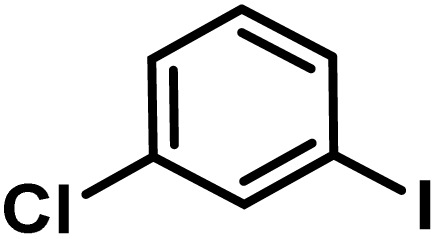	L5	RC4	93±3	85
5	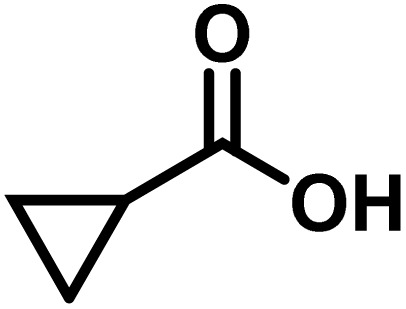	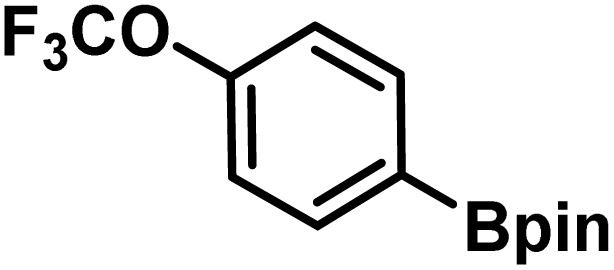	L4	RC3	90±3	86
6	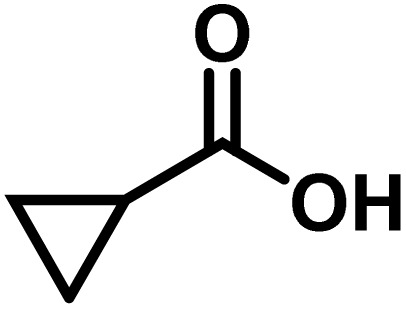	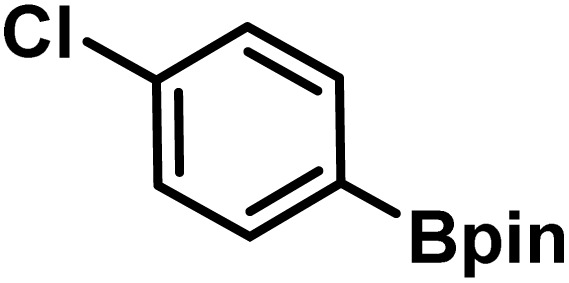	L4	RC3	90±3	85
7	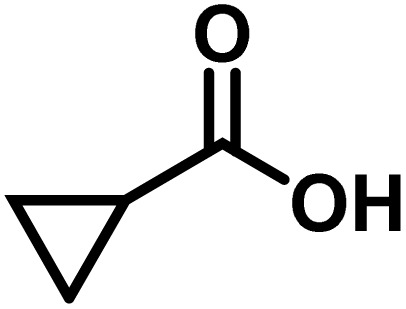	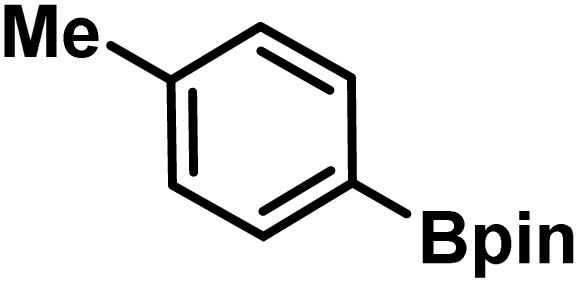	L4	RC3	90±3	86
8	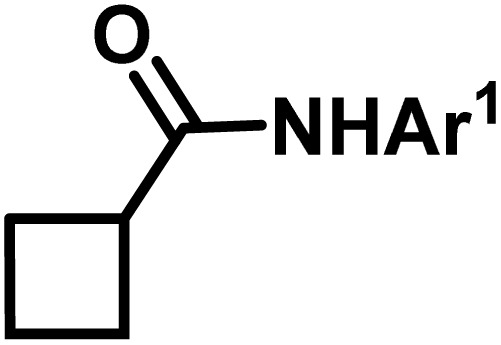	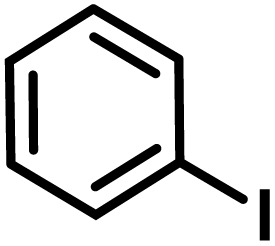	L6	RC1 (Li_3_PO_4_)	85±2	80
9	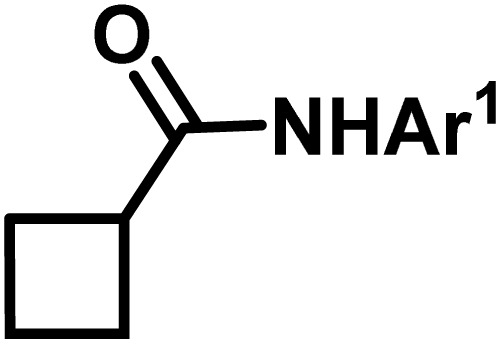	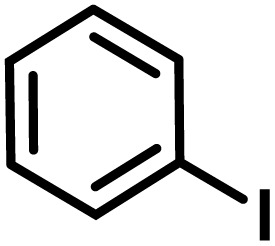	L6	RC1 (Na_3_PO_4_)	84±3	81
10	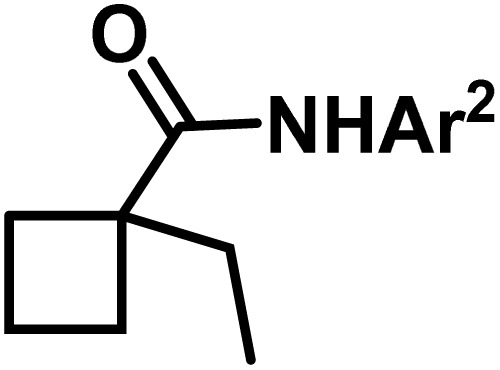	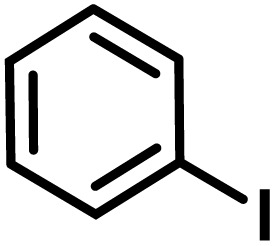	L7	RC2 (Pd(OPiv)_2_)	46±6	30
11	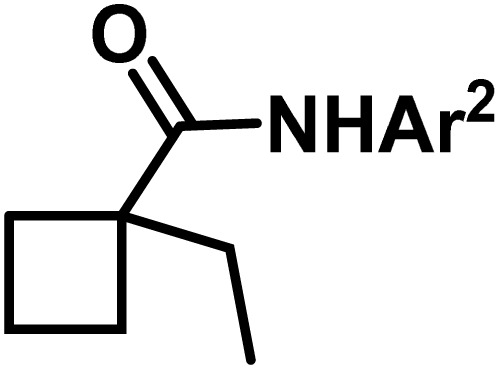	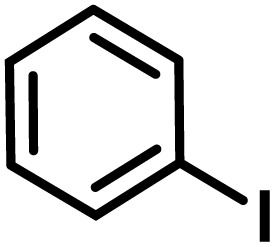	L8	RC2 (Pd(OAc)_2_)	35±6	23
12	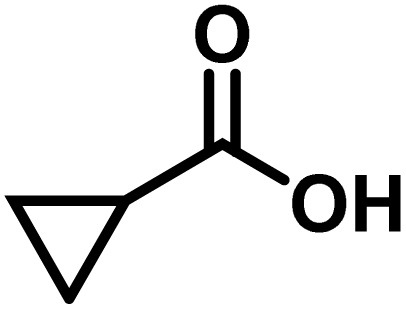	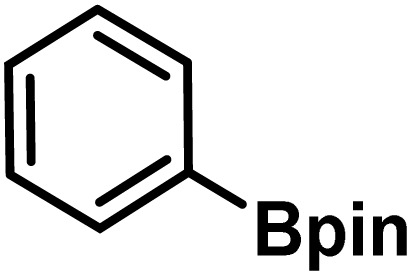	L(Ac-Phe-OH)	RC3	90±4	90
13	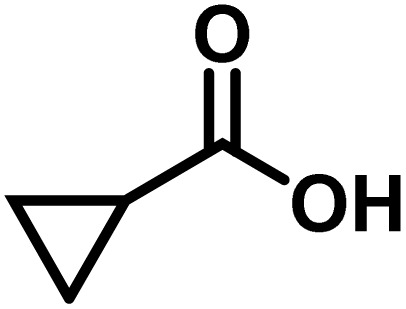	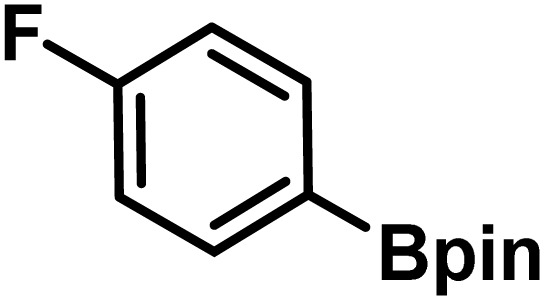	L(Ac-Phe-OH)	RC3	90±3	91
14	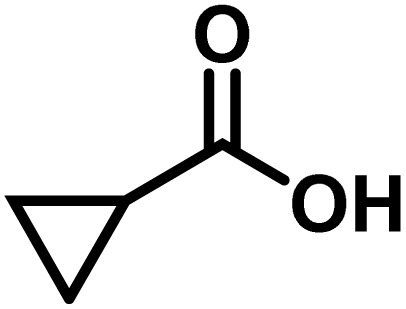	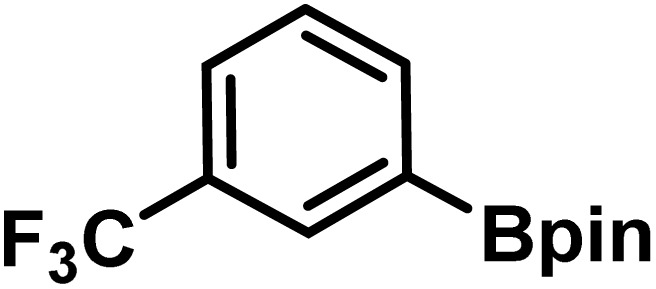	L(Ac-Phe-OH)	RC3	87±3	87
15	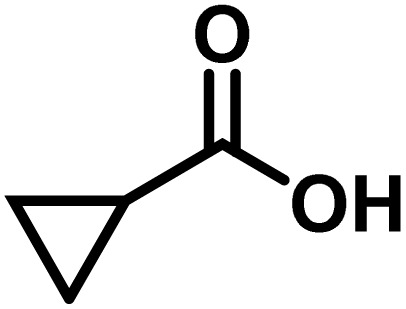	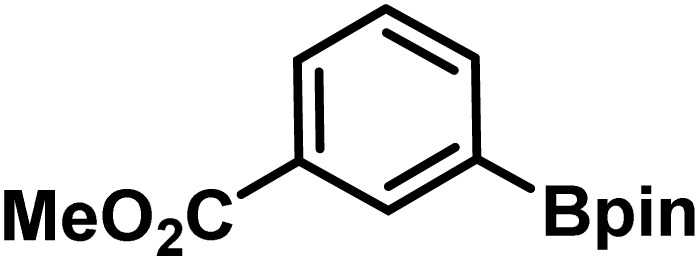	L(Ac-Phe-OH)	RC3	86±3	89

a Ar^1^ = –*p*-CF_3_C_6_F_4_; Ar^2^ = –*p*-CNC_6_F_4_.

b RC1 = Pd(MeCN)_2_Cl_2_ (10 mol%), ligand (10 mol%), Ag_2_CO_3_ (2.0 equiv.), base (2.0 equiv.), CHCl_3_ (1.0 mL), 80 °C, and 24 h. RC2 = Pd(OAc)_2_ (10 mol%), ligand (11 mol%), Ag_2_CO_3_ (1.5 equiv.), Na_2_CO_3_ (2.0 equiv.), BQ (0.5 equiv.), H_2_O (5.0 equiv.), *t*-AmylOH (0.5 mL), 70 °C, and 24 h. RC3 = Pd(OAc)_2_ (10 mol%), ligand (20 mol%), Ag_2_CO_3_ (1.5 equiv.), K_2_HPO_4_ (1.5 equiv.), *t*-BuOH (1.0 mL), H_2_O (10.0 equiv.), BQ (0.5 equiv.), 80 °C, and 12 h. RC4 = Pd(OAc)_2_ (10 mol%), ligand (20 mol%), Ag_2_CO_3_ (1.5 equiv.), Na_2_CO_3_ (2.0 equiv.), HFIP (0.25 mL), 80 °C, and 16 h.

c The standard deviation in the predicted values stems from the use of our EnP regressor, where each reaction is predicted by multiple regressors (see Fig. 2a).

It can be seen from Table 1 that the agreement between the ML-predicted %*ee* for the generated reactions and those obtained from our wet-lab experiments is very good. Predictions on both cycloalkane carboxylic acids (entries 1 through 7) and *N*-aryl amides (entries 8 through 11) can be regarded as excellent on pragmatic grounds, as most entries are well within 10 units of the actual values obtained in our prospective validation. Gratifyingly, even the generated reactions in the lower %*ee* (entries 10 and 11) are in good agreement with the experimental values. A higher standard deviation in the predicted values for these reactions is an indication of the differences in the predictions across different regressors used in our EnP model, built on fewer training samples in the low %*ee* regime.

**Fig. 7:**
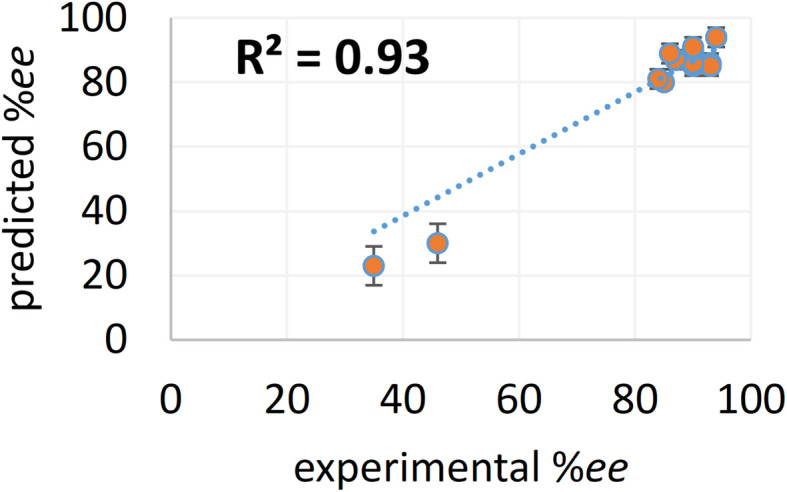
Parity plot between experimental andthe EnP predicted %*ee* for the generated reactions. Error bars represent standard deviation in the predicted values obtained from all the 30 models in the EnP regressor.

In addition to proposing new reactions given by the generated chiral ligands, we have also evaluated the quality of our EnP regressor on another smaller set of unseen reactions. Here, we have conducted new wet-lab experiments by using L(Ac-Phe-OH) as the chiral ligand reported earlier,^67^ but with different substrate and/or coupling partners, which were not previously used with this ligand. We denote these as ‘complementary reactions’ (or unexplored reactions) as their products could have been obtained by employing the known chiral ligands and a suitable choice of substrate and coupling partner. The experimentally obtained %*ee* for this family of reactions from the complementary space, shown in entries 12 through 15, also display excellent match with those predicted by the EnP regressor. Moreover, the good correlation between the predicted and experimental %*ee* for all these 15 out-of-bag reactions is evidenced by the low RMSE of 6.42 and a high *R*^2^ of 0.93 shown in Fig. 7. In contrast, DNN, RF, AttentiveFP, and T5Chem exhibited lower *R*^2^ values (0.90, 0.84, 0.88, and 0.83, respectively) and higher RMSEs, reflecting their reduced reliability in capturing experimental trends (see Table S24 in the ESI† for more details). These results highlight the robustness and potential of our TL-based ensemble approach as a reliable tool for enantioselectivity prediction in asymmetric catalysis.

**Fig. 8:**
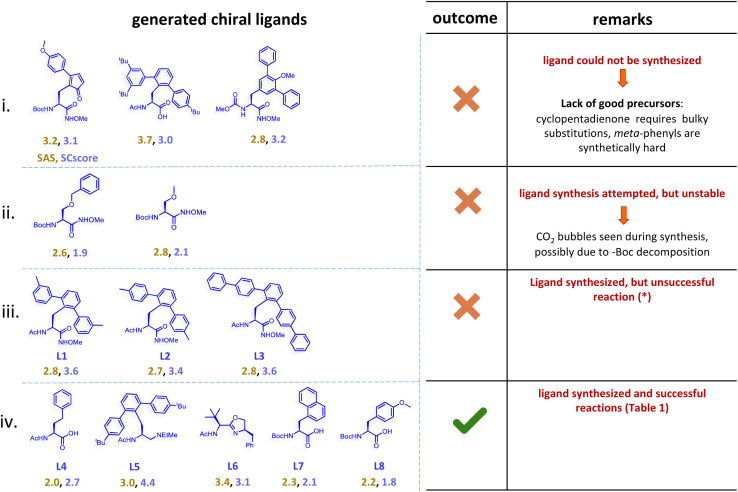
Different situations encountered in real world experimental validation of the ML-generated reactions. (*) Reaction between cyclopropane carboxylic acid and Ph-Bpin in the presence of L1 under RC3 reaction conditions led to no product formation.

Now, we shift focus from our successful validation experiments to another set of generated reactions, as shown in Fig. 8. We wish to convey that a guarded optimism would perhaps be more meaningful when it comes to the prospects of ML-driven experiments to deliver and that the involvement of domain experts in critical decisions would remain all the more important. First, consider one among the generated ligands L5 (MPAAM class) bearing a tertiary -N(Me)(Et) group, with a predicted %*ee* of 94±3 (see entry 1 in Table 1) and an experimental value of 94 obtained in this study. However, another related ligand L1, with a –(C=O)NH(OMe) group, shown as category (iii) in Fig. 8, failed to yield any product under the chosen reaction condition. This alludes to certain interesting points to consider while granting a forward pass for wet lab validation. As described earlier (also see Section 9 in ESI†), upon generation of a new chiral ligand, it is suitably combined with other species such as the transition metal (leading to a chiral transition metal catalyst), substrate, coupling partner as well as entities that contribute to the reaction (solvent, base, and additive), before passing them through the regressors for %*ee* predictions. In the case of L5, the ML-based regressor identified RC3 or RC4 as a better reaction condition with a predicted %*ee* of 94±3/93±3. Note that the efficacy of each of the generated chiral ligands is evaluated across all the other participating species (transition metal precursor, substrate, coupling partner, additive, base, *etc.*). Our wet-lab efforts with L1, a structural analog of L5 (differing in the presence of –(C=O)NH(OMe) in place of –CH_2_N(Et)Me on one donor site), however, did not give any product, serving as a clue for re-thinking on a rather liberal forward pass as adopted here. Interestingly, the chiral ligands bearing –(C=O)NH(OMe) moiety, although were found to be effective in other reactions with different substrates and reaction conditions,^44^ it failed to produce the product. It is possible that the ligands (L1–L3) itself are not suitable for catalyst formation under the chosen reaction condition or that the specific substrate used does not interact optimally with the catalytic pocket provided by the chiral ligand.

Another alternative to gather additional credence to the generative tasks is to evaluate the generated ligands for their synthetic feasibilities using metrics such as SAS and SCS.^63,64^ Values of SAS ≤ 6 and SCS ≤ 3 are generally considered good for amenable laboratory synthesis. The generated ligands (first-row in Fig. 8) have SAS in the range of 2.8 to 3.2 and SCS in 3.0 to 3.2 window. Despite these low scores, synthesis of some of the ligands was found to be not quite feasible owing to the unavailability of (or unstable) precursors. Other examples of hydroxamic acid ligands derived from Boc-*O*-benzyl-l-serine as well as Boc-*O*-methyl-l-serine resulted in decomposition during our attempt to synthesize them, despite their low SAS and SCS values (second-row Fig. 8). These might be due to Boc decomposition under the reaction condition as employed. On the contrary, some MPAHA (L1–L3) ligands could be synthesized within about six steps despite their relatively higher SCS (3.4-3.6) (see Section 3.1 in ESI†). More importantly, their low SAS did not align with the experimental observations. So far as those ligands that could be synthesized and did serve as effective catalysts in this reaction, such as the three MPAA ligands (L4, L7, and L8), possesseda low SAS (2.0-2.2) and SCS (1.8-2.7). For ligands such as L5 and L6, respectively requiring 6 and 3 steps from the commercially available precursors, the SCS (4.4 and 3.1) indeed captured the difficulty level in their synthesis. We believe that the difference in SAS/SCS depends on the molecules belonging to a given family of compounds. The SCS appears to be a slightly more reliable indicator of the ease of synthesis, at least in the present case of chiral mono-protected amino acid ligands.

All the above-mentioned factors highlight the importance of expert opinion in the initial selection from among the generated chiral ligands prior to prospective experimental validation. Domain experts can help identify and exclude ligands, despite promising ML predictions, that are synthetically challenging or unlikely to succeed under experimental conditions. For example, ligands with unstable functional groups or borderline SC scores could be discarded. Furthermore, a domain expert could as well consider the latent structural features of the successful ligands from the available known experiments to make an informed decision. This might involve an assessment of favorable steric and/or electronic characteristics and reactive moieties as suitable for the reaction conditions, to guide the selection of promising candidates.

## Conclusions

An effective transfer learning model, built on recurrent neural network framework with chemical language model, has been developed to predict the enantioselectivity (in %*ee*) of a catalytic asymmetric β-C(sp^3^)–H bond activation reaction. The reaction between substituted cycloalkanes and aryl halides, involves the use of a transition metal catalyst precursor in conjunction with a chiral (amino acid) ligand, solvent, base, and an additive. The presence of the chiral ligand facilitates the formation of a new stereogenic carbon atom in the arylated product. While this reaction serves as a prototypical example of sparse data distribution with skewness toward higher %*ee* values, our model can be deployed to develop different kinds of reactions. Our Ensemble prediction (EnP) model, consisting of 30 independent fine-tuned DL models, offered good test accuracies in %*ee* predictions for the previously reported examples using which the model is built. The EnP model is found to be more reliable for the holdout sets and bears minimal overfitting (RMSE of 6.8 (train) and 7.8 (test)) as compared to a fully trained regressor (RMSE of 6.1 (train) and 9.3(test)).

The use of another CLM (FnG), fine-tuned on 77 known chiral ligands, is found to be effective in generating novel chiral ligands for catalytic β-C(sp^3^)–H bond activation reactions. Exploration of the latent space of this FnG helped us identify several interesting and realistic chiral ligands in the neighborhood of the training samples. Several such generated ligands have good synthetic accessibility as well as synthetic complexity scores, in addition to exhibiting high novelty and uniqueness. Motivated by these, we have subjected a handful of the generated reactions to prospective wet-lab validation. The %*ee* predicted by our EnP model for the new reactions employing these ML-generated chiral ligands are found to be in very good agreement with the actual experimental values. The predictions on such unseen reactions, for both the high and low %*ee* regions, are in concert with the ground truths, thus engendering our EnP model with high practical utility. Given that in the reaction development phase, the number of reactions as well as the corresponding %*ee*s is often low, an ML intervention might be beneficial.

While most of our prospective validation experiments are highly successful, conveying that ML could guide reaction development, the results also point to the importance of domain experts in the loop. A few of the ML-generated ligands considered for experimental validation, despite their promising synthetic accessibility score and high predicted %*ee*, are found to be not viable due to a lack of readily available precursors and/or product formation. As with any predictive science, we expect that one might experience co-lateral issues beyond the scope of current ML implementations, when predictions are subjected to wet-lab validation.

## Author contributions

A. H. and R. B. S. designed machine learning research, A. H. performed the ML experiments, T. C. and J. Q. Y. designed experiments and T. C. carried out the experiments. R. B. S. wrote the manuscript with contributions from A. H.

## Conflicts of interest

The authors declare no competing financial interest.

## Supplementary Material

SC-OLF-D5SC01098E-s001

## Data Availability

The data sets and source code utilized for training the models are accessible on GitHub: https://github.com/alhqlearn/EnsembleAC

## References

[cit1] Blakemore D. C., Castro L., Churcher I., Rees D. C., Thomas A. W., Wilson D. M., Wood A. (2018). Nat. Chem..

[cit2] Taylor C. J., Pomberger A., Felton K. C., Grainger R., Barecka M., Chamberlain T. W., Bourne R. A., Johnson C. N., Lapkin A. A. (2023). Chem. Rev..

[cit3] McMullen J. P., Jensen K. F. (2010). Annu. Rev. Anal. Chem..

[cit4] Leardi R. (2009). Anal. Chim. Acta.

[cit5] Kearnes S. M., Maser M. R., Wleklinski M., Kast A., Doyle A. G., Dreher S. D., Hawkins J. M., Jensen K. F., Coley C. W. (2021). J. Am. Chem. Soc..

[cit6] Raghavan P., Haas B. C., Ruos M. E., Schleinitz J., Doyle A. G., Reisman S. E., Sigman M. S., Coley C. W. (2023). ACS Cent. Sci..

[cit7] Todd M. H. (2005). Chem. Soc. Rev..

[cit8] Williams W. L., Zeng L., Gensch T., Sigman M. S., Doyle A. G., Anslyn E. V. (2021). ACS Cent. Sci..

[cit9] Sigman M. S., Harper K. C., Bess E. N., Milo A. (2016). Acc. Chem. Res..

[cit10] Shields B. J., Stevens J., Li J., Parasram M., Damani F., Alvarado J. I. M., Janey J. M., Adams R. P., Doyle A. G. (2021). Nature.

[cit11] Wu Z., Ramsundar B., Feinberg E. N., Gomes J., Geniesse C., Pappu A. S., Leswing K., Pande V. (2018). Chem. Sci..

[cit12] Mater A. C., Coote M. L. (2019). J. Chem. Inf. Model..

[cit13] Caldeweyher E., Elkin M., Gheibi G., Johansson M., Sköld C., Norrby P.-O., Hartwig J. F. (2023). J. Am. Chem. Soc..

[cit14] Nippa D. F., Atz K., Hohler R., Müller A. T., Marx A., Bartelmus C., Wuitschik G., Marzuoli I., Jost V., Wolfard J., Binder M., Stepan A. F., Konrad D. B., Grether U., Martin R. E., Schneider G. (2023). Nat. Chem..

[cit15] Häse F., Roch L. M., Aspuru-Guzik A. (2019). Trends Chem..

[cit16] Shevlin M. (2017). ACS Med. Chem. Lett..

[cit17] Ahneman D. T., Estrada J. G., Lin S., Dreher S. D., Doyle A. G. (2018). Science.

[cit18] Zahrt A. F., Henle J. J., Rose B. T., Wang Y., Darrow W. T., Denmark S. E. (2019). Science.

[cit19] Beker W., Gajewska E. P., Badowski T., Grzybowski B. A. (2018). Angew. Chem., Int. Ed..

[cit20] Schwaller P., Vaucher A. C., Laino T., Reymond J.-L. (2021). Mach. Learn. Sci. Technol..

[cit21] Li S.-W., Xu L.-C., Zhang C., Zhang S.-Q., Hong X. (2023). Nat. Commun..

[cit22] Singh S., Sunoj R. B. (2023). Acc. Chem. Res..

[cit23] Singh S., Pareek M., Changotra A., Banerjee S., Bhaskararao B., Balamurugan P., Sunoj R. B. (2020). Proc. Natl. Acad. Sci. U. S. A..

[cit24] Das M., Sharma P., Sunoj R. B. (2022). J. Chem. Phys..

[cit25] DL models with complex model architectures are generally subjected to full training or fine-tuning using a single seed. A trained DL model is often used directly to predict on the holdout/unseen dataset

[cit26] Kwon Y., Lee D., Choi Y.-S., Kang S. (2022). J. Cheminf..

[cit27] Crabtree R. H. (2010). Chem. Rev..

[cit28] Dutta U., Maiti S., Bhattacharya T., Maiti D. (2021). Science.

[cit29] Chen X., Engle K. M., Wang D.-H., Yu J.-Q. (2009). Angew. Chem., Int. Ed..

[cit30] Gutekunst W. R., Baran P. S. (2011). J. Am. Chem. Soc..

[cit31] Virelli M., Wang W., Kuniyil R., Wu J., Zanoni G., Fernandez A., Scott J., Vendrell M., Ackermann L. (2019). Chem.–Eur. J..

[cit32] Wang D.-H., Wasa M., Giri R., Yu J.-Q. (2008). J. Am. Chem. Soc..

[cit33] Maetani M., Zoller J., Melillo B., Verho O., Kato N., Pu J., Comer E., Schreiber S. L. (2017). J. Am. Chem. Soc..

[cit34] Shalit Peleg H., Milo A. (2023). Angew. Chem., Int. Ed..

[cit35] Saebi M., Nan B., Herr J. E., Wahlers J., Guo Z., Zurański A. M., Kogej T., Norrby P.-O., Doyle A. G., Chawla N. V., Wiest O. (2023). Chem. Sci..

[cit36] We have performed statistical analyses to quantify the skewness in our β-C(sp^3^)–H bond activation reaction dataset. Skewness measures the asymmetry in a probability distribution with respect to the mean. A skewness 0 indicates a symmetric distribution, while a positive or a negative value, respectively, suggests a right- or a left-skewed distribution. The skewness in the ELN (749 reactions) (ref. 35), and NiCOlit (1813 reactions) (ref. 37) datasets are, respectively, 0.16 and 0.44. In contrast, the β-C(sp^3^)–H dataset (220 reactions) exhibited a negative skewness of −2.30. More details of % yield/%*ee* distribution across all datasets can be found in Section 14 of the ESI.†

[cit37] Schleinitz J., Langevin M., Smail Y., Wehnert B., Grimaud L., Vuilleumier R. (2022). J. Am. Chem. Soc..

[cit38] Hosna A., Merry E., Gyalmo J., Alom Z., Aung Z., Azim M. A. (2022). J. Big Data.

[cit39] Anand M., Sunoj R. B., Schaefer III H. F. (2014). J. Am. Chem. Soc..

[cit40] Reddi Y., Sunoj R. B. (2015). ACS Catal..

[cit41] Zhang T., Zhang Z.-Y., Kang G., Sheng T., Yan J.-L., Yang Y.-B., Ouyang Y., Yu J.-Q. (2024). Science.

[cit42] Strassfeld D. A., Chen C.-Y., Park H. S., Phan D. Q., Yu J.-Q. (2023). Nature.

[cit43] Hoque A., Sunoj R. B. (2022). Digital Discovery.

[cit44] Shen P.-X., Hu L., Shao Q., Hong K., Yu J.-Q. (2018). J. Am. Chem. Soc..

[cit45] Hu L., Shen P.-X., Shao Q., Hong K., Qiao J. X., Yu J.-Q. (2018). Angew. Chem., Int. Ed..

[cit46] Xiao K.-J., Lin D. W., Miura M., Zhu R.-Y., Gong W., Wasa M., Yu J.-Q. (2014). J. Am. Chem. Soc..

[cit47] Wu Q.-F., Wang X.-B., Shen P.-X., Yu J.-Q. (2018). ACS Catal..

[cit48] Howard J. and Ruder S., in Proceedings of the 56th Annual Meeting of the Association for Computational Linguistics (Volume 1: Long Papers), Association for Computational Linguistics, Stroudsburg, PA, USA, 2018 10.18653/v1/2024.acl-long.199 PMC12007664 40255468

[cit49] Singh S., Sunoj R. B. (2022). Digital Discovery.

[cit50] Li J., Tang T., Zhao W. X., Nie J.-Y., Wen J.-R. (2024). ACM Comput. Surv..

[cit51] Comparison of model performance, with and without transfer learning (TL), revealed that the use of TL for the small target task situations as in the present case is beneficial.Statistical significance test yielded low *p*-values (< 0.05) when the test RMSEs obtained from TL and no-TL models were compared.See Section 4 in the ESI.†

[cit52] Singh S., Sunoj R. B. (2022). iScience.

[cit53] In our ensemble prediction (EnP) approach, we chose 70% of the reaction dataset using controlled randomization. This has been done by employing (1) a fixed initial seed (seed = 42) to establish baseline consistency and (2) distinct secondary seeds (seed = 100, 200, *etc.*) to generate varied training sets and parameter initializations for each model (M1 to M30). This dual-seed strategy helps in reproducing (through the initial seed) as well as model diversity (*via* subsequent random seeds). This approach imparts better robustness and generalization abilities to the models as it leverages diverse training subsets. See more details about the model training, validation and test in Table S6 in the ESI.†

[cit54] Ganaie M. A., Hu M., Malik A. K., Tanveer M., Suganthan P. N. (2022). Eng. Appl. Artif. Intell..

[cit55] While the term ‘out-of-bag’ (OOB) is widely found in the ML literature, it can be considered analogous to ‘unseen’ reactions as well

[cit56] In addition to predicting %*ee*, we derived ΔΔ*G*^‡^ values from the reported %*ee* and used them as target variables. Our comparative analysis demonstrated that the EnP model, when trained on %*ee*, delivered superior predictive performance (*R*^2^ = 0.64) compared to when it was trained on ΔΔ*G*^‡^ (*R*^2^ = 0.53) (see Table S25†). These results indicate that the EnP model more effectively captures trends in enantioselectivity when it learns directly from %*ee* rather than on the ΔΔ*G*^‡^ values

[cit57] Xiong Z., Wang D., Liu X., Zhong F., Wan X., Li X., Li Z., Luo X., Chen K., Jiang H., Zheng M. (2019). J. Med. Chem..

[cit58] To ensure fairness in evaluation, an efficient and systematic hyperparameter tuning across these models was performed using optuna. Optuna is a python-based code that enables construction of the parameter search space and uses a search and prune strategy. A detailed comparison is provided in Tables S6, S20–S22 in the ESI.†

[cit59] Lu J., Zhang Y. (2022). J. Chem. Inf. Model..

[cit60] McInnes L., Healy J., Saul N., Großberger L. (2018). J. Open Source Softw..

[cit61] Different unsupervised dimensionality reduction techniques are employed for various purposes. UMAP helps in visualizing high-dimensional data and could provide insights into how the encoder learns different types of reactions. It helped us understand whether certain clusters in the UMAP plot correspond to specific substrates and chiral ligands (Fig. 3)

[cit62] Shao Q., Wu K., Zhuang Z., Qian S., Yu J.-Q. (2020). Acc. Chem. Res..

[cit63] Xing Y.-Y., Liu J.-B., Sun Q.-M., Sun C.-Z., Huang F., Chen D.-Z. (2019). J. Org. Chem..

[cit64] Jensen J. H. (2019). A graph-based genetic algorithm and generative model/Monte Carlo tree search for the exploration of chemical space. Chem. Sci..

[cit65] Guo M. , Thost V., Song S. W., Balachandran A., Das P., Chen J. and Matusik W., International Conference on Learning Representations, 2021

[cit66] FCD quantifies how closely generated molecules align with the real ones by comparing their distributions, with lower values indicating higher chemical resemblance

[cit67] TMAP is a tree-like representation that helped us effectively convey how the generated ligands (*i.e.*, exploration) compare with those in the training set ligands. This plot therefore provides a chemically interpretable comparison between the two kinds of ligands

[cit68] Bajusz D., Rácz A., Héberger K. (2015). J. Cheminf..

[cit69] Park Y., Niemeyer Z. L., Yu J.-Q., Sigman M. S. (2017). Organometallics.

[cit70] Ertl P., Schuffenhauer A. (2009). J. Cheminf..

[cit71] Coley C. W., Rogers L., Green W. H., Jensen K. F. (2018). J. Chem. Inf. Model..

[cit72] We used t-SNE as it prioritizes local clustering that helps in intuitive selection of high %*ee* reactions. The plot clearly separates meaningful reaction groups in latent space

[cit73] In the t-SNE plots, the numbers along the axes (*e.g.*, t-SNE1 and t-SNE2) represent the coordinates of data points in the 2D space generated by the t-SNE algorithm. These coordinates are derived from 400 high-dimensional latent vectors and are designed to preserve the relative distances between data points while reducing dimensionality for visualization purposes. The values on the t-SNE axes do not have a direct physical interpretation in relation to the original high-dimensional vectors but are crucial for understanding the clustering and relationships within the data. These coordinates help identify areas in the latent space where reactions are more likely to achieve higher %*ee*, guiding the selection of promising candidates for further validation or exploration

