# Supplementary material for: Molecular Machine Learning Approach to Enantioselective C–H Bond Activation Reactions: From Generative AI to Experimental Validation
Source: Chem Sci. 2025 Jun 10;16(29):13276–90. doi: 10.1039/d5sc01098e (PMC12184194; doi:10.1039/d5sc01098e)
Supplement: SC-016-D5SC01098E-s001 [file SC-016-D5SC01098E-s001.pdf]

Supplementary Information (SI) for Chemical Science.
This journal is © The Royal Society of Chemistry 2025

Electronic Supplementary Information

for

Molecular Machine Learning Approach to Enantioselective C–H Bond Activation Reactions: From Generative AI to Experimental Validation

Ajnabiul Hoque,[a] Taiwei Chang,[b] Jin-Quan Yu,[b,*] and Raghavan B. Sunoj[a,c,*]

[a] Department of Chemistry, Indian Institute of Technology Bombay, Powai, Mumbai 400076, India

[b] Department of Chemistry, The Scripps Research Institute, La Jolla, California 92037, United States

[c] Centre for Machine Intelligence and Data Science, Indian Institute of Technology Bombay, Powai, Mumbai 400076, India

E-mail: yu200@scripps.edu, sunoj@chem.iitb.ac.in

Part I

Table of Contents

section		Content	page number
1		Details of all reaction components	S3-S5
	1.1	Chiral Ligand	
	1.2	Coupling partner	
	1.3	Substrate	
	1.4	Base	
	1.5	Transition metal catalyst precursor	
	1.6	Solvent	
	1.7	Additive	
2		Overview of ULMFiT	S5-S10
	2.1	General-domain chemical language model pre-training	
	2.2	Fine-tuning of target task regressors	
	2.3	Fine-tuning of chemical language model with chiral ligands	
3		Hyperparameter optimization for the target task regressor	S10-S13
4		Hyperparameter optimization for the target task-regressor without transfer learning	S13-S15
5		Analysis of encoder output of the fine-tuned regressor	S15-S20
6		Hyperparameter tuning for the target task generator	S20-S22
7		Details of generated chiral ligands	S22-S28
8		Comparison between generated and experimental ligands	S28-S30
9		Reaction space using generated ligands	S30-S31
10		Performance comparison of different types of regressors	S32-S37
11		Various benchmark models for the generative tasks	S37-S39
12		Choice of %*ee* versus $\Delta\Delta G^{\ddagger}$ in modeling enantioselectivity	S39
13		Experimental vs. EnP predicted %*ee* parity plot performance	S39-S40
14		Distribution of %yield/%*ee*	S41-S42
15		References	S42-S44

(1) Details of all reaction components

(1.1) Ligands

Our initial dataset consists of previously reported experiments (refs. [1], [2], [3], [4]). There are 77 chiral ligands in this dataset, which can be classified into four categories: (a) mono-N-protected amino acid (MPAA) [**L**$_{\mathbf{A}}$], (b) mono-N-protected α-amino-O-alkyl hydroxamic acid (MPAHA) [**L**$_{\mathbf{B}}$], (c) mono-N-protected amino-alkyl amine (MPAAM) [**L**$_{\mathbf{C}}$], and (d) N-acyl-protected amino oxazoline (APAO) [**L**$_{\mathbf{D}}$].

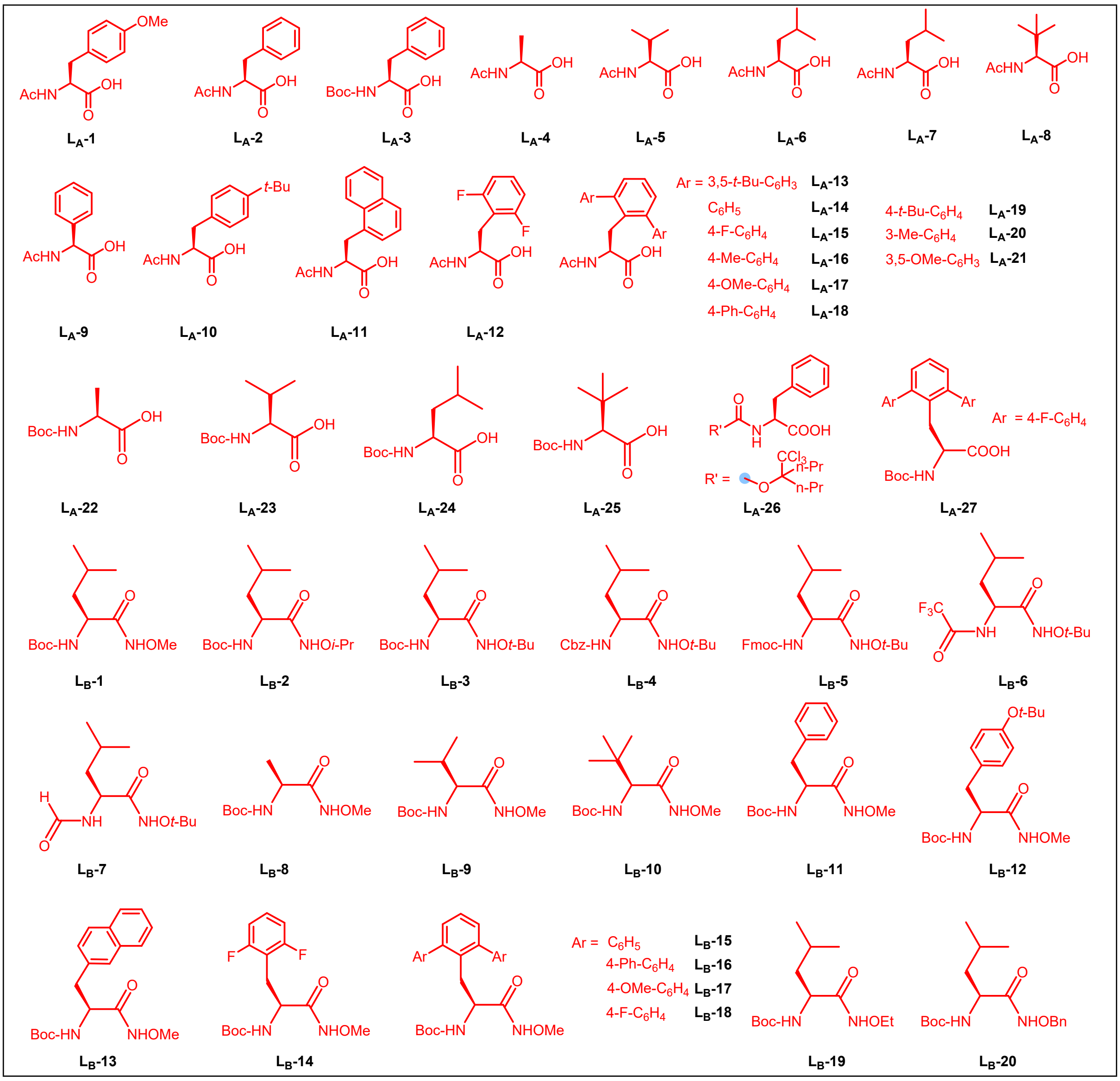

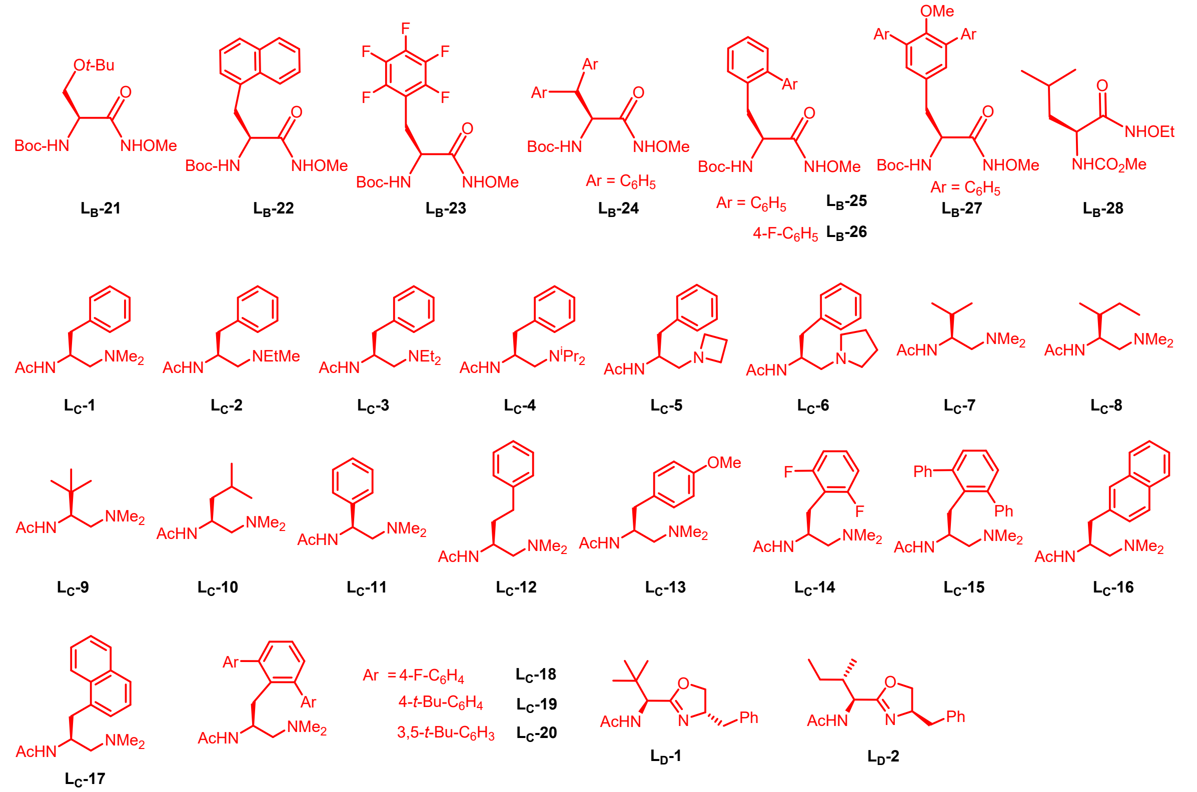

Fig. S1. Notations used for the 77 chiral ligands in our manually curated dataset.

(1.2) Coupling partners

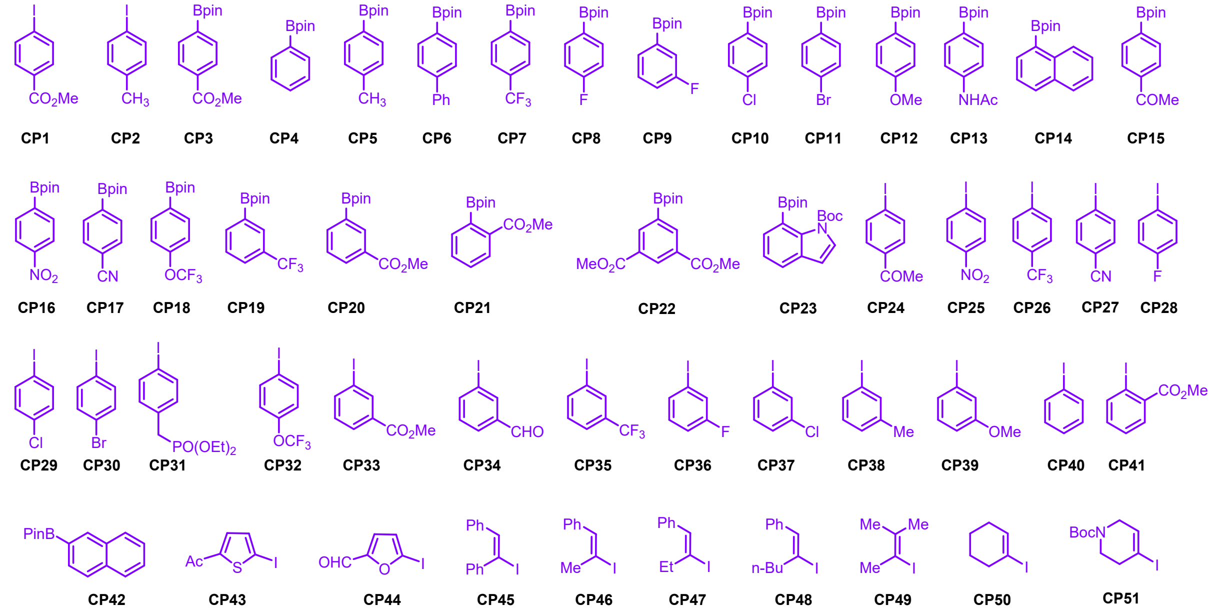

Fig. S2. Notations used for the 51 coupling partners in our manually curated dataset.

(1.3) Substrates

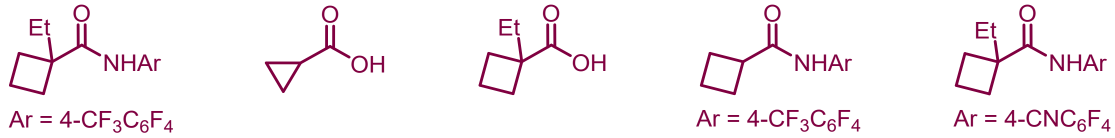

S1 **S2** **S3** **S4** **S5**

Fig. S3. Notations used for the 5 cyclopropyl and cyclobutyl substrates in our manually curated dataset.

(1.4) Bases

NaTFA, Na_2CO_3, $NaHCO_3$, K_2HPO_4, Li_3PO_4, Na_3PO_4, K_3PO_4, LiH_2PO_4, Li_2CO_3, K_2CO_3, Cs_2CO_3, LiOAc, NaOAc, KOAc, CsOAc, NaH_2PO_4, Na_2HPO_4, $KHCO_3$, KH_2PO_4, $K_2HPO_4.3H_2O$

(1.5) Pd catalyst precursors

$Pd(MeCN)_2Cl_2$, $Pd(TFA)_2$, $Pd(C_3H_5)Cl_2$, $Pd(PhCN)_2Cl_2$, $Pd(OTf)_2(MeCN)_4$, $Pd(OAc)_2$, $Pd(BF_4)_2(MeCN)_4$, $Pd(PPh_3)_2Cl_2$, $Pd(OPiv)_2$

(1.6) Solvents

Toluene, $CHCl_3$, HFIP, *t*-AmylOH + H_2O, *t*-BuOH + H_2O, *i*-PrOH + H_2O, *i*-BuOH + H_2O, THF + H_2O, HFIP + H_2O, *t*-AmylOH, DCE, DCM, DMF, C_6F_6, TBME, MeCN, THF, Dioxane, Et_2O, CCl_4

(1.7) Additives

Ag_2CO_3, AgOAc, Ag_2O

2. Overview of ULMFiT

Our approach leverages the Universal Language Model Fine-Tuning (ULMFiT) strategy, developed by Howard and Ruder, which can enable efficient and effective transfer learning (TL) for various NLP tasks, including text classification, sentiment analysis, and language modelling.[5] This approach achieves high performance across a range of tasks with minimal labeled data. Inspired by the success of language models,[6] we adapted the ULMFiT for the molecular domain by utilizing SMILES (Simplified Molecular Input Line Entry System)

strings.[7,8,9,10] Our approach consists of two stages: (a) pre-training, the model is pre-trained on general-domain data to learn universal representations, and (b) fine-tuning, the pre-trained model is fine-tuned for a specific task in the molecular domain, enabling the model to learn task-specific patterns and relationships.

(2.1) General-domain CLM pre-training

The Chemical Language Model (CLM) is trained on a large dataset of ~1M unlabeled molecules from the ChEMBL dataset represented first in the form of the corresponding SMILES strings. SMILES is a one-line textual representation of a molecule, using atomic symbols (e.g., C for carbon and O for oxygen) to denote atoms and implying bonds between them based on their order. Additionally, SMILES notation indicates branching with parentheses and ring structures with numbers, showing bond connections between atoms. To input molecules in the form of the SMILES strings, we first divide the SMILES into individual characters or tokens, each representing a single atom or specific chemical environment (e.g., [OH-] and stereochemical information). Each token is then converted into a unique integer using a string-to-integer dictionary, which contains N entries, including the beginning of string (BOS) and the end of string (EOS) tokens to indicate the start and end of each SMILES string, respectively.

This pre-training step enables the model to learn molecular structure and connections, as well as SMILES grammar and meaning, by predicting the next character. We used AWD-LSTM (Long-Short Term Memory) as the core architecture, which is a type of recurrent neural network (RNN) capable of handling sequential data and learning both the short- and long-term dependencies within a sequence. This architecture consists of an embedding layer, an encoder with three LSTM layers, and a decoder layer. The embedding layer converts numerical tokens into 400-dimensional real-valued vectors, representing each character in the SMILES string in a 400-dimensional space (see Fig. S4). These vectors capture the semantic

relationships among the characters. The output from the embedding layer is fed into the first of the three LSTM layers in the encoder, each with 1152 hidden units. The output of the final LSTM layer is decoded by a fully connected linear layer, followed by a softmax function, which assigns the probability for every token in the vocabulary to be the next token.

The pre-training dataset typically encompasses a broad range of the chemical space, but the target task may exhibit a distinct distribution. To leverage the knowledge acquired during pre-training, the pre-trained weights are used to fine-tune the target task by adopting a TL approach. This enables the pre-trained model to adapt to the new task while retaining the valuable information learned during pre-training. This pre-trained model is then used for the following two different downstream tasks.

(2.2) Fine-tuning of target task regressors

Our target task involves predicting the %*ee* of asymmetric β-C(sp^3) H bond activation reactions, which is a regression problem. In this phase, we utilize the embedding layer of the pre-trained model and three LSTM layers while modifying the decoder with the removal of softmax layers for the regression tasks (shown in Fig. S4). To generate a feature vector for the decoder unit, we employ concat pooling by concatenating the last hidden state with max-pooled and mean-pooled representations of all the hidden states in the third LSTM layer, resulting in a 1200-dimensional vector for each character. This vector is then passed to a feed-forward neural network serving as the linear decoder, comprising two linear layers with 50 activations and a final output dimension of 1 for regression. The two linear layers of the regressor are initialized with random weights and trained from scratch. Fine-tuning the regressor is crucial for effective TL. To achieve this, we implement a gradual unfreezing protocol, allowing the model to adapt to the target task while retaining the knowledge gained during pre-training.

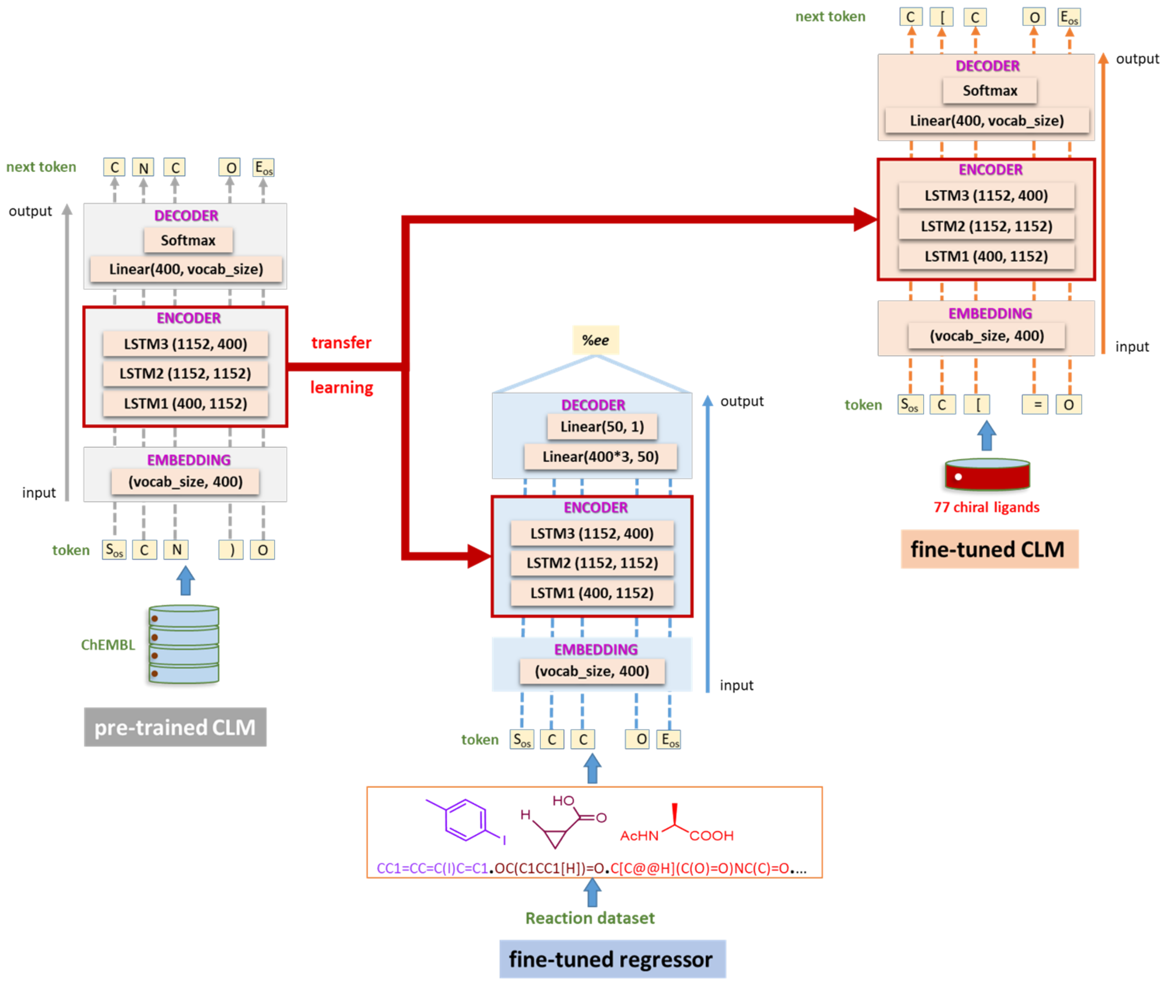

Fig. S4. Overview the ULMFiT method for pre-training of general domain CLM and use of this model for fine tuning two downstream tasks, such as the building of a regression model to predict %*ee* and the generation of novel chemical entities (ligands) from a small subset of chiral ligands.

(2.3) Fine-tuning of CLM

Recognizing the significance of TL in low-data settings and the crucial role of chiral ligands in inducing chirality in products, we aim to explore the chemical space of a small dataset containing the SMILES strings of 77 chiral ligands using the TL method. Following the TL approach, we fine-tune the pre-trained CLM on this dataset to learn task-specific features. The primary objective is to adapt the model to generate SMILES strings that resemble the

ligands of interest, enabling the model to capture the unique characteristics of these chiral ligands.

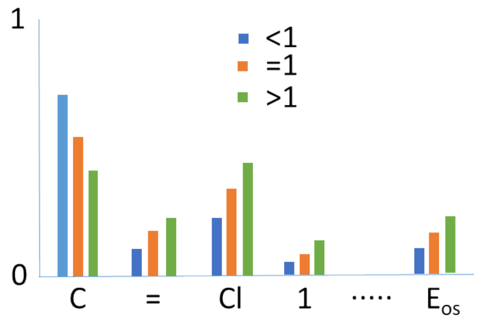

Fig. S5. Effect of temperature sampling in the generation of new molecules from the fine-tuned CLM

In LSTM-based chemical language models, random sampling methods are generally employed to introduce diversity in generated molecules.[11] The key parameter controlling this diversity is the temperature (τ), which regulates the randomness of the sampling process. The random sampling method involves selecting the next character in a sequence based on the learned probability distribution. The probability of selecting each possible next character (i) is computed using the softmax function as

$$P_i = \frac{e^{(z_i)}}{\sum_j e^{(z_j)}}$$

where, z_i is the output of the LSTM output layer, P_i is the sampling probability of token i as determined by the CLM, variable j represents all possible indices corresponding to the different characters in the vocabulary. To introduce randomness, a temperature parameter τ is applied to the logits z_i before computing the softmax probabilities as,

$$P_i = \frac{e^{(z_i/\tau)}}{\sum_j e^{(z_j/\tau)}}$$

A higher τ flattens the probability distribution, increasing the likelihood of selecting less probable characters, while a lower temperature makes the distribution more peaked, favoring more probable characters (see Fig. S5). By adjusting τ, the model can generate more diverse or more deterministic sequences, allowing for control over the trade-off between novelty and accuracy in molecule generation tasks.

3. Hyperparameter optimization for the target task regressor

Optimizing hyperparameters is crucial for fine-tuning the target-task regressor. In this case, the data is divided into 70% for training, 10% for validation, and 20% for testing. Hyperparameter tuning is carried out using the validation set, and the models are assessed using the root mean squared error (RMSE) as the error metric.

Table S1. Train and Validation RMSEs Obtained by Varying Number of Augmented SMILES.[i] Shown in Bold Font is the Optimal Model

No. of augmented SMILES	train	validation
0	81.52	81.81
25	7.69	9.16
50	7.47	9.87
**100**	**7.25**	**8.78**
200	7.45	9.25
300	7.20	9.32

[i] seed =1234, σ_g = 0.0, batch size = 128, dropout ratio = 0.0, epoch = [6, 7, 7, 7], learning rate = [3e-2, slice(5e-4/(2.6^4),5e-3), slice(5e-4/(2.6^4),5e-3), (5e-6/(2.6^4),5e-5)]. The `slice()` function defines a range of values and is commonly used to assign distinct learning rates to different layers in a deep learning model.

Table S2. Train and Validation RMSEs Obtained by Varying the Noise in the Experimental %*ee* (σ_g).[i] Shown in Bold Font is the Optimal Model

σ_g	train	validation
0.0	7.25	8.78
0.1	7.27	8.61
**0.3**	**7.20**	**7.89**
0.5	7.23	8.08
0.7	7.29	8.87
0.9	7.36	8.19

[i] seed =1234, augmented SMILES = 100, batch size = 128, dropout ratio = 0.0, epoch = [6, 7, 7, 7], learning rate = [3e-2, slice(5e-4/(2.6^4),5e-3), slice(5e-4/(2.6^4),5e-3),slice(5e-6/(2.6^4),5e-5)]

Table S3. Train and Validation RMSEs Obtained by Varying Dropout Rate.[i] Shown in Bold Font is the Optimal Model

dropout rate	train	validation
**0.0**	**7.20**	**7.89**
0.1	7.72	8.55
0.2	8.01	8.43
0.3	8.35	8.83
0.4	8.49	9.56
0.5	8.78	9.56
0.6	9.09	10.53
0.7	9.60	10.34
0.8	10.11	10.32
0.9	10.87	11.59

[i] seed =1234, σ_g = 0.3, augmented SMILES = 100, batch size = 128, epoch = [6, 7, 7, 7], learning rate = [3e-2, slice(5e-4/(2.6^4),5e-3), slice(5e-4/(2.6^4),5e-3), slice(5e-6/(2.6^4),5e-5)]

Table S4. Train and Validation RMSEs Obtained by Varying Number of Epochs.[i] Shown in Bold Font is the Optimal Model

epoch	train	validation
[2, 2, 2, 4]	8.42	10.67
[3, 3, 3, 4]	7.69	8.19
[3, 4, 5, 6]	7.43	8.07
[4, 4, 4, 5]	7.48	8.6
[5, 6, 6, 6]	7.07	7.93
[6, 6, 6, 6]	7.21	8.18
**[6, 7, 7, 7]**	**7.20**	**7.89**
[7, 8, 8, 8]	7.55	8.96
[8, 8, 8, 8]	7.12	8.22
[8, 9, 10, 10]	6.94	8.75
[9, 10, 10, 10]	6.75	8.17

[i] seed =1234, σ_g = 0.3, augmented SMILES = 100, batch size = 128, dropout ratio = 0.0, learning rate = [3e-2, slice(5e-4/(2.6^4),5e-3), slice(5e-4/(2.6^4),5e-3), slice(5e-6/(2.6^4),5e-5)]

Table S5. Train and Validation RMSEs Obtained by Varying Learning Rate.[i] Shown in Bold Font is the Optimal Model

learning rate	train	validation
[0.01, 0.01, 0.01, 0.001]	6.80	9.94
[0.01, 0.01, 0.01, 0.0001]	6.89	9.97
[0.01, 0.01, 0.001, 1e-05]	6.93	10.55
[0.145, 0.01, 0.001, 0.0001]	7.02	9.84
[0.145, 0.001, 0.001, 0.001]	7.24	8.21
[0.01, 0.001, 0.0001, 1e-05]	7.55	8.63
[3e-2,slice(5e-3/(2.6^4),5e-3),slice(5e-4/(2.6^4),5e-	7.20	7.89

4),slice(5e-5/(2.6^4),5e-5)]		
[3e-2,slice(5e-3/(2.6^4),5e-3),slice(5e-3/(2.6^4),5e-3),slice(5e-5/(2.6^4),5e-5)]	7.00	8.22
[3e-1,slice(5e-2/(2.6^4),5e-2),slice(5e-2/(2.6^4),5e-2),slice(5e-4/(2.6^4),5e-4)]	6.78	9.36
**[3e-2,slice(5e-4/(2.6^4),5e-3),slice(5e-4/(2.6^4),5e-3),slice(5e-6/(2.6^4),5e-5)]**	**7.10**	**7.52**
[3e-2,slice(5e-4/(2.6^4),5e-4),slice(5e-4/(2.6^4),5e-4),slice(5e-6/(2.6^4),5e-6)]	7.95	8.11

[i] seed =1234, σ_g = 0.3, augmented SMILES = 100, batch size = 128, epoch = [6, 7, 7, 7], dropout ratio = 0.0

Table S6. Train, Validation and Test RMSEs Obtained by Varying Split.[i] Shown in Bold Font is the Optimal Model

split	train	validation	test (canonical)	test (TTA)
1	7.22	6.85	8.32	8.70
2	6.78	8.39	6.96	6.42
3	6.71	11.46	6.46	5.88
4	6.39	8.81	6.40	6.80
5	6.79	8.51	7.92	6.98
6	6.72	8.81	9.03	8.61
7	7.05	6.23	8.19	6.86
8	7.67	7.34	7.33	8.14
9	6.73	13.31	8.89	8.72
10	6.71	8.98	7.59	5.89
11	7.04	6.14	6.70	7.17
12	6.79	7.96	11.75	12.14
13	7.37	7.21	6.83	5.94
14	6.65	8.59	7.20	7.44
15	7.03	7.52	10.04	9.54
16	6.67	6.61	8.34	8.31
17	6.21	8.55	7.57	7.03
18	6.27	5.30	7.51	7.98
19	6.14	9.99	9.33	7.65
20	6.73	5.69	8.59	8.45
21	6.81	6.93	7.80	7.99
22	7.37	9.11	7.79	7.50
23	7.21	6.70	6.93	7.26
24	6.46	7.18	7.14	6.87
25	7.18	5.13	5.86	5.89
26	6.69	6.97	8.76	8.56
27	7.29	8.02	6.67	6.26
28	7.03	6.70	6.18	6.18
29	6.55	8.11	8.59	8.77

30	7.01	5.74	7.29	7.13
**avg. ± std. dev.**	**6.84±0.36**	**7.76±1.74**	**7.80±1.23**	**7.57±1.31**

[i] σ_g = 0.3, augmented SMILES = 100, batch size = 128, dropout ratio = 0.0, learning rate = [3e-2,slice(5e-4/(2.6^4),5e-3),slice(5e-4/(2.6^4),5e-3),slice(5e-6/(2.6^4),5e-5)], epoch = [6, 7, 7, 7]

4. Hyperparameter optimization for the target task-regressor without TL

Table S7. Train and Validation RMSEs Obtained by Varying Number of Augmented SMILES.[i] Shown in Bold Font is the Optimal Model

No. of augmented SMILES	train	validation
0	82.89	83.89
25	68.86	67.36
50	40.66	43.11
100	14.84	14.64
200	13.35	14.75
**300**	**6.99**	**9.73**

[i] seed =1234, σ_g = 0.0, batch size = 128, dropout ratio = 0.0, epoch = [25], learning rate = [1e-3]

Table S8. Train and Validation RMSEs Obtained by Varying σ_g.[i] Shown in Bold Font is the Optimal Model

σ_g	train	validation
**0.0**	**6.99**	**9.73**
0.1	7.00	11.58
0.3	7.03	10.19
0.5	7.01	10.50
0.7	7.03	10.17
0.9	7.06	10.34

[i] seed =1234, augmented SMILES = 300, batch size = 128, dropout ratio = 0.0, epoch = [25], learning rate = [1e-3]

Table S9. Train and Validation RMSEs Obtained by Varying Dropout Rate.[i] Shown in Bold Font is the Optimal Model

dropout rate	train	validation
0.0	6.99	9.73
**0.1**	**7.12**	**7.61**
0.2	7.40	8.57
0.3	7.23	11.36
0.4	7.29	9.24
0.5	7.38	9.92
0.6	7.45	9.46
0.7	7.62	9.83

0.8	13.30	16.14
0.9	12.73	16.90

[i] seed =1234, augmented SMILES = 300, σ_g = 0.0, batch size = 128, epoch = [25], learning rate = [1e-3]

Table S10. Train and Validation RMSEs Obtained by Varying Number of Epochs.[i] Shown in Bold Font is the Optimal Model

epoch	train	validation
15	7.87	9.90
**25**	**7.12**	**7.61**
35	6.97	8.57

[i] seed =1234, augmented SMILES = **300**, σ_g = 0.0, dropout ratio = **0.1**, batch size = 128, learning rate = [1e-3]

Table S11. Train and Validation RMSEs Obtained by Varying Learning Rate.[i] Shown in Bold Font is the Optimal Model

learning rate	train	validation
0.1	14.01	11.65
0.01	13.74	13.86
**0.001**	**7.12**	**7.61**
0.0001	69.32	69.51

[i] seed =1234, augmented SMILES = 300, σ_g = 0.0, dropout ratio = 0.1, batch size = 128, epoch = [25]

Table S12. Train, Validation, and Test RMSEs Obtained by Varying Split.[i] Shown in Bold Font is the Optimal Model

split	train	validation	test (canonical)	test (TTA)
1	7.25	6.69	8.59	8.26
2	8.67	11.22	15.83	12.91
3	7.72	11.56	7.68	6.55
4	7.14	12.62	6.92	7.52
5	6.82	10.45	6.97	7.84
6	6.41	7.17	10.34	10.42
7	6.75	6.32	7.82	7.47
8	8.25	7.95	12.25	12.75
9	6.72	14.25	10.03	9.37
10	7.14	8.62	8.10	6.96
11	6.80	11.14	7.44	7.28
12	6.99	6.85	13.21	12.65
13	7.22	7.14	6.33	6.40
14	7.13	7.84	8.44	8.81
15	8.07	6.15	12.87	12.73
16	6.48	7.71	8.17	8.08
17	5.93	9.80	5.63	5.82
18	6.62	6.00	8.47	8.66

19	6.26	8.27	7.41	5.96
20	6.90	5.91	9.10	9.38
21	6.62	8.42	8.92	7.07
22	7.82	10.35	11.10	9.97
23	6.99	8.53	9.74	9.31
24	6.93	10.79	11.11	10.24
25	7.49	6.18	6.83	7.06
26	6.35	7.80	8.98	8.68
27	7.04	5.72	6.72	6.09
28	6.65	6.95	7.40	6.22
29	6.68	4.90	8.59	9.11
30	7.13	6.66	8.53	10.04
**avg. ± std. dev.**	**7.03±0.59**	**8.33±2.26**	**8.98±2.25**	**8.65±2.08**

[i] augmented SMILES = 300, σ_g = 0.0, dropout ratio = 0.1, batch size = 128, learning rate = [1e-3], epoch = [25]

5. Analysis of the encoder output of the fine-tuned regressor

We have analyzed the final hidden state of the encoder to understand the features learned by the fine-tuned regressor. The encoder processes the input data and condenses it into a 400 dimensional fixed-length vector, capturing essential information such as contextual relationships and temporal dependencies. To visualize this high-dimensional data, we applied Uniform Manifold Approximation and Projection (UMAP) for dimensionality reduction,[12] and the *k*-means clustering of the first two principal components.

To determine the optimal number of clusters (*k*), we used the Silhouette score, a valuable metric for evaluating the quality and separation of clusters produced by the k-means clustering. The Silhouette score ranges from -1 to 1, (a) high scores (closer to 1) indicate well-separated and cohesive clusters, (b) low scores indicate overlapping or poorly defined clusters, and (c) scores around 0 indicate uncertain or noisy clusters. Fig. S6 illustrates the effect of different *k* values on clustering performance. We note that the highest Silhouette score belongs to when *k* was set to 7, revealing seven distinct groups of reactions in the training set. Further details on how various samples(reactions) are distributed among the seven clusters can be found in Table S13.

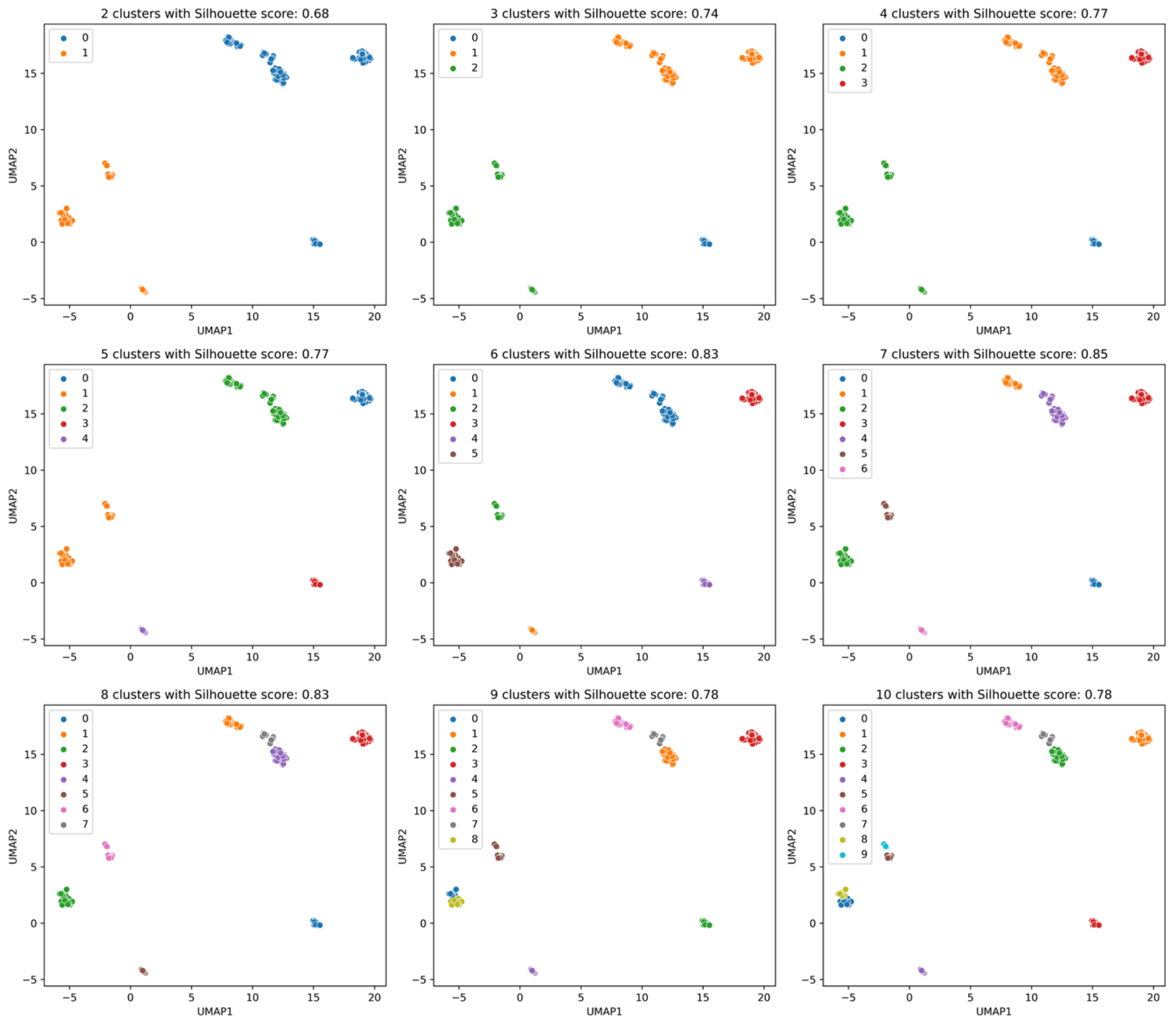

Fig. S6. *k*-means clustering of the encoder output for different values of *k*

Table S13. Identities of Samples in Different Clusters (see Fig. S1-S3 for the details of sample nomenclature)

cluster label	%*ee*	description
0	92	S4-LD-2-CP2-Pd(MeCN)$_2$(Cl)$_2$-NaOAc-Ag2CO3-CHCl3
0	92	S4-LD-2-CP2-Pd(MeCN)2(Cl)2-KOAc-Ag2CO3-CHCl3
0	80	S4-LD-2-CP2-Pd(MeCN)2(Cl)2-Cs2CO3-Ag2CO3-CHCl3
0	81	S4-LD-2-CP2-Pd(MeCN)2(Cl)2-NaTFA-Ag2CO3-CHCl3
0	88	S4-LD-2-CP2-Pd(MeCN)2(Cl)2-K3PO4-Ag2CO3-CHCl3
0	74	S4-LD-2-CP2-Pd(MeCN)2(Cl)2-K2CO3-Ag2CO3-CHCl3
0	88	S4-LD-2-CP2-Pd(MeCN)2(Cl)2-Na2HPO4-Ag2CO3-CHCl3
0	90	S4-LD-2-CP2-Pd(MeCN)2(Cl)2-Li3PO4-Ag2CO3-CHCl3
0	90	S4-LD-2-CP2-Pd(MeCN)2(Cl)2-KH2PO4-Ag2CO3-CHCl3
0	76	S4-LD-2-CP2-Pd(OTf)2(MeCN)4-NaH2PO4-Ag2CO3-CHCl3
0	82	S4-LD-2-CP2-Pd(PhCN)2(Cl)2-NaH2PO4-Ag2CO3-CHCl3
0	88	S4-LD-2-CP2-Pd(MeCN)2(Cl)2-NaHCO3-Ag2CO3-CHCl3

0	90	S4-LD-2-CP2-Pd(MeCN)2(Cl)2-NaH2PO4-Ag2CO3-CHCl3
1	82	S3-LC-15-CP23-Pd(OAc)2-K2HPO4-Ag2CO3-t-BuOH_H2O
1	72	S3-LC-1-CP3-Pd(OAc)2-K2HPO4-Ag2CO3-t-BuOH_H2O
1	90	S3-LC-15-CP6-Pd(OAc)2-K2HPO4-Ag2CO3-t-BuOH_H2O
1	70	S3-LC-14-CP3-Pd(OAc)2-K2HPO4-Ag2CO3-t-BuOH_H2O
1	70	S3-LC-19-CP3-Pd(OAc)2-K2HPO4-Ag2CO3-t-BuOH_H2O
1	86	S3-LC-15-CP15-Pd(OAc)2-K2HPO4-Ag2CO3-t-BuOH_H2O
1	84	S3-LC-15-CP22-Pd(OAc)2-K2HPO4-Ag2CO3-t-BuOH_H2O
1	90	S3-LC-15-CP17-Pd(OAc)2-K2HPO4-Ag2CO3-t-BuOH_H2O
1	90	S3-LC-15-CP8-Pd(OAc)2-K2HPO4-Ag2CO3-t-BuOH_H2O
1	84	S3-LC-15-CP42-Pd(OAc)2-K2HPO4-Ag2CO3-t-BuOH_H2O
1	84	S3-LC-18-CP3-Pd(OAc)2-K2HPO4-Ag2CO3-t-BuOH_H2O
1	64	S3-LC-7-CP3-Pd(OAc)2-K2HPO4-Ag2CO3-t-BuOH_H2O
1	72	S3-LC-15-CP3-Pd(OAc)2-K2HPO4-Ag2CO3-THF_H2O
1	86	S3-LC-15-CP18-Pd(OAc)2-K2HPO4-Ag2CO3-t-BuOH_H2O
1	50	S3-LD-1-CP3-Pd(OAc)2-K2HPO4-Ag2CO3-t-BuOH_H2O
1	84	S3-LC-15-CP14-Pd(OAc)2-K2HPO4-Ag2CO3-t-BuOH_H2O
1	74	S3-LC-9-CP3-Pd(OAc)2-K2HPO4-Ag2CO3-t-BuOH_H2O
1	70	S3-LC-20-CP3-Pd(OAc)2-K2HPO4-Ag2CO3-t-BuOH_H2O
1	70	S3-LC-17-CP3-Pd(OAc)2-K2HPO4-Ag2CO3-t-BuOH_H2O
1	50	S3-LA-2-CP3-Pd(OAc)2-K2HPO4-Ag2CO3-t-BuOH_H2O
1	62	S3-LC-10-CP3-Pd(OAc)2-K2HPO4-Ag2CO3-t-BuOH_H2O
2	76	S5-LB-27-CP4-Pd(OAc)2-Na2CO3-Ag2CO3-t-AmylOH_H2O
2	43	S5-LB-3-CP4-Pd(OAc)2-Na2CO3-Ag2CO3-t-AmylOH_H2O
2	80	S5-LB-13-CP4-Pd(OAc)2-Na2CO3-Ag2CO3-t-AmylOH_H2O
2	79	S5-LB-19-CP4-Pd(OAc)2-Na2CO3-Ag2CO3-t-AmylOH_H2O
2	81	S5-LB-26-CP4-Pd(OAc)2-Na2CO3-Ag2CO3-t-AmylOH_H2O
2	82	S5-LB-22-CP4-Pd(OAc)2-Na2CO3-Ag2CO3-t-AmylOH_H2O
2	84	S5-LB-16-CP4-Pd(OAc)2-Na2CO3-Ag2CO3-t-AmylOH_H2O
2	74	S5-LB-8-CP4-Pd(OAc)2-Na2CO3-Ag2CO3-t-AmylOH_H2O
2	82	S5-LB-11-CP4-Pd(OAc)2-Na2CO3-Ag2CO3-t-AmylOH_H2O
2	80	S5-LB-12-CP4-Pd(OAc)2-Na2CO3-Ag2CO3-t-AmylOH_H2O
2	83	S5-LB-14-CP4-Pd(OAc)2-Na2CO3-Ag2CO3-t-AmylOH_H2O
2	24	S5-LA-22-CP4-Pd(OAc)2-Na2CO3-Ag2CO3-t-AmylOH_H2O
2	60	S5-LB-2-CP4-Pd(OAc)2-Na2CO3-Ag2CO3-t-AmylOH_H2O
2	31	S5-LA-3-CP4-Pd(OAc)2-Na2CO3-Ag2CO3-t-AmylOH_H2O
2	34	S5-LA-23-CP4-Pd(OAc)2-Na2CO3-Ag2CO3-t-AmylOH_H2O
2	78	S5-LB-24-CP4-Pd(OAc)2-Na2CO3-Ag2CO3-t-AmylOH_H2O
2	78	S5-LB-4-CP4-Pd(OAc)2-Na2CO3-Ag2CO3-t-AmylOH_H2O
2	81	S5-LB-17-CP4-Pd(OAc)2-Na2CO3-Ag2CO3-t-AmylOH_H2O
2	79	S5-LB-5-CP4-Pd(OAc)2-Na2CO3-Ag2CO3-t-AmylOH_H2O
2	75	S5-LB-28-CP4-Pd(OAc)2-Na2CO3-Ag2CO3-t-AmylOH_H2O
2	76	S5-LB-21-CP4-Pd(OAc)2-Na2CO3-Ag2CO3-t-AmylOH_H2O
2	68	S5-LB-10-CP4-Pd(OAc)2-Na2CO3-Ag2CO3-t-AmylOH_H2O

2	78	S5-LB-20-CP4-Pd(OAc)2-Na2CO3-Ag2CO3-t-AmylOH_H2O
2	29	S5-LA-25-CP4-Pd(OAc)2-Na2CO3-Ag2CO3-t-AmylOH_H2O
2	79	S5-LB-1-CP4-Pd(OAc)2-Na2CO3-Ag2CO3-t-AmylOH_H2O
3	56	S2-LC-11-CP1-Pd(OAc)2-Na2CO3-Ag2CO3-HFIP
3	90	S2-LC-1-CP40-Pd(OAc)2-Na2CO3-Ag2CO3-HFIP
3	92	S2-LC-8-CP1-Pd(OAc)2-Na2CO3-Ag2CO3-HFIP
3	82	S2-LC-16-CP1-Pd(OAc)2-Na2CO3-Ag2CO3-HFIP
3	80	S2-LC-1-CP25-Pd(OAc)2-Na2CO3-Ag2CO3-HFIP
3	92	S2-LC-1-CP33-Pd(OAc)2-Na2CO3-Ag2CO3-HFIP
3	94	S2-LC-1-CP26-Pd(OAc)2-Na2CO3-Ag2CO3-HFIP
3	68	S2-LA-2-CP1-Pd(OAc)2-Na2CO3-Ag2CO3-HFIP
3	92	S2-LC-1-CP35-Pd(OAc)2-Na2CO3-Ag2CO3-HFIP
3	90	S2-LC-2-CP1-Pd(OAc)2-Na2CO3-Ag2CO3-HFIP
3	92	S2-LC-14-CP1-Pd(OAc)2-Na2CO3-Ag2CO3-HFIP
3	92	S2-LC-1-CP37-Pd(OAc)2-Na2CO3-Ag2CO3-HFIP
3	82	S2-LC-12-CP1-Pd(OAc)2-Na2CO3-Ag2CO3-HFIP
3	92	S2-LC-1-CP29-Pd(OAc)2-Na2CO3-Ag2CO3-HFIP
3	92	S2-LC-1-CP24-Pd(OAc)2-Na2CO3-Ag2CO3-HFIP
3	90	S2-LC-17-CP1-Pd(OAc)2-Na2CO3-Ag2CO3-HFIP
3	90	S2-LC-1-CP43-Pd(OAc)2-Na2CO3-Ag2CO3-HFIP
3	88	S2-LC-1-CP30-Pd(OAc)2-Na2CO3-Ag2CO3-HFIP
3	92	S2-LC-1-CP31-Pd(OAc)2-Na2CO3-Ag2CO3-HFIP
3	12	S2-LC-4-CP1-Pd(OAc)2-Na2CO3-Ag2CO3-HFIP
3	90	S2-LC-15-CP1-Pd(OAc)2-Na2CO3-Ag2CO3-HFIP
3	92	S2-LC-1-CP28-Pd(OAc)2-Na2CO3-Ag2CO3-HFIP
3	96	S2-LC-1-CP41-Pd(OAc)2-Na2CO3-Ag2CO3-HFIP
3	92	S2-LC-1-CP27-Pd(OAc)2-NaHCO3-AgOAc-HFIP
3	94	S2-LC-1-CP1-Pd(OAc)2-Na2CO3-Ag2CO3-HFIP
3	92	S2-LC-1-CP44-Pd(OAc)2-Na2CO3-Ag2CO3-HFIP
3	86	S2-LC-1-CP34-Pd(OAc)2-NaHCO3-AgOAc-HFIP
3	88	S2-LC-9-CP1-Pd(OAc)2-Na2CO3-Ag2CO3-HFIP
4	88	S2-LA-19-CP3-Pd(MeCN)4(BF4)2-K2HPO4-Ag2CO3-t-BuOH_H2O
4	96	S2-LA-13-CP50-[Pd(allyl)Cl]2-K2HPO4-Ag2CO3-t-BuOH_H2O
4	88	S2-LA-19-CP3-Pd(OAc)2-Na2CO3-Ag2CO3-t-BuOH_H2O
4	94	S2-LA-13-CP46-[Pd(allyl)Cl]2-K2HPO4-Ag2CO3-t-BuOH_H2O
4	96	S2-LA-19-CP15-Pd(OAc)2-K2HPO4-Ag2CO3-t-BuOH_H2O
4	96	S2-LA-13-CP3-Pd(OAc)2-K2HPO4-Ag2CO3-t-BuOH_H2O
4	86	S2-LA-12-CP3-Pd(OAc)2-K2HPO4-Ag2CO3-t-BuOH_H2O
4	98	S2-LA-19-CP21-Pd(OAc)2-K2HPO4-Ag2CO3-t-BuOH_H2O
4	88	S2-LA-19-CP3-Pd(OAc)2-KH2PO4-Ag2CO3-t-BuOH_H2O
4	94	S2-LA-8-CP3-Pd(OAc)2-K2HPO4-Ag2CO3-t-BuOH_H2O
4	96	S2-LA-13-CP45-[Pd(allyl)Cl]2-K2HPO4-Ag2CO3-t-BuOH_H2O
4	94	S2-LA-2-CP3-Pd(OAc)2-K2HPO4-Ag2CO3-t-BuOH_H2O
4	90	S2-LA-19-CP20-Pd(OAc)2-K2HPO4-Ag2CO3-t-BuOH_H2O

4	92	S2-LA-17-CP3-Pd(OAc)2-K2HPO4-Ag2CO3-t-BuOH_H2O
4	94	S2-LA-19-CP16-Pd(OAc)2-K2HPO4-Ag2CO3-t-BuOH_H2O
4	88	S2-LA-19-CP3-Pd(PhCN)2(Cl)2-K2HPO4-Ag2CO3-t-BuOH_H2O
4	90	S2-LA-6-CP3-Pd(OAc)2-K2HPO4-Ag2CO3-t-BuOH_H2O
4	82	S2-LA-19-CP3-Pd(PPh3)2(Cl)2-K2HPO4-Ag2CO3-t-BuOH_H2O
4	96	S2-LA-19-CP4-Pd(OAc)2-K2HPO4-Ag2CO3-t-BuOH_H2O
4	92	S2-LC-1-CP3-Pd(OAc)2-K2HPO4-Ag2CO3-t-BuOH_H2O
4	94	S2-LA-19-CP7-Pd(OAc)2-K2HPO4-Ag2CO3-t-BuOH_H2O
4	92	S2-LA-19-CP3-Pd(OAc)2-K2HPO4-AgOAc-t-BuOH_H2O
4	78	S2-LA-19-CP3-Pd(OAc)2-K2HPO4-Ag2CO3-HFIP_H2O
4	90	S2-LA-21-CP3-Pd(OAc)2-K2HPO4-Ag2CO3-t-BuOH_H2O
4	86	S2-LA-19-CP3-Pd(MeCN)2(Cl)2-K2HPO4-Ag2CO3-t-BuOH_H2O
4	88	S2-LA-4-CP3-Pd(OAc)2-K2HPO4-Ag2CO3-t-BuOH_H2O
4	92	S2-LA-1-CP3-Pd(OAc)2-K2HPO4-Ag2CO3-t-BuOH_H2O
4	84	S2-LA-19-CP19-Pd(OAc)2-K2HPO4-Ag2CO3-t-BuOH_H2O
4	88	S2-LA-19-CP3-Pd(OAc)2-KOAc-Ag2CO3-t-BuOH_H2O
4	98	S2-LA-19-CP5-Pd(OAc)2-K2HPO4-Ag2CO3-t-BuOH_H2O
4	80	S2-LA-3-CP3-Pd(OAc)2-K2HPO4-Ag2CO3-t-BuOH_H2O
4	84	S2-LA-18-CP3-Pd(OAc)2-K2HPO4-Ag2CO3-t-BuOH_H2O
4	94	S2-LA-10-CP3-Pd(OAc)2-K2HPO4-Ag2CO3-t-BuOH_H2O
4	98	S2-LA-19-CP23-Pd(OAc)2-K2HPO4-Ag2CO3-t-BuOH_H2O
4	90	S2-LA-5-CP3-Pd(OAc)2-K2HPO4-Ag2CO3-t-BuOH_H2O
4	94	S2-LA-19-CP18-Pd(OAc)2-K2HPO4-Ag2CO3-t-BuOH_H2O
4	94	S2-LA-20-CP3-Pd(OAc)2-K2HPO4-Ag2CO3-t-BuOH_H2O
4	90	S2-LA-11-CP3-Pd(OAc)2-K2HPO4-Ag2CO3-t-BuOH_H2O
4	90	S2-LA-13-CP49-[Pd(allyl)Cl]2-K2HPO4-Ag2CO3-t-BuOH_H2O
4	94	S2-LA-7-CP3-Pd(OAc)2-K2HPO4-Ag2CO3-t-BuOH_H2O
4	86	S2-LA-19-CP3-Pd(OAc)2-K2HPO4-Ag2O-t-BuOH_H2O
4	88	S2-LA-19-CP3-Pd(OAc)2-K2HPO4-Ag2CO3-t-AmylOH_H2O
5	88	S5-LB-18-CP4-Pd(OAc)2-Na2CO3-Ag2CO3-THF_H2O
5	89	S5-LB-18-CP7-Pd(OAc)2-Na2CO3-Ag2CO3-t-AmylOH_H2O
5	90	S5-LB-18-CP14-Pd(OAc)2-Na2CO3-Ag2CO3-t-AmylOH_H2O
5	45	S5-LA-27-CP4-Pd(OAc)2-Na2CO3-Ag2CO3-t-AmylOH_H2O
5	89	S5-LB-18-CP3-Pd(OAc)2-Na2CO3-Ag2CO3-t-AmylOH_H2O
5	81	S5-LB-18-CP4-Pd(OPiv)2-Na2CO3-Ag2CO3-t-AmylOH_H2O
5	77	S5-LB-18-CP4-Pd(OAc)2-Na2CO3-Ag2CO3-i-PrOH_H2O
5	90	S5-LB-18-CP8-Pd(OAc)2-Na2CO3-Ag2CO3-t-AmylOH_H2O
5	93	S5-LB-18-CP13-Pd(OAc)2-Na2CO3-Ag2CO3-t-AmylOH_H2O
5	85	S1-LB-18-CP4-Pd(OAc)2-Na2CO3-Ag2CO3-t-AmylOH_H2O
5	80	S5-LB-18-CP4-Pd(OAc)2-Li2CO3-Ag2CO3-t-AmylOH_H2O
5	89	S5-LB-18-CP10-Pd(OAc)2-Na2CO3-Ag2CO3-t-AmylOH_H2O
5	92	S5-LB-18-CP5-Pd(OAc)2-Na2CO3-Ag2CO3-t-AmylOH_H2O
5	88	S5-LB-18-CP4-Pd(OAc)2-Na2HPO4-Ag2CO3-t-AmylOH_H2O
5	88	S5-LB-18-CP4-Pd(OAc)2-Na2CO3-Ag2CO3-t-AmylOH_H2O

5	88	S5-LB-18-CP4-Pd(OAc)2-KHCO3-Ag2CO3-t-AmylOH_H2O
5	69	S5-LB-18-CP4-Pd(OAc)2-KOAc-Ag2CO3-t-AmylOH_H2O
6	82	S4-LD-2-CP2-Pd(OAc)2-NaH2PO4-Ag2CO3-C6F6
6	92	S4-LD-2-CP2-Pd(OAc)2-NaH2PO4-Ag2CO3-t-AmylOH
6	88	S4-LD-2-CP2-Pd(OAc)2-NaH2PO4-Ag2CO3-DCE
6	88	S4-LD-2-CP2-Pd(OAc)2-NaH2PO4-Ag2CO3-THF
6	76	S4-LD-2-CP2-Pd(OAc)2-NaH2PO4-Ag2CO3-Et2O
6	80	S4-LD-2-CP2-Pd(OAc)2-NaH2PO4-Ag2CO3-CCl4
6	66	S4-LD-2-CP2-Pd(OAc)2-NaH2PO4-Ag2CO3-MeCN
6	89	S4-LD-2-CP2-Pd(OAc)2-NaH2PO4-Ag2CO3-DCM

6. Hyper-parameter tuning for the target task generator

We have evaluated the model training by measuring three key aspects; (1) validity - the percentage of generated SMILES strings that can be converted back into molecular structures, (2) uniqueness - the percentage of distinct SMILES strings without duplicates, and (3) novelty - the percentage of generated SMILES strings not found in the training data. High validity shows the model can generate chemically meaningful SMILES strings; high uniqueness means the generation is diverse, and high novelty indicates the model can create new molecules from scratch. In the context of the ULMFiT-based text generation, performance indicators such as train loss, validation loss, accuracy, and error rate are considered. Train loss indicates how well the model fits the training data, with lower values being better. Validation loss measures how well the model generalizes to unseen data. Accuracy reflects the proportion of correctly predicted tokens in the validation set, where higher values are better, while error rate represents the proportion of incorrectly predicted tokens, with lower values being desirable. These metrics provide insights into the model performance, helping to identify issues such as overfitting (high train accuracy but low valid accuracy) or underfitting (low train accuracy).

Table S14. Performance Obtained by Varying Number of Augmented SMILES.[i] Shown in Bold Font is the Optimal Model

augmentation	train loss	valid loss	accuracy	error rate	validity	uniqueness	novelty

0	0.675864	0.964036	0.728125	0.271875	0	0	0
25	0.225234	0.490671	0.846763	0.153237	0.96	0.96	0.96
50	0.227066	0.360535	0.869922	0.130078	0.98	0.97	0.96
100	0.226238	0.318261	0.876079	0.123921	0.99	0.99	0.99
**200**	**0.224793**	**0.265236**	**0.885532**	**0.114468**	**1**	**1**	**0.99**

[i]augmented SMILES = 100, batch size = 128, dropout ratio = 0.0, learning rate = [1e-1, 1e-2, 1e-3], epoch = [5,6,6]

Table S15. Performance Obtained by Varying the Dropout Rate.[i] Shown in Bold Font is the Optimal Model

dropout rate	train loss	valid loss	accuracy	error rate	validity	uniqueness	novelty
0.0	0.224793	0.265236	0.885532	0.114468	1	1	0.99
0.1	0.229315	0.264232	0.886607	0.113393	0.99	0.98	0.97
0.2	0.231952	0.26415	0.887054	0.112946	1	0.99	0.99
0.3	0.231593	0.263500	0.887094	0.112906	1	0.99	0.99
0.4	0.233399	0.263111	0.887378	0.112622	0.99	0.99	0.99
0.5	0.23423	0.263813	0.887297	0.112703	1	1	1
0.6	0.23289	0.26373	0.8874	0.112600	0.99	0.99	0.99
0.7	0.234257	0.263637	0.888129	0.111871	0.97	0.97	0.97
**0.8**	**0.23761**	**0.26403**	**0.88823**	**0.11177**	**0.99**	**0.99**	**0.99**
0.9	0.239185	0.264236	0.887886	0.112114	1	1	0.99

[i]augmented SMILES = 200, batch size = 128, learning rate = [1e-1, 1e-2, 1e-3], epoch = [5,6,6]

Table S16. Performance Obtained by Varying Number of Epochs.[i] Shown in Bold Font is the Optimal Model

epoch	train loss	valid loss	accuracy	error rate	validity	uniqueness	novelty
[4,4,4]	0.24251	0.26811	0.88748	0.11252	0.99	0.99	0.97
[4,5,5]	0.23857	0.2654	0.88785	0.11216	1	1	1
[4,5,6]	0.23813	0.26321	0.88766	0.11234	0.98	0.98	0.98
[5,5,5]	0.23888	0.26699	0.88683	0.11317	1	1	0.98
**[5,6,6]**	**0.23761**	**0.26403**	**0.88823**	**0.11177**	**0.99**	**0.99**	**0.99**
[5,6,7]	0.23794	0.26231	0.8877	0.1123	0.99	0.99	0.99
[6,6,6]	0.23766	0.26636	0.88714	0.11287	0.98	0.97	0.97
[6,7,7]	0.23573	0.26513	0.8864	0.1136	0.99	0.99	0.98

[i]augmented SMILES = 200, batch size = 128, learning rate = [1e-1, 1e-2, 1e-3], dropout ratio = 0.8

Table S17. Performance Obtained by Varying Learning Rate.[i] Shown in Bold Font is the Optimal Model

learning rate	train loss	valid loss	accuracy	error rate	validity	uniqueness	novelty
**[1e-1, 1e-2, 1e-3]**	**0.23761**	**0.26403**	**0.88823**	**0.11177**	**0.99**	**0.99**	**0.99**
[1e-2, 1e-2, 1e-3]	0.25311	0.27486	0.88705	0.11295	1	1	1
[1e-2, 1e-2, 1e-4]	0.2705	0.28852	0.8849	0.1151	0.93	0.93	0.93
[1e-2, 1e-2, 1e-5]	0.244418	0.266653	0.887013	0.112987	0.9	0.9	0.9
[3e-2,slice(5e-3/(2.6^4),5e-3),slice(5e-4/(2.6^4),5e-4)]	0.26393	0.28042	0.88618	0.11382	0.98	0.97	0.97
[3e-1,slice(5e-4/(2.6^4),5e-4),slice(5e-5/(2.6^4),5e-5)]	0.30647	0.31227	0.87778	0.12222	0.95	0.95	0.95

Table S18. The Effect of Different Sampling Temperatures on Validity, Uniqueness, and Novelty of the Generated Molecules

Validity									
runs	0.2	0.6	**0.8**	1.0	1.2	1.4	1.6	1.8	2.0
1	100	100	**99.8**	99.2	96.8	86.4	73	53.4	34.4
2	100	100	**99.6**	98.8	95.2	86.4	76.2	57.4	34.4
3	100	100	**99.8**	98.4	95	86.6	69.8	51.2	33.6
Uniqueness									
	0.2	0.6	**0.8**	1.0	1.2	1.4	1.6	1.8	2.0
1	30.8	96	**98.4**	98.8	95.8	85.6	72	53.2	34.2
2	34	95.8	**97.6**	97.8	93.6	84.4	76	57.2	34.2
3	33.6	95.8	**97.6**	96	94.4	86.2	68.8	51	32.8
Novelty									
	0.2	0.6	**0.8**	1.0	1.2	1.4	1.6	1.8	2.0
1	30.8	95.6	**98**	98.6	95.4	85.6	72	52.8	34.2
2	34	95.4	**97.2**	97.6	93	83.8	75.8	57.2	34.2
3	33.6	95.6	**97.4**	95.8	94	86	68.4	51	32.4

7. Details of the generated chiral ligands

We have used a temperature-based sampling procedure intending to generate 500 SMILES strings from the fine-tuned model, which exhibited a validity of 98%, uniqueness of 98%, and novelty of 97.8%. This resulted in 489 new SMILES strings. However, some of the generated ligands might not meet specific criteria, such as lacking a chiral center, having not more than 2 chiral centers, or missing the desired functional groups such as the –NHCO fragment for binding with the transition metal to serve as a catalyst. Others might not even possess the key backbone that renders the molecule with a chelate binding to the transition metal, as shown in Fig. S7. To address this, we applied various filters that eventually offered 73 new chiral ligands that meet all the required criteria mandated by the reaction of interest.

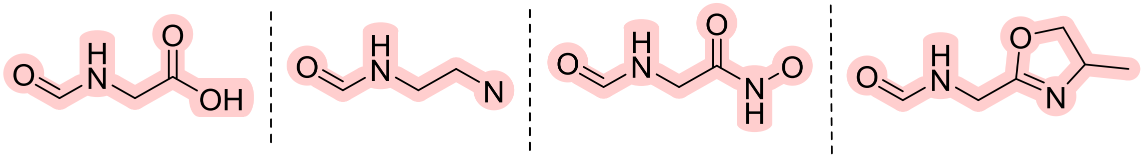

Fig. S7. Important backbone required for a generated to act as a chiral ligand in our reaction of interest

The set of 73 generated novel chiral ligands with their chemical structure is shown in the following Table S19.

Table S19. Identities and Notations of the Generated Chiral Ligands

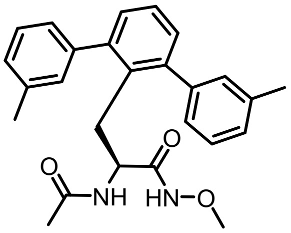	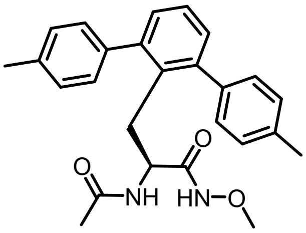	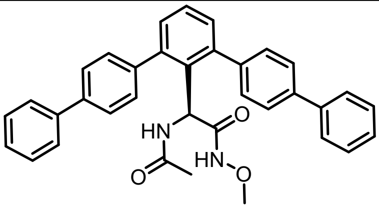
**L1**	**L2**	**L3**

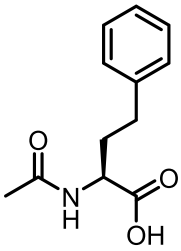	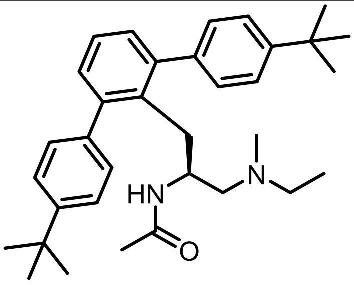	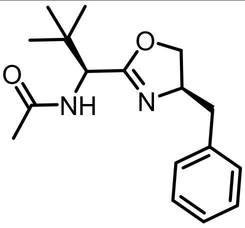
**L4**	**L5**	**L6**
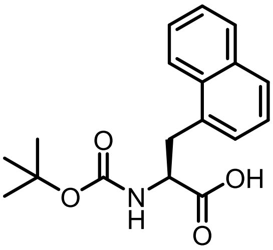	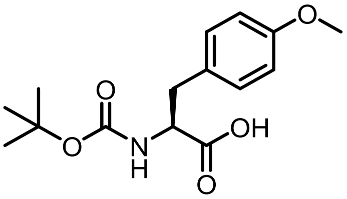	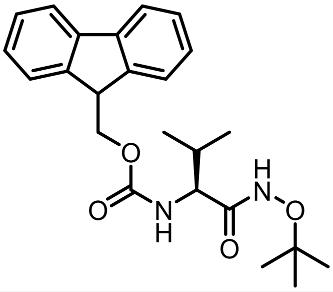
**L7**	**L8**	**L9**
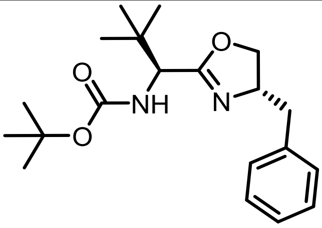	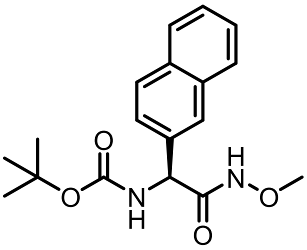	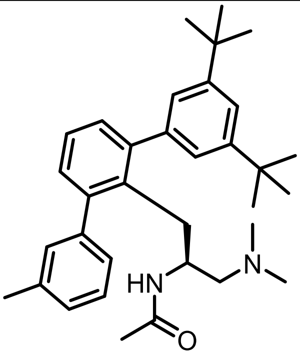
**L10**	**L11**	**L12**
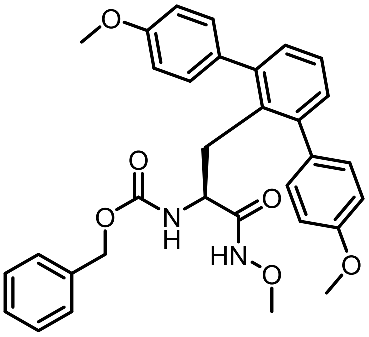	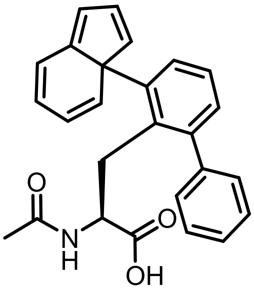	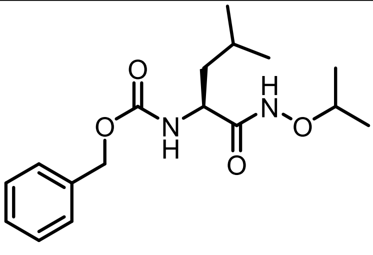
**L13**	**L14**	**L15**
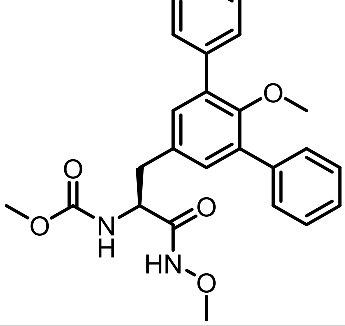	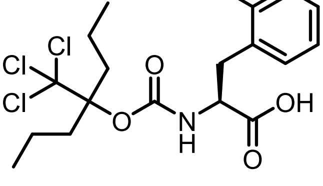	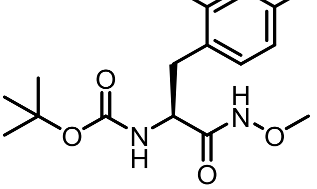
**L16**	**L17**	**L18**

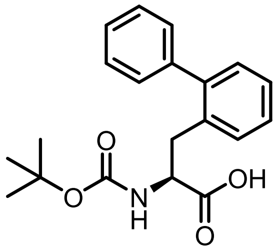	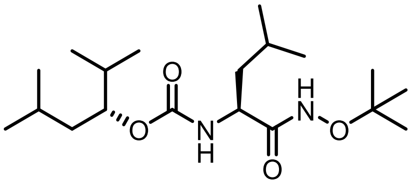	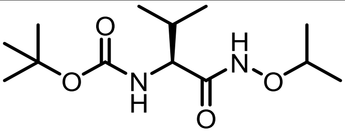
**L19**	**L20**	**L21**
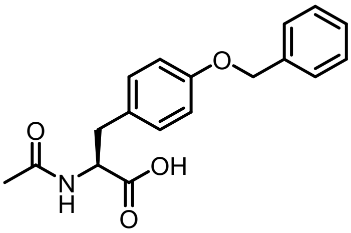	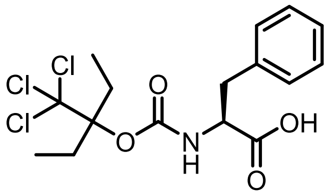	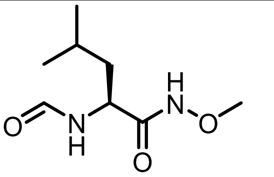
**L22**	**L23**	**L24**
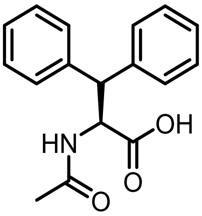	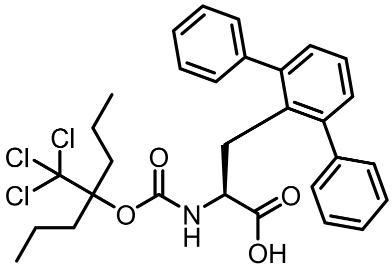	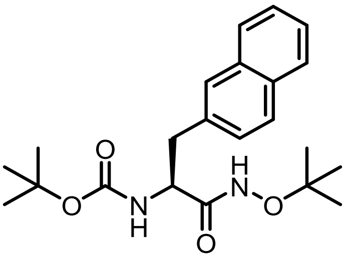
**L25**	**L26**	**L27**
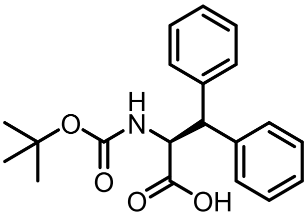	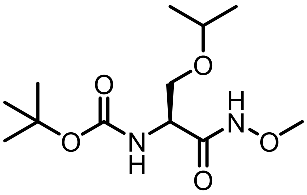	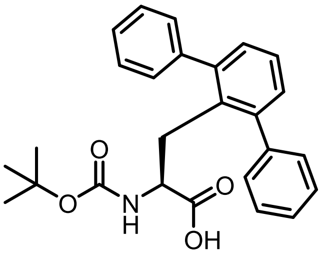
**L28**	**L29**	**L30**
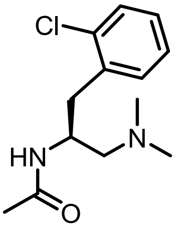	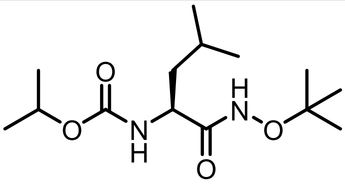	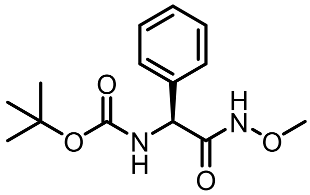
**L31**	**L32**	**L33**
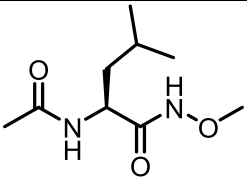	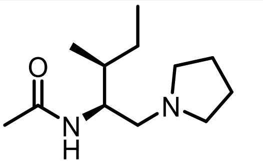	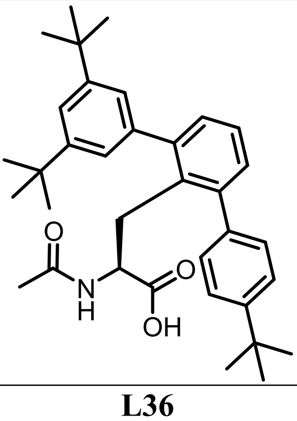
**L34**	**L35**	**L36**

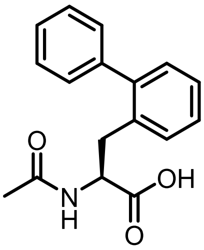	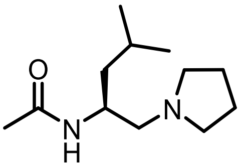	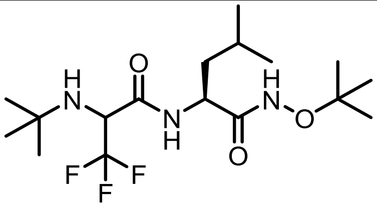
**L37**	**L38**	**L39**
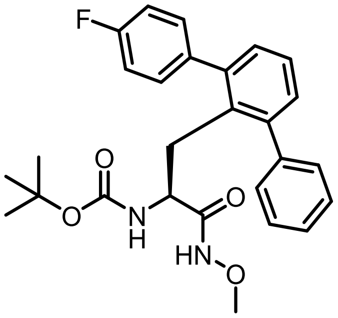	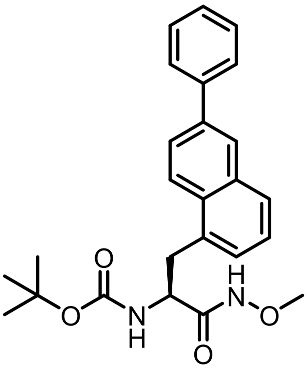	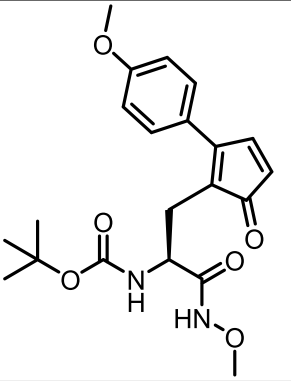
**L40**	**L41**	**L42**
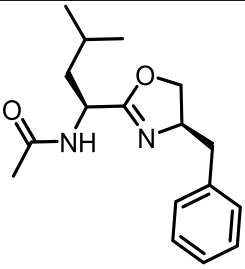	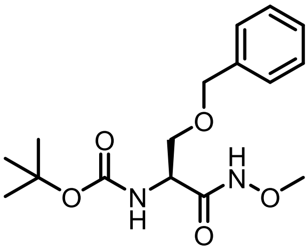	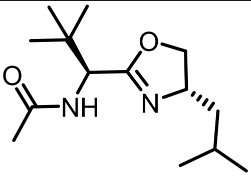
**L43**	**L44**	**L45**
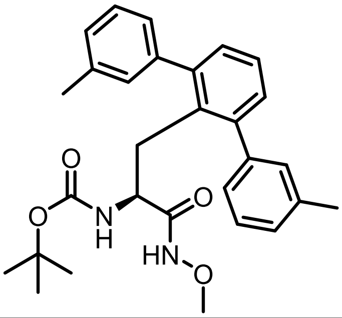	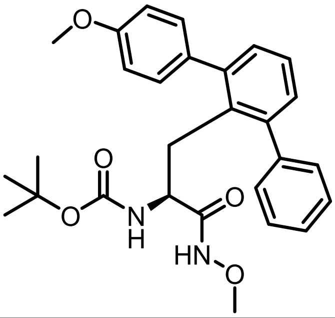	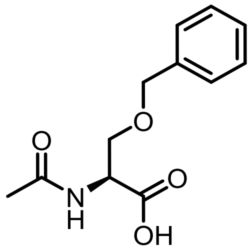
**L46**	**L47**	**L48**
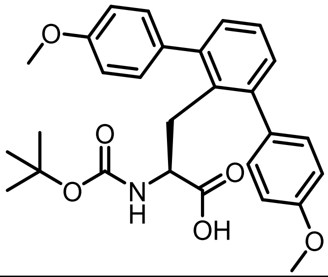	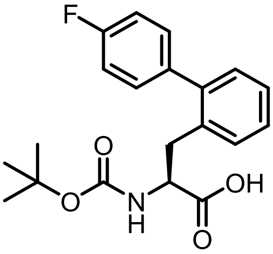	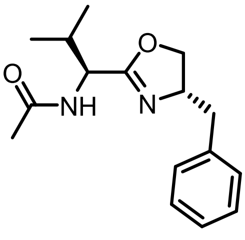
**L49**	**L50**	**L51**

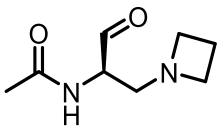	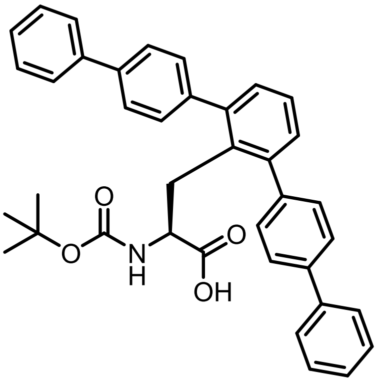	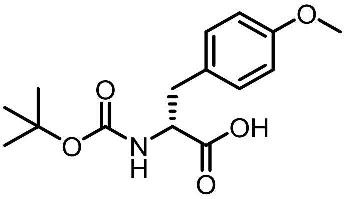
**L52**	**L53**	**L54**
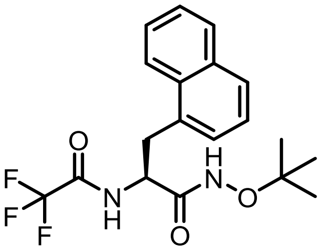	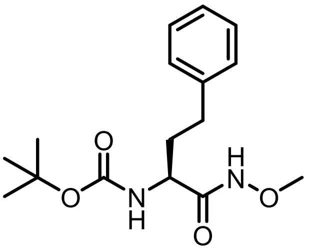	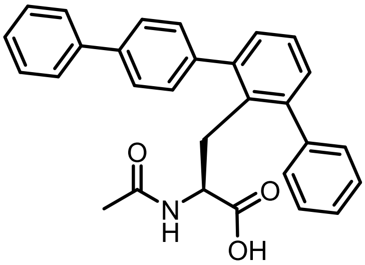
**L55**	**L56**	**L57**
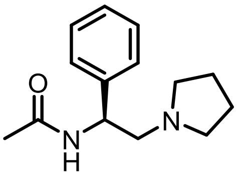	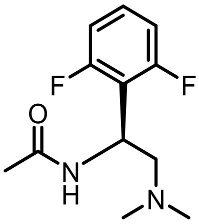	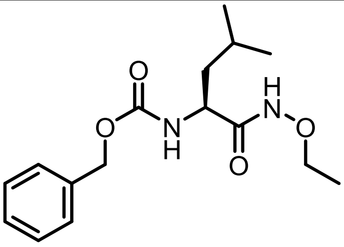
**L58**	**L59**	**L60**
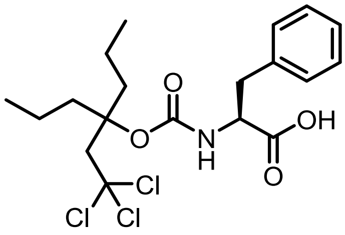	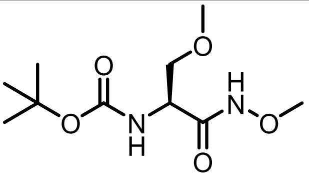	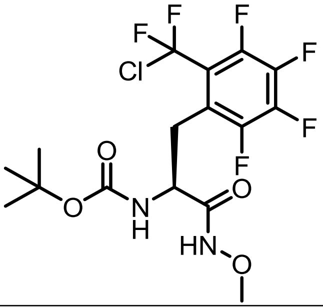
**L61**	**L62**	**L63**
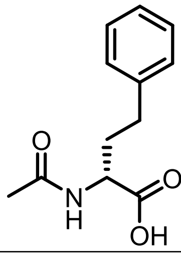	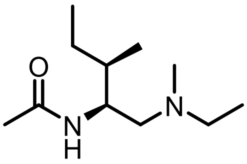	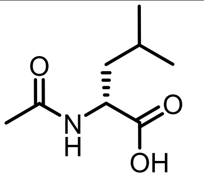
**L64**	**L65**	**L66**

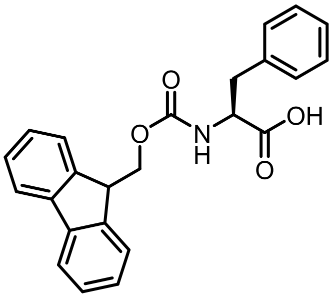	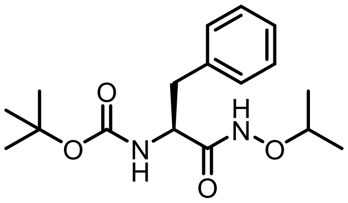	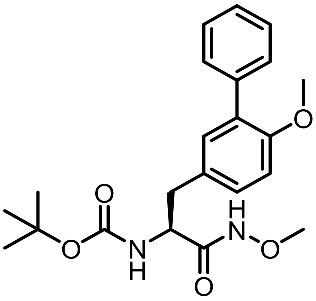
**L67**	**L68**	**L69**
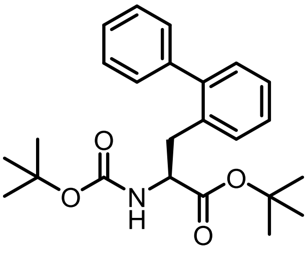	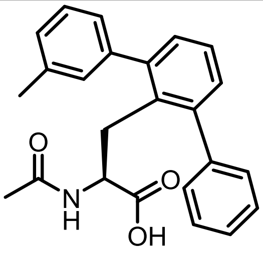	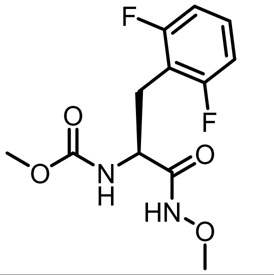
**L70**	**L71**	**L72**
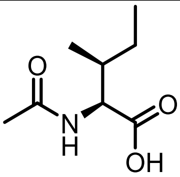		
**L73**		

8. Comparison between the generated and experimental ligands

To compare the chemical space generated by the fine-tuned generator with experimental ligands, we used a TMAP plot enabled by the Faerun visualization library.[13] The TMAP algorithm is known its ability to generate a two-dimensional dendrogram-like representation that preserves local and global relationships, focusing on the local. In this technique, MinHashing permutation is set as 512 in order to estimate the similarity between data sets. Then, the MHFP (MinHash Fingerprint) encoder is initialized with these permutations to generate fingerprint vectors from the SMILES strings that capture chemical structure similarities. An LSH (Locality-Sensitive Hashing) forest is set up using the same permutations to facilitate efficient similarity searches and rapid nearest-neighbor identification. The process proceeds to create a Faerun plot (shown in Fig. 3 in the manuscript), adding scatter points colored by label values and a tree structure connecting the related points. The resulting plot revealed several key aspects; (a) four distinct leaf-like

structures globally corresponding to different ligand classes, (b) chemically or structurally similar molecules from both the generated and experimental sets are positioned close to each other, and (c) the alignment of the generated ligands near experimental ones suggests that the fine-tuned generator explores the nearby chemical space. Thus, this plot helps in identifying clusters and patterns and enables the recognition of areas where generated ligands align with, or differ, from the experimentally known ones.

We have utilized the encoder output obtained from the fine-tuned generator to visualize them using the t-SNE plots as well as using the corresponding 2048-bit Morgan fingerprint vector. This is done to compare the most informative features of the generated and experimentally known chiral ligands. Subsequent 3D plotting of the top three principal components revealed neighboring exploration (Shown in Fig. S8).

(a) **(b)**

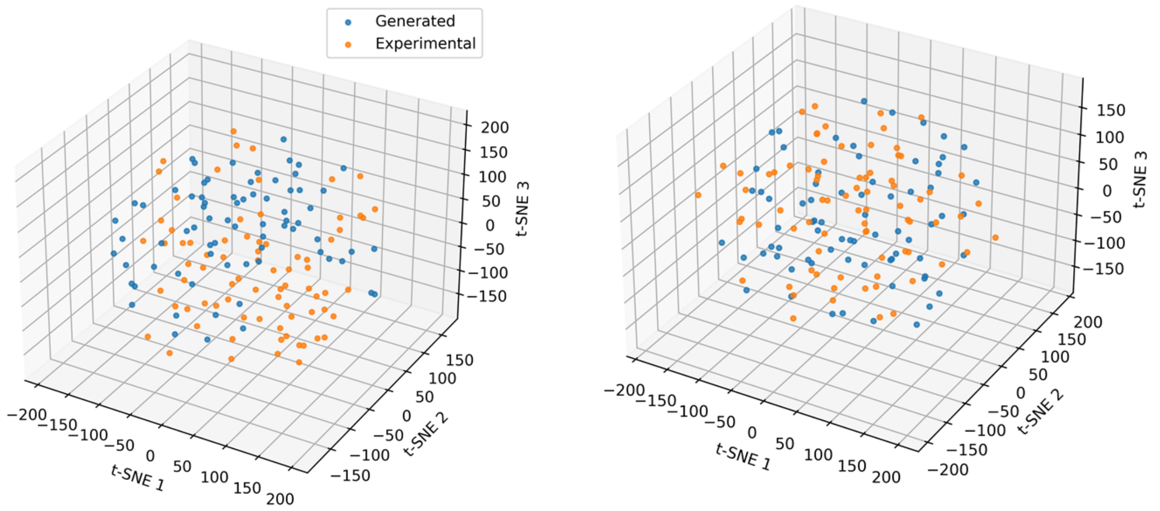

Fig. S8. The training and generated chemical space occupied by the chiral ligands depicted using the t-SNE plots obtained from the (a) encoder output, and (b) fingerprint representation technique. The training set is represented by orange color dots, while the generated set is shown using the blue dots.

After comparing the generated and the experimentally reported ligands by using the TMAP and t-SNE methods, we conducted a more direct assessment of how the generated

ligands resemble the real ones in terms of various chemical properties. Properties, such as LogP (hydrophobic/hydrophilic nature), NP-likeness score (natural product likelihood), QED (drug-likeness), hydrogen bond acceptor count (molecular interactions), and polar surface area (solubility), are found to exhibit minimal variations. The generated chemical space exhibits slight deviations in physicochemical properties compared to the experimental set, indicating a localized exploration of chemical space (Fig. S9).

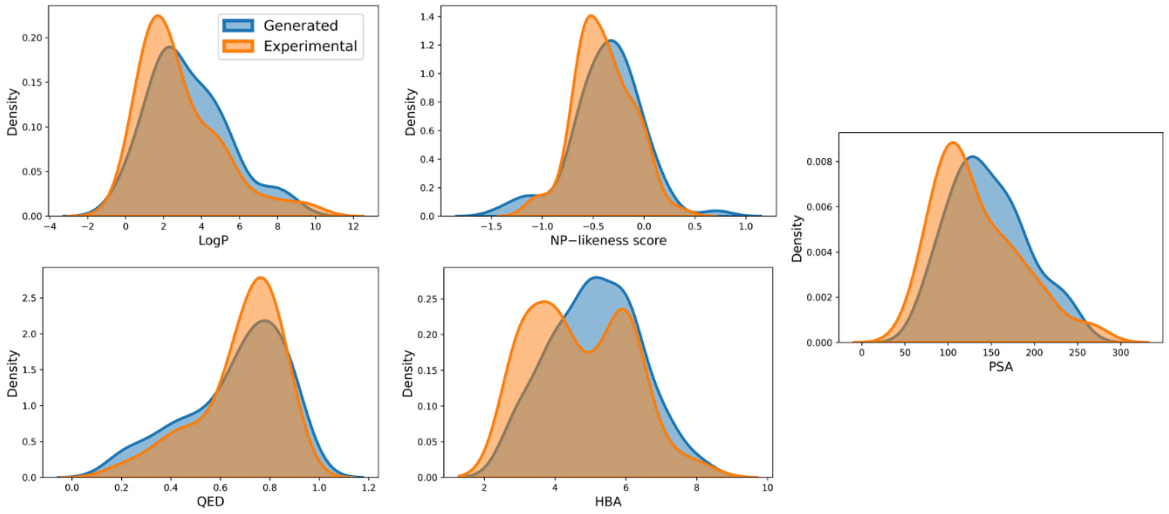

Fig. S9. Comparison of various physicochemical properties between the training and generated ligands. The key properties considered are LogP (hydrophobic/hydrophilic nature), NP-likeness score, QED, hydrogen bond acceptor count, and polar surface area (solubility).

9. Reactions space using the generated ligands

Using a fine-tuned TL-based generator, we generated 73 new chiral ligands and utilized them to design novel reactions. From a pool of 220 experimentally reported reactions, we have selected one reaction and systematically substituted the original chiral ligand with each of the 73 newly generated ligands, resulting in 73 reaction variants of the original reaction. Given that there are 135 unique combinations of all reacting components (excluding the ligand) for the 220 reactions, this substitution process was repeated across all the reactions, leading to

9855 novel reactions, which could potentially be carried out. These reactions feature diverse combinations of cycloalkane substrates, coupling partners, newly generated chiral ligands, bases, and solvents, none of which have been experimentally validated.

We employed our EnP model to predict the %*ee*s of these newly generated reactions. The %*ee* predictions represent the average of the output values obtained from 30 different models. The distribution of the predicted %*ee* values for the 9855 reactions is shown using a heat map representation (Fig. 5c). A comprehensive detail of each reaction component and its corresponding %*ee* can be found in the CSV file available in the GitHub repository. A histogram depicting the %*ee* distribution, given in Fig. S10, highlights a substantial number of reactions in the high %*ee* range, mirroring the distribution as seen in the original experimental training set.

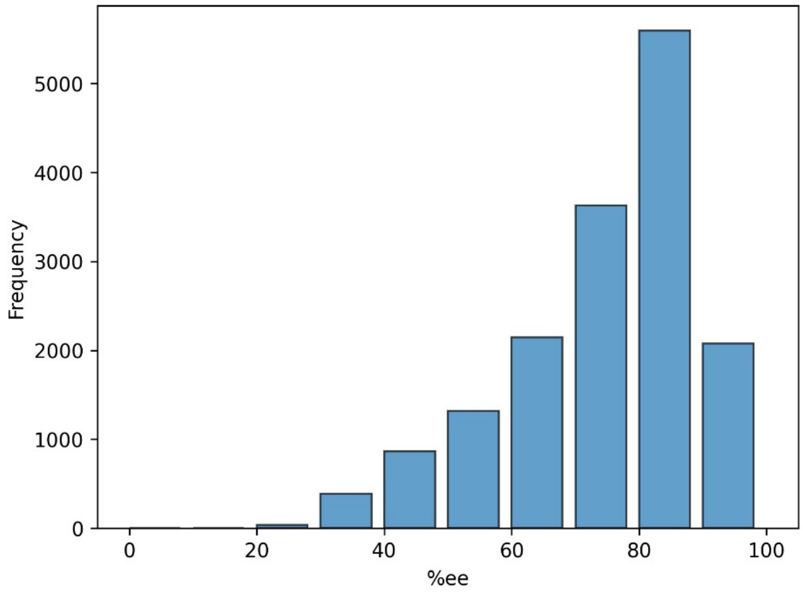

Fig. S10. Number of newly designed reactions obtained from the generated chiral ligands for different class intervals of the predicted %*ee*.

10. Performance comparison of different types of regressor

We compared the performance of various deep learning and tree-based models as baselines to evaluate how well the TL-based ensemble regressor performs in the *ee* prediction task. It was found that these regressors showed poorer performance compared to our EnP model. The details of these models and their performances are discussed below.

A deep neural network (DNN) consisting of multiple fully connected input layers, one or more hidden layers, and an output layer is developed. Key hyperparameters, such as the number of hidden layers, neurons per layer, learning rate, and dropout rate were found to significantly impact the model performance. We have used Optuna, a Python-based framework, to efficiently optimize these hyperparameters.[14] In this study, each reaction instance is represented by a comprehensive molecular fingerprint vector, created by concatenating several fingerprint types —Morgan, atom pair, RDKit, layered, topological torsion (TT), Avalon, and MACCS. This combined fingerprint vector serves as the input to the DNN, which is trained to predict the *ee* of the reaction.

Table S20. Performance of DNN Algorithm in terms of RMSE across Different Splits using Fingerprint Featurization Technique

split	train	validation	test	hyperparameters {n_layers, n_neurons, dropout_rate, learning_rate}
1	4.54	5.66	9.54	2,598,0.22,0.08298
2	4.01	5.32	7.82	2,131,0.14,0.02189
3	7.38	10.34	9.76	1,523,0.02,0.00171
4	8.73	8.25	7.56	4,264,0.14,0.02220
5	4.37	7.23	7.36	2,616,0.12,0.01896
6	9.61	11.26	12.13	5,255,0.17,0.00449
7	3.29	7.62	7.61	1,802,0.44,0.04972
8	5.71	7.05	8.83	3,321,0.42,0.03454
9	13.20	14.08	16.51	1,371,0.45,0.00156
10	4.83	5.25	10.32	3,591,0.12,0.00368
11	4.48	5.86	8.69	2,553,0.44,0.00467
12	4.62	5.70	12.32	1,867,0.43,0.00228
13	10.36	10.99	7.08	5,486,0.50,0.03069
14	3.63	7.98	7.88	1,184,0.21,0.00981

15	4.71	4.83	18.77	3,382,0.22,0.00535
16	4.44	5.83	8.33	2,517,0.31,0.00421
17	4.50	6.45	12.31	3,157,0.20,0.01523
18	7.68	6.59	10.17	4,834,0.25,0.00353
19	11.76	15.86	15.67	5,533,0.12,0.00309
20	8.57	6.13	11.10	4,982,0.32,0.00265
21	4.04	4.89	6.66	2,576,0.31,0.00664
22	12.00	10.65	11.68	5,559,0.40,0.00256
23	10.25	11.09	11.98	1,812,0.29,0.00098
24	3.81	9.29	5.79	1,950,0.47,0.06155
25	8.26	6.88	6.81	4,685,0.31,0.00373
26	4.72	7.50	10.29	1,747,0.50,0.00170
27	4.33	9.38	6.72	1,331,0.34,0.00347
28	6.49	10.83	7.98	3,683,0.25,0.04698
29	4.15	5.23	8.48	2,909,0.39,0.00138
30	5.88	5.74	9.80	3,102,0.26,0.01448
**avg.±s.d.**	6.48±2.82	7.99±2.77	9.87±3.0	

A Random Forest (RF) model, which comprises of an ensemble of decision trees, is developed to predict the *ee* of reactions using the same combined fingerprint vectors utilized in the DNN model. Key hyperparameters, including the number of trees (n_estimators), maximum tree depth, minimum samples per split, and maximum features considered for splitting, are the important parameters that can influence the performance of the RF model. We employed Optuna, a Python-based framework, to efficiently optimize these parameters.

Table S21. Performance of RF Algorithm in terms of RMSE across Different Splits using Fingerprint Featurization Technique

split	train	validation	test	parameters {n_estimators, max_depth, min_samples_split, min_samples_leaf, max_features}
1	5.00	10.56	8.27	53,7,5,1,auto
2	4.71	5.88	7.12	31,7,3,1,auto
3	4.94	15.31	8.98	53,6,2,1,auto
4	4.99	9.65	8.60	184,5,2,1,auto
5	3.68	8.02	7.60	113,27,4,1,auto
6	4.15	7.84	9.00	32,15,4,1,auto
7	3.97	6.21	7.78	82,11,2,1,auto
8	4.88	6.66	9.37	57,27,6,1,auto
9	4.23	11.88	7.14	141,32,3,1,sqrt

10	4.51	7.15	6.85	184,13,5,1,auto
11	3.95	5.70	7.63	161,23,3,1,auto
12	4.92	4.56	12.54	35,13,7,2,auto
13	4.27	11.05	6.67	91,7,3,1,auto
14	4.04	7.85	6.41	161,23,3,1,auto
15	5.02	5.47	9.96	83,7,6,1,auto
16	3.68	7.27	10.10	100,11,3,1,auto
17	7.69	11.37	8.34	28,5,5,5,auto
18	3.82	6.34	7.41	181,18,3,1,auto
19	4.17	11.04	9.11	121,8,2,1,auto
20	3.47	4.70	10.27	28,12,3,1,auto
21	4.52	5.48	6.59	199,19,5,1,auto
22	4.02	7.57	7.91	187,27,4,1,auto
23	5.32	8.65	6.91	187,5,3,1,auto
24	6.07	12.02	6.38	166,4,5,1,auto
25	5.14	7.86	6.59	22,7,6,1,auto
26	5.06	8.91	9.81	91,5,2,1,auto
27	4.11	7.23	7.00	199,12,4,1,auto
28	4.63	6.67	7.72	67,13,6,1,auto
29	4.53	7.10	7.76	23,7,2,1,sqrt
30	7.02	6.59	7.56	142,7,9,4,auto
**avg.±s.d.**	4.68±0.91	8.09±2.48	8.11±1.41	

We employed the AttentiveFP architecture with convolutional layers for atomic feature extraction, a readout layer for molecular embeddings, and a fully connected layer to predict %*ee* values.[15] We used Optuna for automated hyperparameter optimization, tuning both general hyperparameters (e.g., learning rate) and model-specific parameters (e.g., number of layers, graph feature size, and dropout rate). The search space included the number of layers for node embedding {1 to 5}, graph feature size {100 to 500}, dropout rate {1 to 5}, learning rate {10^{-5} to 10^{-1}}, and num timesteps for graph embedding {1 to 3}.

Table S22. Performance of AttentiveFP Algorithm in terms of RMSE across Different Splits

split	train	validation	test	hyperparameters {num_layers, graph_feat_size, dropout_rate, learning_rate, num_timesteps }
1	7.85	5.72	9.97	1,242,0.26,0.00960
2	10.16	6.87	10.86	1,264,0.19,0.00053
3	4.88	8.11	6.86	4,438,0.28,0.00115
4	6.08	14.30	9.57	2,224,0.12,0.00378
5	7.53	9.38	10.04	3,425,0.34,0.00061
6	7.02	8.89	9.66	1,253,0.05,0.00595

7	12.08	7.43	13.03	1,462,0.23,0.00015
8	4.05	10.40	9.97	3,290,0.10,0.00192
9	5.25	12.71	9.12	2,417,0.22,0.00129
10	9.36	7.59	8.30	1,139,0.34,0.00439
11	7.40	11.05	9.71	3,344,0.12,0.00412
12	5.89	7.51	13.53	2,379,0.32,0.00239
13	11.27	10.89	11.27	3,435,0.21,0.00667
14	4.71	7.90	7.69	4,317,0.30,0.00163
15	6.34	5.08	9.74	2,359,0.32,0.00257
16	4.87	7.68	10.05	3,499,0.08,0.00086
17	6.70	11.04	7.03	2,152,0.01,0.00422
18	6.99	7.01	8.35	2,349,0.16,0.00528
19	6.92	10.29	9.50	2,182,0.09,0.00525
20	6.60	6.54	8.76	2,338,0.07,0.00197
21	8.05	6.56	9.22	1,188,0.29,0.00892
22	5.76	12.70	8.50	2,410,0.37,0.00278
23	5.05	13.92	10.62	4,449,0.26,0.00108
24	5.62	11.74	8.12	3,314,0.02,0.00158
25	6.92	8.36	9.39	2,274,0.11,0.00219
26	8.48	11.30	12.81	2,449,0.26,0.00468
27	7.74	8.11	7.91	2,139,0.10,0.00894
28	7.80	9.02	8.81	3,233,0.12,0.00069
29	7.71	12.61	8.24	1,380,0.34,0.00480
30	5.73	6.64	11.61	2,318,0.29,0.00334
**avg.±s.d.**	7.03±1.86	9.25±2.47	9.61±1.62	

In our study, we selected the state-of-the-art T5Chem model as a baseline for predicting *ee*.[16] T5Chem is based on the "Text-to-Text Transfer Transformer" (T5) framework, originally designed for natural language processing. This model has demonstrated success in various chemical reaction prediction tasks, including reaction type classification, forward reaction prediction, retrosynthesis, and reaction yield prediction, using open-source datasets like USPTO. The model was first pre-trained on a large pool of PubChem molecules. For our %*ee* prediction task, we fine-tuned T5Chem using a hidden dimension of 256, an intermediate feed-forward layer of 2048, an initial learning rate of 5e-4, a weight decay of 0.01, trained over 50 epochs with a batch size of 64.

Table S23. Performance of T5Chem Algorithm in terms of RMSE across Different Splits

split	train	validation	test
1	6.58	5.50	8.59
2	9.45	6.05	9.89

3	9.31	12.95	10.21
4	10.25	15.51	7.89
5	8.28	9.90	8.31
6	7.85	11.76	11.47
7	7.82	7.69	11.18
8	8.51	7.72	13.48
9	10.26	15.31	8.84
10	10.42	5.57	12.57
11	9.31	10.57	9.45
12	5.56	11.46	12.87
13	9.07	5.47	7.20
14	8.76	10.29	10.91
15	7.81	5.38	13.36
16	8.87	12.35	8.73
17	9.56	10.88	10.97
18	10.33	10.48	8.40
19	9.29	16.39	11.34
20	10.22	6.20	9.46
21	9.42	7.12	7.55
22	8.70	8.74	9.61
23	9.51	11.11	9.46
24	9.02	6.19	6.97
25	10.01	10.01	8.94
26	9.91	10.41	12.68
27	9.61	12.29	10.52
28	8.57	7.70	7.89
29	8.92	9.47	9.19
30	8.72	7.24	10.00
**avg.±s.d.**	9.09±0.86	9.59±3.06	9.95±1.81

The trained ensemble models were subsequently employed to predict the %*ee* for the new unseen reactions featuring newly generated chiral (amino acid) ligands. The specifics of these reactions are provided in Table 1 and Fig. 6 in the main body the manuscript. The table below presents the mean predicted %*ee* values along with their standard deviations, derived from 30 independent models within each regressor category. Additionally, the root mean square error (RMSE) and R^2 values, calculated between the predicted and experimental %*ee*, are also reported. These findings highlight the superior performance of our TL-based **EnP** model as compared to the alternative models.

Table S24. Comparison of the Predicted %*ee* Obtained using Different Regressors with the Experimental %*ee* for Newly Generated Reactions

Entry	**EnP**	DNN	RF	AttentiveFP	T5Chem	Experimental %*ee*
1	94.32±2.77	88.87±3.44	84.52±3.84	103.61±9.72	92.37±1.46	94
2	94.19±2.72	88.49±3.18	84.46±4.00	104.49±9.91	92.26±1.51	94
3	92.66±3.36	86.35±5.27	83.08±3.61	89.43±6.68	90.18±1.57	86
4	92.57±3.49	85.99±5.38	83.01±3.56	91.03±7.03	91.92±1.49	85
5	90.55±3.04	88.68±3.42	84.86±3.24	90.02±8.46	91.95±1.55	86
6	90.29±3.43	88.41±3.21	84.67±3.43	90.69±8.33	92.01±1.57	85
7	89.84±3.41	88.73±3.25	85.45±3.56	89.77±8.04	91.90±1.55	86
8	85.50±2.33	83.13±4.12	77.96±5.72	88.64±6.78	87.84±2.02	80
9	84.43±2.61	83.13±4.13	78.21±5.78	88.64±6.78	87.62±2.06	81
10	45.79±5.68	46.29±12.42	49.08±9.39	42.26±11.56	54.09±9.23	30
11	35.31±5.93	39.33±11.73	44.16±8.36	44.77±10.71	46.85±9.07	23
12	89.64±3.92	89.34±3.27	87.89±4.35	86.11±3.09	91.13±2.77	90
13	89.87±2.82	90.11±3.63	87.47±4.23	87.55±3.33	90.83±2.59	91
14	86.94±3.01	87.04±5.92	85.25±3.61	86.84±3.46	90.76±2.83	87
15	86.07±3.00	87.38±3.17	87.21±3.01	86.21±2.47	87.43±3.10	89
RMSE	**6.42**	6.50	8.34	8.58	9.88	
R^2	**0.93**	0.90	0.84	0.88	0.83	

11. Various benchmark models for the generative tasks

To emphasize the advantages of using a transfer learning-based model for generating chiral ligands suitable for the β-C–H activation reactions, we have undertaken thorough benchmarking studies by consiering different generative models. Each model produced 500 molecules, which was then evaluated based on three criteria: validity, uniqueness, and novelty (see Table S24). We applied a series of filters to ensure that the generated ligands met essential standards for catalytic activity. These criteria included the presence of a chiral center (with no more than two chiral centers) and key functional groups, such as the –NHCO fragment, which aids in binding of the transition metal. During our evaluation, we noted that one baseline model (virtual screening) contained atom types of no interest for this class of reaction, including isotopes and elements like [SeH], [V], [D], and [C^{13}]. To maintain chemical relevance, we implemented an additional elemental filter that restricted ligands to only the following atoms: C, H, O, N, S, F, Cl, Br, I, and P. This step ensured the likelihood of the generated ligands adhereding to similar reaction mechanisms as known in the case of experimentally reported catalysts. Such an approach would thereby improve the realism and

reliability of our predictions. Finally, we assessed the Fréchet ChemNet Distance (FCD) of the filtered molecules in comparison to experimentally known chiral ligands, providing a quantitative measure of their structural similarity.[17] The FCD is a commonly used metric for assessing the quality of generated molecules by comparing their distribution to that of real molecules. A lower FCD value indicates that the generated molecules closely resemble real molecules in terms of their chemical properties.

The first generative model considered as a baseline in our study is genetic algorithm (GA), designed to generate chiral ligands by optimizing a scoring function that primarily assesses the validity of the generated molecules.[18] Valid molecules receive a score of 1, while invalid ones 0. In this framework, the GA simulates natural evolution, beginning with an initial population of molecules represented as SMILES strings. The algorithm evaluates the fitness of each molecule based on its validity. High-fitness molecules are selected for reproduction, during which crossover combines substructures while mutations help introduce random changes. This iterative process refines the population, efficiently exploring chemical spaces to discover novel and valid compounds.

The second baseline model is a data-efficient, graph-based generative model (DEG) introduced by Guo et al.[19] It can be trained on much smaller datasets than those typically used in benchmark studies. Central to this model is a learnable graph grammar that automatically generates production rules from the training data, without human input. Optimization of the grammar also allows for integrating additional chemical knowledge into the model.

The third baseline model focuses on virtual screening (VS), in which we used the PubChem database to find possible chiral ligands. Initially, we gathered data that contained the MPAA ligands or structurally similar compounds. After applying the previously

mentioned criteria to filter these molecules, we evaluated their performance shown in the following table.

Table S24. Comparison of the Performance of each Individual Generative Model

model	validity(%)	uniqueness(%)	novelty(%)	number of practical chiral ligands	FCD
GA	100±0.0	100±0.0	87.3±0.4	57.0±7.0	18.6±2.3
DEG	100±0.0	100±0.0	100±0.0	43.3±18.8	18.7±3.8
**FnG (our model)**	**99.3±0.5**	**97.7±0.5**	**97.5±0.7**	**68.3±15.4**	**4.1±0.4**
VS	100.0	100.0	100.0	83.0	32.3

This performance comparison demonstrates that TL-based methods outperform other generative models in generating ligands similar to the experimental chiral ligands in the training set, as indicated by a low FCD score.

12. Choice of %*ee* vs. $\Delta\Delta G^{\ddagger}$ in modeling enantioselectivity

In regression settings, we have used enantiomeric excess (%*ee*) as the primary target variable instead of the free energy difference between transition states ($\Delta\Delta G^{\ddagger}$), due to its direct relevance for synthetic chemistry.[20] While $\Delta\Delta G^{\ddagger}$ offers a kinetic basis for selectivity, %*ee* serves as the practical measure used in synthesis, as it clearly reflects the ratio of major to minor enantiomers. To ensure a comprehensive evaluation, we report model performance for both %*ee* and $\Delta\Delta G^{\ddagger}$ in Table S25. The results indicate that the **EnP** model captures enantioselectivity trends more effectively when trained on %*ee* rather than $\Delta\Delta G^{\ddagger}$. Although we recognize the theoretical importance of $\Delta\Delta G^{\ddagger}$, %*ee* remains the more interpretable and practically actionable metric for guiding reaction optimization.

Table S25. Performance of EnP Regressor in terms of RMSE and R^2 across Different Splits

output	train RMSE	validation RMSE	test (canonical) RMSE	test (TTA) RMSE	test (canonical) R^2	test (TTA) R^2
%*ee*	6.84±0.36	7.76±1.74	7.80±1.23	7.57±1.31	**0.64±0.13**	**0.65±0.15**
$\Delta\Delta G^{\ddagger}$	0.28±0.01	0.37±0.05	0.36±0.04	0.36±0.04	0.53±0.11	0.53±0.12

13. Experimental versus EnP Predicted %*ee* Parity Plot

To evaluate the performance of the **EnP** regressor, we calculated the R^2 metric based on the parity plot that compares experimental and predicted %*ee* values from the test split, utilizing predictions from all 30 trained regressors (**M1–M30**). Our dataset consists of 220 unique reactions, but due to the randomized selection of training and test splits, each reaction may be predicted different number of times across these models.

For instance, a reaction (R1) might appear in the test set of six different models, leading to six distinct predictions. Another another reaction (R2) might appear in only one test set, leading to one prediction. Instead of using all the predictions available for a given reaction, we selected a representative prediction for each reaction to form **a unique reaction set**. The choice of the representative prediction is made on the basis of top-1, top-2, or other ranking-based strategies.[21]

The top-1 R^2 metric is computed by selecting, for each unique reaction, the prediction with the smallest absolute error compared to the experimental %*ee*. In cases where only one prediction was available for a given reaction, it was automatically considered the 'best' entry. Using this approach, we obtained a top-1 R^2 value of 0.89, indicating a strong correlation between predicted and actual %*ee* values. For additional insights, we also provide top-2 to top-5 R^2 values, which ranged from 0.81 to 0.60 (see Table S26). Instead of top-*k*, if the mean value of predicted %*ee* for every unique reaction is used, the resulting R^2 is 0.75. These correlations suggest that our **EnP** offers good confidence in predicting %*ee*.

Table S26. Performance of **EnP** Regressor in terms of R^2

top-*k*	R^2
top-1	0.89
top-2	0.81
top-3	0.75
top-4	0.67
top-5	0.60
mean	0.75

14. Distribution of %yield/%*ee*

The distribution of %yield/%*ee* in three data sets (a) NiCOlit, [22] (b) ELN, [23] and (c) our *β*-C(sp³)–H are provided in Fig. S11. The histogram plots bearing the mean and standard deviation markers provide a clear visualization of the data spread. Notably, our β-C(sp³)–H data set exhibits a strong skewness towards high %*ee*.

(a)

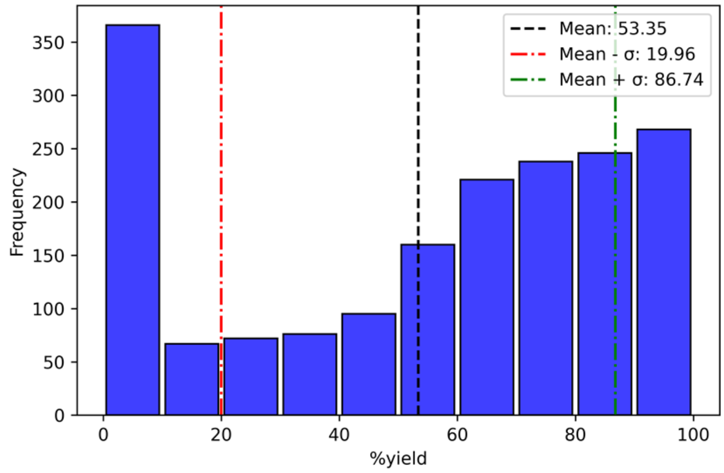

(b)

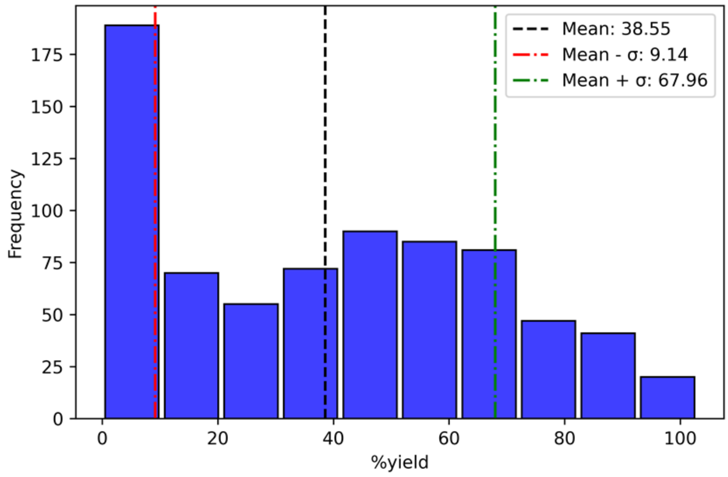

(c)

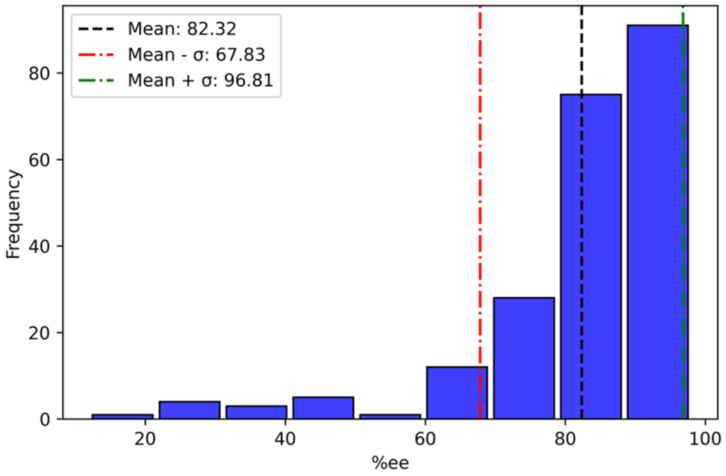

Fig. S11. Distribution of %yield/%*ee* in different data sets (a) NiCOlit, (b) ELN, and (c) our *β*-C(sp³)–H.

15. References

[1] Shen, P.-X.; Hu, L.; Shao, Q.; Hong, K.; Yu, J.-Q. Pd(II)-Catalyzed Enantioselective C(Sp3)–H Arylation of Free Carboxylic Acids. *J. Am. Chem. Soc.* **2018**, *140*, 6545–6549.

[2] Hu, L.; Shen, P.; Shao, Q.; Hong, K.; Qiao, J. X.; Yu, J. PdII-Catalyzed Enantioselective C(Sp3)−H Activation/Cross-Coupling Reactions of Free Carboxylic Acids. *Angew. Chem. Int. Ed.* **2019**, *58*, 2134–2138.

[3] Xiao, K.-J.; Lin, D. W.; Miura, M.; Zhu, R.-Y.; Gong, W.; Wasa, M.; Yu, J.-Q. Palladium(II)-Catalyzed Enantioselective C(Sp3)–H Activation Using a Chiral Hydroxamic Acid Ligand. *J. Am. Chem. Soc.* **2014**, *136*, 8138–8142.

[4] Wu, Q.-F.; Wang, X.-B.; Shen, P.-X.; Yu, J.-Q. Enantioselective C–H Arylation and Vinylation of Cyclobutyl Carboxylic Amides. *ACS Catal.* **2018**, *8*, 2577–2581.

[5] Howard, J.; Ruder, S. Universal Language Model Fine-tuning for Text Classification. in *Proceedings of the 56th Annual Meeting of the Association for Computational Linguistics (Volume 1: Long Papers)* (Association for Computational Linguistics, 2018).

[6] Li, J.; Tang, T.; Zhao, W. X.; Nie, J.-Y.; Wen, J.-R. Pre-Trained Language Models for Text Generation: A Survey. *ACM Comput. Surv.* **2024**, *56*, 1–39.

[7] Weininger, D. SMILES, a Chemical Language and Information System. 1. Introduction to Methodology and Encoding Rules. *J. Chem. Inf. Comput. Sci.* **1988**, *28*, 31–36.

[8] Singh, S.; Sunoj, R. B. A Transfer Learning Protocol for Chemical Catalysis Using a Recurrent Neural Network Adapted from Natural Language Processing. *Digital Discovery* **2022**, *1*, 303–312.

[9] Singh, S.; Sunoj, R. B. A Transfer Learning Approach for Reaction Discovery in Small Data Situations Using Generative Model. *iScience* **2022**, *25*, 104661.

[10] Santana, M. V. S.; Silva-Jr, F. P. De Novo Design and Bioactivity Prediction of SARS-CoV-2 Main Protease Inhibitors Using Recurrent Neural Network-Based Transfer Learning. *BMC Chem.* **2021**, *15*, 8.

[11] Moret, M.; Friedrich, L.; Grisoni, F.; Merk, D.; Schneider, G. Generative Molecular Design in Low Data Regimes. *Nat. Mach. Intell.* **2020**, *2*, 171–180.

[12] McInnes, L.; Healy, J.; Saul, N.; Großberger, L. UMAP: Uniform Manifold Approximation and Projection. *J. Open Source Softw.* **2018**, *3*, 861.

[13] Probst, D.; Reymond, J.-L. Visualization of Very Large High-Dimensional Data Sets as Minimum Spanning Trees. *J. Cheminform.* **2020**, *12*, 12.

[14] Akiba, T.; Sano, S.; Yanase, T.; Ohta, T.; Koyama, M. Optuna. In *Proceedings of the 25th ACM SIGKDD International Conference on Knowledge Discovery & Data Mining*; ACM: New York, NY, USA, **2019**; pp 2623–2631.

[15] Xiong, Z.; Wang, D.; Liu, X.; Zhong, F.; Wan, X.; Li, X.; Li, Z.; Luo, X.; Chen, K.; Jiang, H.; Zheng, M. Pushing the Boundaries of Molecular Representation for Drug Discovery with the Graph Attention Mechanism. *J Med Chem.* **2019**, *63*, 8749–8760.

[16] Lu, J.; Zhang, Y. Unified Deep Learning Model for Multitask Reaction Predictions with Explanation. *J. Chem. Inf. Model.* **2022**, *62*, 1376–1387.

[17] Preuer, K.; Renz, P.; Unterthiner, T.; Hochreiter, S.; Klambauer, G. Fréchet ChemNet Distance: A Metric for Generative Models for Molecules in Drug Discovery. *J. Chem. Inf. Model.* **2018**, *58*, 1736–1741.

[18] Jensen, J. H. A Graph-Based Genetic Algorithm and Generative Model/Monte Carlo Tree Search for the Exploration of Chemical Space. *Chem. Sci.* **2019**, *10*, 3567–3572.

[19] Guo, M.; Thost, V.; Li, B.; Das, P.; Chen, J.; Matusik, W. Data-Efficient Graph Grammar Learning for Molecular Generation. *International Conference on Learning Representations* **2021**.

[20] Ruth, M.; Gensch, T.; Schreiner, P. R. Contrasting Historical and Physical Perspectives in Asymmetric Catalysis: ΔΔG≠ versus Enantiomeric Excess. *Angew. Chem. Int. Ed.* **2024**, *63*, e202410308.

[21] J. Schleinitz, A. Carretero-Cerdán, A. Gurajapu, Y. Harnik, G. Lee, A. Pandey, A. Milo and S. E. Reisman, *J. Am. Chem. Soc.*, 2025, **147**, 7476–7484.

[22] J. Schleinitz, *et al.*, Machine Learning Yield Prediction from NiCOlit, a Small-Size Literature Data Set of Nickel Catalyzed C–O Couplings. *J. Am. Chem. Soc.*, **2022**, 144, 14722–14730.

[23] M. Saebi, *et al.*, On the use of real-world datasets for reaction yield prediction. *Chem. Sci.* **2023**, 14, 4997–5005.

Part II

Table of Contents

section	content	page number
1	General information	S46
2	Ligand structures	S47
3	Experimental section	S47-S62
	3.1 Synthesis of ligands	
	3.1.1 Preparation of N-Ac MPAHA ligands	
	3.1.2 Preparation of N-Ac MPAAM Ligand	
	3.1.3 Preparation of N-Ac MPAO Ligand	
	3.2 General Procedure for C–H Activation Reactions	
	3.2.1 Enantioselective arylation of cyclopropanecarboxylic acid by ArI	
	3.2.2 Enantioselective arylation of cyclopropanecarboxylic acid by ArBpin	
	3.2.3 Enantioselective arylation of cyclobutanecarboxamide by ArBpin	
	3.2.4 Enantioselective arylation of cyclobutanecarboxamide by ArI	
4	References	S62
5	NMR Spectra	S62-S72

1. General Information

Carboxylic acids were obtained from the commercial sources or synthesized following literature procedures. Alkyl iodides were obtained from the commercial sources. Solvents were obtained from Sigma-Aldrich, Oakwood, and Acros and used directly without further purification.

Analytical thin layer chromatography was performed on 0.25 mm silica gel 60-F254. Visualization was carried out with UV light and Bromocresol Green Stain.

^{1}H NMR was recorded on Bruker DRX-600 instrument (600 MHz). Chemical shifts were quoted in parts per million (ppm) referenced to the peak of tetramethylsilane at 0 ppm. The following abbreviations were used to explain multiplicities: s = singlet, d = doublet, t = triplet, q = quartet, p = pentet, m = multiplet, br = broad. Coupling constants *J* were reported in Hertz unit (Hz).

^{13}C NMR spectra were recorded on Bruker DRX-600 instrument (150 MHz) and were fully decoupled by broad band proton decoupling. Chemical shifts were reported in ppm referenced to center line of a triplet at 77.0 ppm of chloroform-*d*.

Enantiomeric ratios (er) were determined on an Agilent SFC system or Waters SFC system using commercially available chiral columns.

2. Ligand Structures

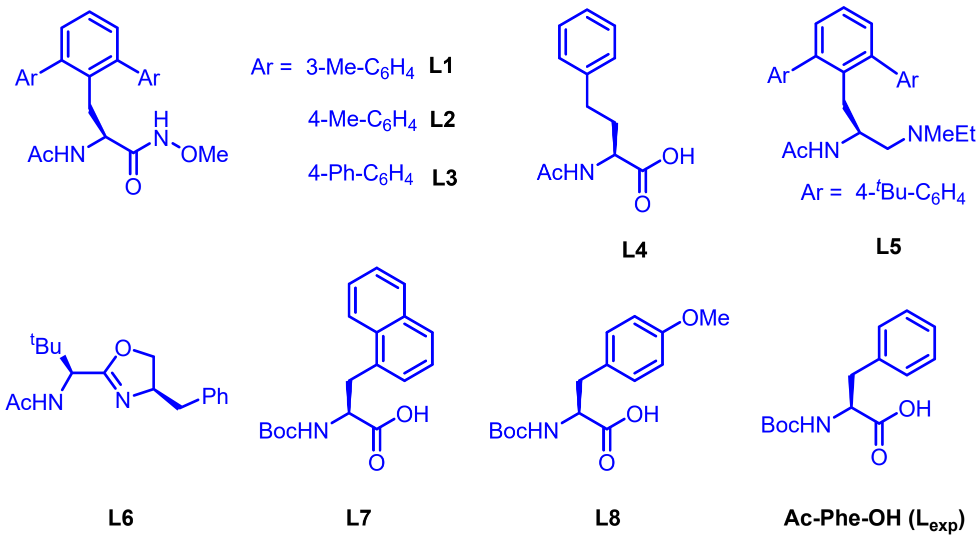

3. Experimental Section

3.1 Synthesis of Ligands

3.1.1 Preparation of N-Ac MPAHA Ligands

General Procedure

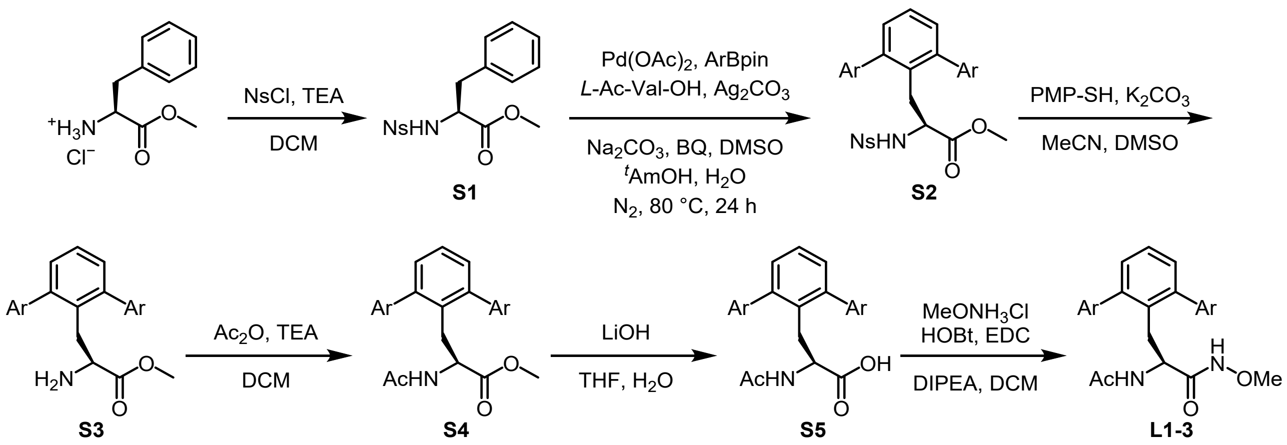

4-Nitrobenzenesulfonyl chloride (NsCl, 50 mmol, 1.0 equiv) was added to a cooled (0 °C) solution of *L*-phenylalanine methyl ester hydrochloride (*L*-Phe-OMe·HCl, 50 mmol, 1.0 equiv) and triethylamine (TEA, 150 mmol, 3.0 equiv) in DCM (150 mL). After being stirred at room temperature for 12 h, the reaction mixture was poured into H_2O. The organic layer was separated, dried over anhydrous Na_2SO_4, filtered and concentrated under vacuum. The residue was purified by trituration with a mixture of 50% DCM/hexane to give **S1**.

S1 (8.0 mmol, 1.0 equiv), $Pd(OAc)_2$ (0.075 equiv), Ar-BPin (2.0 equiv), *L*-Ac-Val-OH (0.2 equiv), Ag_2CO_3 (2.0 equiv), Na_2CO_3 (2.0 equiv), BQ (0.5 equiv), H_2O (5.0 equiv), and DMSO (0.4 equiv) were weighed in air and placed in a Schlenk tube with a magnetic stir bar. *t*-AmylOH (50 mL) was added, and the reaction vessel was evacuated and backfilled with nitrogen for three times. The reaction mixture was heated to 80 °C for 24 h under vigorous stirring. After being cooled to room temperature, the reaction mixture was diluted with EtOAc and filtered through a pad of Celite eluting with EtOAc. The filtrate was concentrated under vacuum and the resulting residue was purified by flash chromatography on silica gel (eluent: EtOAc/hexane = 1:3) to give **S2**.

4-Methoxybenzenethiol (PMP–SH, 20.0 mmol, 4.0 equiv) and potassium carbonate (20.0 mmol, 4.0 equiv) were added to a solution of **S2** (5.0 mmol, 1.0 equiv) in MeCN (40 mL) and DMSO (1.5 mL). After being stirred at room temperature for 12 h, the reaction mixture was diluted with EtOAc, washed with H_2O and brine, dried over anhydrous Na_2SO_4, filtered, and concentrated under vacuum. The residue was purified by column chromatography on silica gel (eluent: EtOAc/hexane = 1:1) to give **S3**.

Ac_2O (12.0 mmol, 3.0 equiv) was added to a solution of **S3** (4.0 mmol, 1.0 equiv) and triethylamine (12.0 mmol, 3.0 equiv) in DCM (20 mL). After being stirred at room temperature for 3 h, the reaction mixture was quenched with saturated NH_4Cl solution and extracted with DCM. The combined organic layers were dried over anhydrous Na_2SO_4, filtered and concentrated to give **S4**, which could be used directly in the next step without further purification.

LiOH (8.0 mmol) was added to a suspension of **S4** in THF (8 mL), and H_2O (4 mL) at 0 °C. The reaction mixture was allowed to warm to room temperature and stirred for 8 h. The reaction mixture was quenched with 10% aqueous citric acid solution and extracted EtOAc (3 × 20 mL). The combined organic layers were washed with brine, dried over anhydrous

Na_2SO_4, filtered and concentrated to give **S5**, further purification could be conducted by recrystallization or reversed phase flash column.

O-Alkylhydroxylamine hydrochloride salt (1.5 equiv) and *N*,*N*-diisopropylethylamine (DIPEA, 1.5 equiv) were added to a cooled (0 °C) solution of **S5** (1.0 equiv), HOBt (1.1 equiv) and EDC (1.1 equiv) in DCM (0.25 M). After being stirred at room temperature for 8 h, the reaction mixture was poured into H_2O. The organic layer was separated, dried over anhydrous Na_2SO_4, filtered and concentrated under vacuum. The residue was purified by column chromatography on silica gel (eluent: MeOH/DCM = 1:20) to give the corresponding ligands **L1-3**.

(*S*)-2-acetamido-3-(3,3''-dimethyl-[1,1':3',1''-terphenyl]-2'-yl)-N-methoxypropanamide (L1)

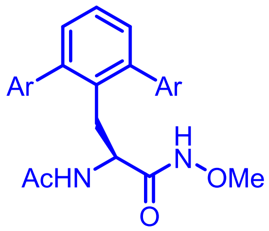

L1 was synthesized following the standard procedure as a white solid and purified by reverse phase column.

^{1}H NMR (600 MHz, CDCl$_3$): δ 7.48 (s, 1H), 7.32 – 7.25 (m, 10H), 7.20 (d, J = 7.6 Hz, 2H), 5.15 (d, J = 8.0 Hz, 1H), 3.86 – 3.79 (m, 1H), 3.49 (s, 3H), 3.09 (s, 1H), 3.08 (s, 1H), 2.43 (s, 6H), 1.73 (s, 3H).

HRMS (ESI-TOF): *m*/*z* Calcd for $C_{26}H_{29}N_2O_3^+$ [M+H]$^+$ 417.2178, found 417.2188.

(*S*)-2-acetamido-3-(4,4''-dimethyl-[1,1':3',1''-terphenyl]-2'-yl)-N-methoxypropanamide (L2)

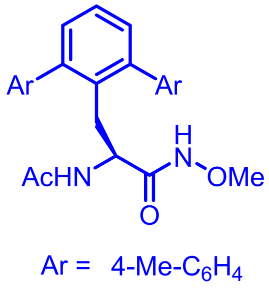

L2 was synthesized following the standard procedure as a white solid and purified by reverse phase column.

^{1}H NMR (600 MHz, CDCl$_3$): δ 7.48 (s, 1H), 7.32 – 7.25 (m, 10H), 7.20 (d, J = 7.6 Hz, 2H), 5.15 (d, J = 8.0 Hz, 1H), 3.86 – 3.79 (m, 1H), 3.49 (s, 3H), 3.09 (s, 1H), 3.08 (s, 1H), 2.43 (s, 6H), 1.73 (s, 3H).

^{13}C NMR (151 MHz, CDCl$_3$): δ 170.02, 168.60, 142.93, 138.85, 137.21, 132.50, 129.65, 129.52, 129.33, 126.67, 64.09, 51.63, 31.08, 22.93, 21.13.

HRMS (ESI-TOF): *m/z* Calcd for $C_{26}H_{29}N_2O_3^+$ [M+H]$^+$ 417.2178, found 417.2183.

(*S*)-2-acetamido-3-(4,4''-diphenyl-[1,1':3',1''-terphenyl]-2'-yl)-N-methoxypropanamide (L3)

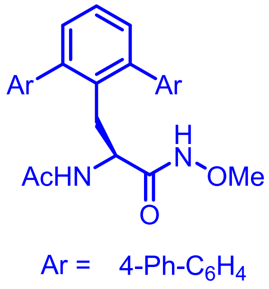

L2 was synthesized following the standard procedure as a white solid and purified by reverse phase column.

^{1}H NMR (600 MHz, CDCl$_3$): δ 7.75 – 7.71 (m, 4H), 7.69 – 7.65 (m, 4H), 7.61 (d, J = 2.4 Hz, 1H), 7.51 – 7.46 (m, 8H), 7.42 – 7.36 (m, 3H), 7.29 (d, J = 7.6 Hz, 2H), 5.24 (d, J = 8.2 Hz, 1H), 3.93 (td, J = 8.7, 4.3 Hz, 1H), 3.38 (s, 3H), 3.20 (m, 2H), 1.74 (s, 3H).

^{13}C NMR (151 MHz, $CDCl_3$) δ 170.07, 168.55, 142.73, 140.68, 140.47, 140.24, 132.42, 130.12, 129.89, 128.93, 127.64, 127.36, 127.08, 126.91, 64.17, 51.56, 31.31, 23.00.

HRMS (ESI-TOF): *m/z* Calcd for $C_{36}H_{33}N_2O_3^+$ $[M+H]^+$ 541.2491, found 541.2504.

3.1.2 Preparation of *N*-Ac MPAAM Ligand

General Procedure

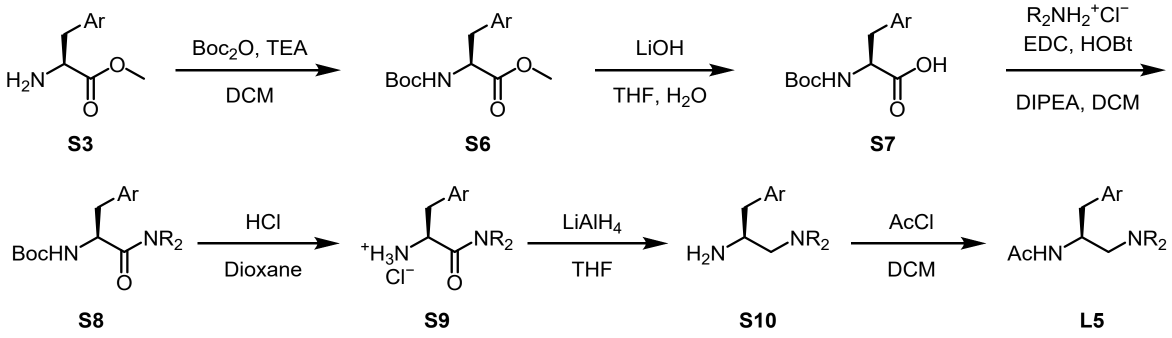

Boc_2O (8.0 mmol, 2.0 equiv) was added to a solution of **S3** (4.0 mmol, 1.0 equiv) and triethylamine (8.0 mmol, 2.0 equiv) in DCM (20 mL). After being stirred at room temperature for 3 h, the reaction mixture was quenched with saturated NH_4Cl solution and extracted with DCM. The combined organic layers were dried over anhydrous Na_2SO_4, filtered and concentrated to give **S6**, which could be used directly in the next step without further purification.

LiOH (8.0 mmol) was added to a suspension of **S6** in THF (8 mL), and H_2O (4 mL) at 0 °C. The reaction mixture was allowed to warm to room temperature and stirred for 8 h. The reaction mixture was quenched with 10% aqueous citric acid solution and extracted EtOAc (3 × 20 mL). The combined organic layers were washed with brine, dried over anhydrous Na_2SO_4, filtered**,** and concentrated to give **S7**, further purification could be conducted by recrystallization or reversed phase flash column.

The corresponding Boc-protected amino acid **S7** (4 mmol), dialkylammonium chloride (8.8 mmol, 2.2 equiv) and benzotriazol-1-ol hydrate (HOBt) (4 mmol, 1.0 equiv) were added to a round bottom flask equipped with a magnetic stir bar. The solid mixture was dissolved in

DCM (40 mL), and 1-ethyl-(3-(3-dimethylamino)propyl)-carbodiimide hydrochloride (EDC) (4.8 mmol, 1.2 equiv) was added at 0 °C. The resulting solution was stirred at 0 °C as *N*-ethyl-*N*,*N*-diisopropylamine (DIPEA) (9.6 mmol, 2.4 equiv) was added slowly. The reaction solution was allowed to warm to r.t. and stirred for about 3 h, after which the solution was poured into a separatory funnel, diluted to 150 mL with additional DCM, and washed with approximately 25 mL of 10% *w*/*w* aqueous citric acid. The organic layer was separated and subsequently washed with 25 mL each of saturated aqueous $NaHCO_3$ and brine. The organics were dried over anhydrous Na_2SO_4, filtered, and concentrated in vacuo to provide corresponding amide **S8** which could be directly used in the next step without further purification.

To the Boc-protected amino amide **S8** was added 4 N HCl/dioxane solution (4 mL). The resulting solution was stirred at room temperature for 2 h. Then, the volatile components were evaporated in vacuo, and the residue was subsequently used in the following reduction step.

To a solution of **S9** in THF (24 mL) was added a solution of LiAlH4 in THF (6.0 mmol, 1.5 equiv) dropwise under N_2 at 0 °C. Then, the mixture was heated to reflux for 12 h, before being cooled down and diluted with ether. The mixture was cooled to 0 °C, and 0.28 mL of water was added slowly followed by 15% w/w NaOH aqueous solution (0.28 mL) and water (0.84 mL). The resulting suspension was then warmed to room temperature and stirred for 15 min before $MgSO_4$ was added. The mixture was stirred for additional 15 min before filtration. The filtrate was collected, and the solvent was removed in vacuo to provide diamine compound **S10** which could be used in the next step without purification.

To a solution of the synthesized diamine compound **S10** in DCM 8 mL was added acetyl chloride (8 mmol, 2.0 equiv) at 0 °C. Then the solution was stirred at room temperature for 2 h. The volatile components were evaporated in vacuo, and the residue was dissolved in 8 ml

of water. The resulting solution was extracted with ether (10 mL × 3), then the aqueous phase was alkalized with 15% *w*/*w* NaOH aqueous solution until pH > 13. The alkalized mixture was extracted with ether (10 ml × 3), and the organic layers were concentrated to provide the desired MPAAM Ligand **L5**. Further purification could be conducted by recrystallization or reversed phase flash column.

(*S*)-*N*-(1-(4,4''-di-tert-butyl-[1,1':3',1''-terphenyl]-2'-yl)-3-(ethylmethylamino)propan-2-yl)acetamide (L5)

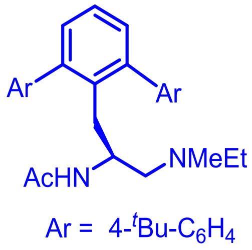

L5 was synthesized following the standard procedure as a white solid and purified by reverse phase column.

^{1}H NMR (600 MHz, CDCl$_3$): δ 7.40 – 7.38 (m, 4H), 7.28 – 7.25 (m, 4H), 7.19 – 7.16 (m, 1H), 7.09 (d, J = 7.5 Hz, 2H), 4.59 (m, 1H), 3.75 – 3.64 (m, 1H), 3.11 (dd, J = 14.3, 3.5 Hz, 1H), 2.67 (dd, J = 14.3, 11.1 Hz, 1H), 1.95 (m, 3H), 1.67 – 1.60 (m, 7H), 1.30 (s, 18H), 0.70 (t, J = 7.1 Hz, 3H).

HRMS (ESI-TOF): *m*/*z* Calcd for $C_{34}H_{47}N_2O^+$ $[M+H]^+$ 499.3688, found 499.3685.

3.1.3 Preparation of *N*-Ac MPAO Ligand

General Procedure

To the solution of *N*-Fmoc-protected amino acid (10.0 mmol) in DCM (200 mL), amino alcohol (10.0 mmol), PPh_3 (30.0 mmol) and *N*,*N*-diisopropylethylamine (DIPEA) (30.0 mmol) were added at 0 °C. CCl_4 (50.0 mmol) was added dropwise over three hours via a syringe pump. The ice bath was removed after the addition, and the reaction mixture was stirred at room temperature for 24 h. The solvents were then removed under reduced pressure.

The residue was purified by silica gel column chromatography to afford the oxazoline intermediate **S11**.

To the solution of *N*-Fmoc-protected oxazoline **S11** (6.0 mmol) in MeOH (30 mL), piperidine (12.0 mmol) was added dropwise at 0 °C. The ice bath was removed after the addition, and the reaction was stirred at room temperature. The reaction progress was monitored by TLC within 2 hours. After completion, the solvents were removed under reduced pressure, and the residue was purified by silica gel column chromatography to afford the desired amine intermediate **S12**.

Triethylamine (4.5 mmol) was added to the solution of the amine **S12** (3.0 mmol) in dry DCM (20 mL). Acetic anhydride (4.5 mmol) was added slowly in 10 minutes at 0 °C. The ice bath was removed after the addition, and the reaction was stirred at room temperature. The reaction progress was monitored by TLC. After completion, the reaction was quenched at 0 °C with saturated $NaHCO_3$ (aq). The layers were separated, and the aqueous layer was extracted with DCM. The combined organic layers were dried over anhydrous Na_2SO_4, filtered, and concentrated in vacuo. The crude reaction mixture was purified by silica gel column chromatography to afford the desired MPAO ligand **L6**.

***N*-((*S*)-1-((*R*)-4-benzyl-4,5-dihydrooxazol-2-yl)-2,2-dimethylpropyl)acetamide (L6)**

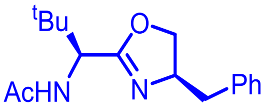

^{1}H NMR (600 MHz, MeOD) δ 7.34 (t, J = 7.6 Hz, 2H), 7.29 – 7.25 (m, 3H), 4.17 (s, 1H), 3.67 (dd, J = 11.6, 3.5 Hz, 1H), 3.50 (dd, J = 11.6, 6.3 Hz, 1H), 3.40 (qd, J = 6.7, 3.6 Hz, 1H), 2.94 – 2.87 (m, 2H), 1.99 (s, 3H), 0.99 (s, 9H).

HRMS (ESI-TOF): *m/z* Calcd for $C_{17}H_{25}N_2O_2^+$ $[M+H]^+$ 289.1911, found 289.1915.

3.2 General Procedure for C–H Activation Reactions

3.2.1 Enantioselective arylation of cyclopropanecarboxylic acid by ArI

General Procedure

A 2-dram vial equipped with a magnetic stir bar was charged with $Pd(OAc)_2$ (4.4 mg, 10 mol%) and ligand (20 mol%) in HFIP (0.25 mL). The appropriate cyclopropanecarboxylic acid substrate (0.20 mmol), Ag_2CO_3 (82.7 mg, 0.30 mmol), Na_2CO_3 (31.8 mg, 0.30 mmol) and aryl iodide (0.40 mmol) were then added. Subsequently, the vial was capped and closed tightly. The reaction mixture was then stirred at the rate of 300 rpm at 80 °C for 16 h.

After being allowed to cool to room temperature, the mixture was diluted with ethyl acetate, and 0.1 ml of acetic acid was then added. The mixture was passed through a pad of Celite with ethyl acetate as the eluent to remove any insoluble precipitate. The resulting solution was concentrated, and the residual mixture was dissolved with a minimal amount of acetone and loaded onto a preparative TLC plate. The pure product was then isolated using preparative TLC with ethyl acetate and hexanes as the eluent and 2% *v*/*v* of acetic acid as the additive.

(1*R*,2*S*)-2-(3-chlorophenyl)cyclopropane-1-carboxylic acid (S13a)

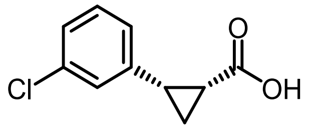

The reaction was completed following the general procedure with the ligand **L5** (eluent: hexanes/ethyl acetate = 5/1 with 2% *v*/*v* of acetic acid). The product was obtained as a slightly yellow oil (59% yield, 85% *ee*).

^{1}H NMR (600 MHz, $CDCl_3$): δ 7.20 (s, 1H), 7.16-7.11 (m, 2H), 7.09-7.06 (m, 1H), 2.57 (m, 1H), 2.05 (m, 1H), 1.67 (dt, J = 7.9, 5.0 Hz, 1H), 1.40 (ddd, J = 8.3, 7.8, 4.7 Hz, 1H).

HRMS (ESI-TOF): *m/z* Calcd for $C_{10}H_{10}ClO_2^+$ $[M+H]^+$ 197.0364, found 197.0372.

(1*R*,2*S*)-2-(3-methoxyphenyl)cyclopropane-1-carboxylic acid (S13b)

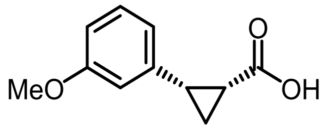

The reaction was completed following the general procedure with the ligand **L5** (eluent: hexanes/ethyl acetate = 5/1 with 2% *v*/*v* of acetic acid). The product was obtained as a slightly yellow oil (57% yield, 86% *ee*).

^{1}H NMR (600 MHz, $CDCl_3$) δ 7.16 (t, J = 7.9 Hz, 1H), 6.86 – 6.81 (m, 1H), 6.80 (t, J = 2.0 Hz, 1H), 6.75 (dd, J = 8.2, 2.6 Hz, 1H), 3.77 (s, 3H), 2.60 (q, J = 8.6 Hz, 1H), 2.08 – 2.00 (m, 1H), 1.65 (dt, J = 7.6, 5.3 Hz, 1H), 1.36 (ddd, J = 8.7, 7.7, 5.1 Hz, 1H).

HRMS (ESI-TOF): *m*/*z* Calcd for $C_{11}H_{13}O_3^+$ $[M+H]^+$ 193.0859, found 193.0865.

3.2.2 Enantioselective arylation of cyclopropanecarboxylic acid by ArBpin

General Procedure

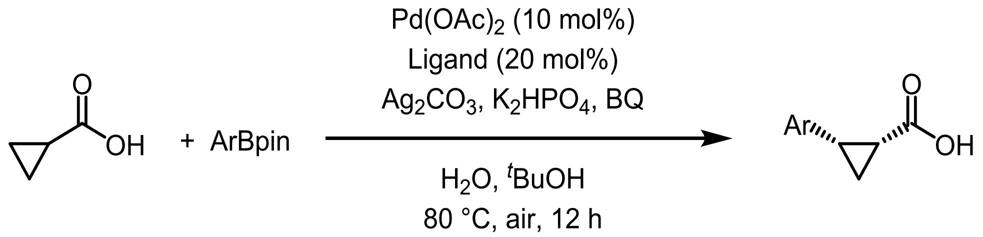

A 2-dram vial equipped with a magnetic stir bar was charged with $Pd(OAc)_2$ (2.2 mg, 10 mol%) and the ligand (9.4 mg, 20 mol%) in ^{t}BuOH (1.0 mL) and then stirred at the rate of 300 rpm at room temperature for 5 min. The appropriate cyclopropanecarboxylic acid substrate (0.10 mmol), Ag_2CO_3 (41.4 mg, 0.15 mmol), K_2HPO_4 (26.0 mg, 0.15 mmol), ArBpin (0.15 mmol), BQ (5.4 mg, 0.05 mmol), H_2O (18.0 mg, 1.0 mmol) were then added. Subsequently the vial was capped and closed tightly. The reaction mixture was then stirred at the rate of 300 rpm at 80 °C for 12 h.

After being allowed to cool to room temperature, the mixture was diluted with ethyl acetate and 0.1 mL of acetic acid was then added. The mixture was passed through a pad of Celite with ethyl acetate as the eluent to remove any insoluble precipitate. The resulting solution was concentrated, and the residual mixture was dissolved with a minimal amount of acetone and loaded onto a preparative TLC plate. The pure product was then isolated using

preparative TLC with ethyl acetate and hexanes as the eluent and 2% *v*/*v* of acetic acid as additive.

(1*R*,2*S*)-2-phenylcyclopropane-1-carboxylic acid (S13c)

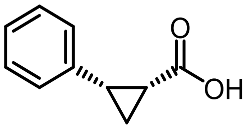

The reaction was completed following the general procedure with **L(Ac-Phe-OH)** as the ligand (eluent: hexanes/ethyl acetate = 5/1 with 2% *v*/*v* of acetic acid). The product was obtained as a white solid (58% yield, 90% *ee*).

The reaction was also performed with ligand **L1**, resulted in no product.

^{1}H NMR (600 MHz, CDCl$_3$): δ 7.26 – 7.19 (m, 5H), 2.63 (q, J = 8.6 Hz, 1H), 2.05 (ddd, J = 9.2, 7.7, 5.6 Hz, 1H), 1.66 (dt, J = 7.6, 5.4 Hz, 1H), 1.37 (td, J = 8.2, 5.0 Hz, 1H).

HRMS (ESI-TOF): *m*/*z* Calcd for $C_{10}H_9O^+$ $[M{-}OH]^+$ 145.0653, found 145.0655.

(1*R*,2*S*)-2-(4-fluorophenyl)cyclopropane-1-carboxylic acid (S13d)

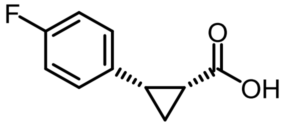

The reaction was completed following the general procedure with the ligand **L(Ac-Phe-OH)** (eluent: hexanes/ethyl acetate = 5/1 with 2% *v*/*v* of acetic acid). The product was obtained as a white solid (55% yield, 91% *ee*).

The reaction was also performed with ligand **L1** or **L2**, resulted in no product.

The reaction was also performed with ligand **L5** (28% yield, 94% *ee*).

^{1}H NMR (600 MHz, CDCl$_3$): δ 7.21 – 7.16 (m, 2H), 6.96 – 6.91 (m, 2H), 2.59 (q, J = 8.5 Hz, 1H), 2.03 (ddd, J = 9.1, 7.7, 5.6 Hz, 1H), 1.62 (dt, J = 7.6, 5.4 Hz, 1H), 1.38 (ddd, J = 8.6, 7.7, 5.2 Hz, 1H).

HRMS (ESI-TOF): *m*/*z* Calcd for $C_{10}H_{10}FO_2^+$ $[M+H]^+$ 181.0665, found 181.0671.

(1*R*,2*S*)-2-(3-trifluoromethylphenyl)cyclopropane-1-carboxylic acid (S13e)

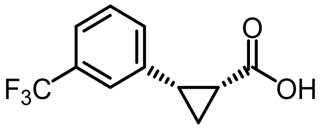

The reaction was completed following the general procedure with the ligand **L(Ac-Phe-OH)** (eluent: hexanes/ethyl acetate = 5/1 with 2% *v*/*v* of acetic acid). The product was obtained as a white solid (62% yield, 87% *ee*).

The reaction was also performed with ligand **L1**, resulted in no product.

^{1}H NMR (600 MHz, $CDCl_3$): δ 7.49 (s, 1H), 7.46 (d, J = 7.8 Hz, 1H), 7.40 (d, J = 7.7 Hz, 1H), 7.35 (t, J = 7.7 Hz, 1H), 2.64 (q, J = 8.5 Hz, 1H), 2.09 (ddd, J = 9.2, 7.8, 5.6 Hz, 1H), 1.68 (dt, J = 7.7, 5.4 Hz, 1H), 1.44 (ddd, J = 8.6, 7.8, 5.2 Hz, 1H).

HRMS (ESI-TOF): *m*/*z* Calcd for $C_{11}H_{10}F_3O_2^+$ $[M+H]^+$ 231.0633, found 231.0639.

(1*R*,2*S*)-2-(3-(methoxycarbonyl)phenyl) cyclopropane-1-carboxylic acid (S13f)

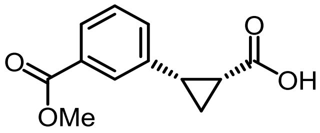

The reaction was completed following the general procedure with the ligand **L(Ac-Phe-OH)** (eluent: hexanes/ethyl acetate = 3/1 with 2% *v*/*v* of acetic acid). The product was obtained as a white solid (58% yield, 89% *ee*).

The reaction was also performed with ligand **L1**, resulted in no product.

^{1}H NMR (600 MHz, $CDCl_3$): δ 7.93 (t, J = 1.8 Hz, 1H), 7.88 (dt, J = 7.6, 1.5 Hz, 1H), 7.40 (ddd, J = 7.7, 2.0, 1.0 Hz, 1H), 7.31 (t, J = 7.7 Hz, 1H), 3.91 (s, 3H), 2.64 (q, J = 8.5 Hz, 1H), 2.07 (ddd, J = 9.1, 7.8, 5.6 Hz, 1H), 1.70 (dt, J = 7.6, 5.4 Hz, 1H), 1.45 – 1.39 (m, 1H).

HRMS (ESI-TOF): *m*/*z* Calcd for $C_{12}H_{13}O_4^+$ $[M+Na]^+$ 243.0633, found 243.0639.

(1*R*,2*S*)-2-(4-chlorophenyl)cyclopropane-1-carboxylic acid (S13g)

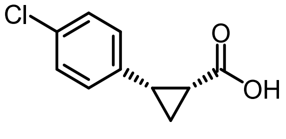

The reaction was completed following the general procedure with the ligand **L5** (eluent: hexanes/ethyl acetate = 5/1 with 2% *v*/*v* of acetic acid). The product was obtained as a white solid (33% yield, 94% *ee*).

The reaction was also performed with ligand **L4** (45% yield, 85% *ee*).

^{1}H NMR (600 MHz, CDCl$_3$): δ 7.22 (d, J = 8.3 Hz, 2H), 7.16 (d, J = 8.3 Hz, 2H), 2.57 (q, J = 8.6 Hz, 1H), 2.09 – 2.04 (m, 1H), 1.63 (dt, J = 7.6, 5.4 Hz, 1H), 1.41 – 1.37 (m, 1H).

HRMS (ESI-TOF): *m*/*z* Calcd for $C_{10}H_{10}ClO_2^+$ $[M+H]^+$ 197.0369, found 197.0365.

(1*R*,2*S*)-2-(4-trifluoromethoxy)cyclopropane-1-carboxylic acid (S13h)

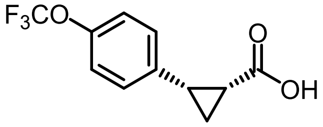

The reaction was completed following the general procedure with the ligand **L4** (eluent: hexanes/ethyl acetate = 4/1 with 2% *v*/*v* of acetic acid). The product was obtained as a white solid (39% yield, 86% *ee*).

^{1}H NMR (600 MHz, CDCl$_3$): δ 7.22 (d, J = 8.2 Hz, 1H), 7.08 (d, J = 8.2 Hz, 1H), 1.63 (dt, J = 7.5, 5.3 Hz, 1H), 1.39 (td, J = 8.1, 5.1 Hz, 1H).

HRMS (ESI-TOF): *m*/*z* Calcd for $C_{11}H_{10}F_3O_3^+$ $[M+H]^+$ 247.0582, found 247.0587.

(1*R*,2*S*)-2-(4-methylphenyl)cyclopropane-1-carboxylic acid (S13i)

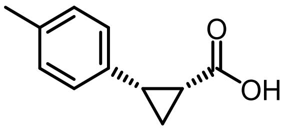

The reaction was completed following the general procedure with the ligand **L4** (eluent: hexanes/ethyl acetate = 5/1 with 2% *v*/*v* of acetic acid). The product was obtained as a white solid (47% yield, 86% *ee*).

^{1}H NMR (600 MHz, CDCl$_3$): δ 7.12 (d, J = 7.7 Hz, 2H), 7.05 (d, J = 7.7 Hz, 2H), 2.58 (q, J = 8.6 Hz, 1H), 2.31 (s, 3H), 2.05 – 1.97 (m, 1H), 1.63 (dt, J = 7.6, 5.3 Hz, 1H), 1.38 – 1.32 (m, 1H).

HRMS (ESI-TOF): *m/z* Calcd for $C_{11}H_{13}O_2^+$ $[M+H]^+$ 177.0910. 159.0810\ 159.0815

3.2.3 Enantioselective arylation of cyclobutanecarboxamide by ArBpin

General Procedure

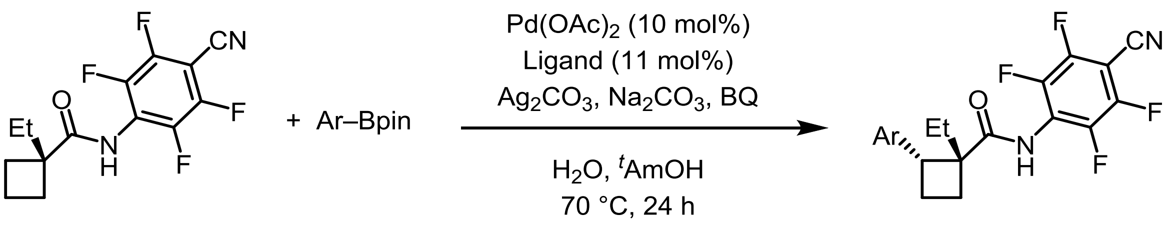

Amide substrate (0.1 mmol, 1.0 equiv), $Pd(OAc)_2$ (0.1 equiv), ArBPin (2.0 equiv), ligand (0.11 equiv), Ag_2CO_3 (2.5 equiv), Na_2CO_3 (2.0 equiv), BQ (0.5 equiv), H_2O (5.0 equiv) and ^{t}AmylOH (0.5 mL) were added into a 10 mL sealed tube. The reaction vessel was evacuated and backfilled with nitrogen (×3). The reaction mixture was heated to 70 °C for 24 h under vigorous stirring.

After being cooled to room temperature, the reaction mixture was diluted with EtOAc and filtered through a pad of Celite, eluting with EtOAc. The filtrate was concentrated under vacuum and the resulting residue was purified by preparative TLC using EtOAc/hexanes as the eluent to give the desired product.

(1*R*,2*R*)-*N*-(4-cyano-2,3,5,6-tetrafluorophenyl)-1-ethyl-2-phenylcyclobutane-1-carboxamide (S13j)

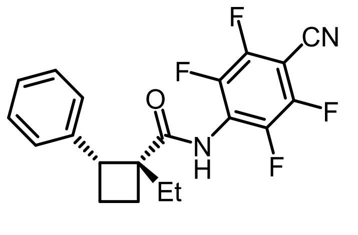

The reaction was completed following the general procedure with the ligand **L7**. The product was obtained as a white solid (30% yield, 26% *ee*).

The product was also obtained with 23% yield and 31% *ee* with the ligand **L8**.

HRMS (ESI-TOF): *m/z* Calcd for $C_{20}H_{17}F_4N_2O^+$ $[M+H]^+$ 377.1272, found 377.1279.

3.2.4 Enantioselective arylation of cyclobutanecarboxamide by ArI

General Procedure

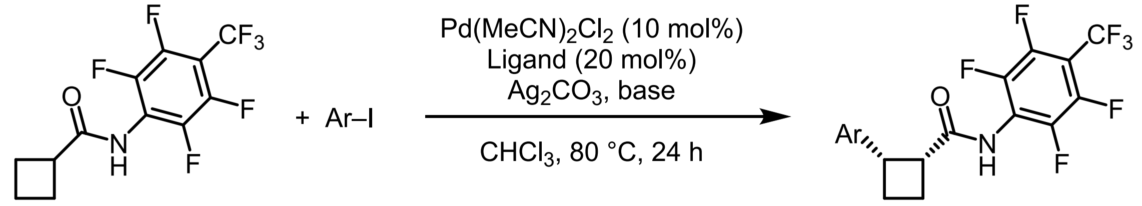

A reaction tube (10 mL) with magnetic stir bar was charged with the amide substrate (0.1 mmol), aryl iodide (0.30 mmol), $Pd(MeCN)_2Cl_2$ (0.01 mmol, 2.6 mg), ligand (0.015 mmol, 3.6 mg), Ag_2CO_3 (0.20 mmol, 55.2 mg). Chloroform (0.5 mL) was added to the tube and the tube was sealed and heated to 80 °C for 24 hours.

The crude reaction mixture was filtrated with celite and washed with EtOAc. The solvents were removed under reduced pressure and the residue was purified by preparative TLC to afford the desired product.

(1*R*,2*S*)-*N*-(2,3,5,6-tetrafluoro-4-(trifluoromethyl)phenyl)-2-(*p*-tolyl)cyclobutane-1-carboxamide (S13k)

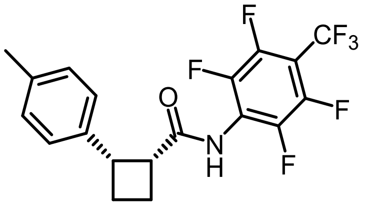

The reaction was completed following the general procedure with the ligand **L6**, and the base Li_3PO_4 (eluent: hexanes/ethyl acetate = 10/1). The product was obtained as a white solid (37% yield, 80% *ee*).

The product was also obtained with 36% yield and 81% *ee* with the ligand **L6** and the base Na_3PO_4.

^{1}H NMR (600 MHz, $CDCl_3$): δ 7.16 (d, J = 8.2 Hz, 2H), 7.14 – 7.10 (m, 2H), 6.39 (s, 1H), 4.02 (q, J = 8.8 Hz, 1H), 3.65 – 3.59 (m, 1H), 2.64 – 2.57 (m, 1H), 2.57 – 2.51 (m, 1H), 2.39 – 2.28 (m, 5H).

HRMS (ESI-TOF): *m/z* Calcd for $C_{19}H_{15}F_{7}NO^{+}$ $[M+H]^{+}$ 406.1042, found 406.1047.

4. References

[1]. P.-X. Shen, L. Hu, Q. Shao, K. Hong, J.-Q. Yu, Pd(II)-Catalyzed Enantioselective C(sp3)–H Arylation of Free Carboxylic Acids. *J. Am. Chem. Soc.* **140**, 6545–6549 (2018).

[2]. L. Hu, *et al.*, PdII-Catalyzed Enantioselective C(sp3)−H Activation /Cross-Coupling Reactions of Free Carboxylic Acids. *Angew. Chem. Int. Ed.* **58**, 2134–2138 (2019).

[3]. Q.-F. Wu, X.-B. Wang, P.-X. Shen, J.-Q. Yu, Enantioselective C–H Arylation and Vinylation of Cyclobutyl Carboxylic Amides. *ACS Catal.* **8**, 2577–2581 (2018).

[4]. K.-J. Xiao, *et al.*, Palladium(II)-Catalyzed Enantioselective C(sp3)–H Activation Using a Chiral Hydroxamic Acid Ligand. *J. Am. Chem. Soc.* **136**, 8138–8142 (2014).

[5] J. He, Q. Shao, Q. Wu, J.-Q. Yu, Pd(II)-Catalyzed Enantioselective C(sp3)–H Borylation. *J. Am. Chem. Soc.* **139**, 3344–3347 (2017).

5. NMR Spectra

(*S*)-2-acetamido-3-(3,3''-dimethyl-[1,1':3',1''-terphenyl]-2'-yl)-Nmethoxypropanamide (L1)

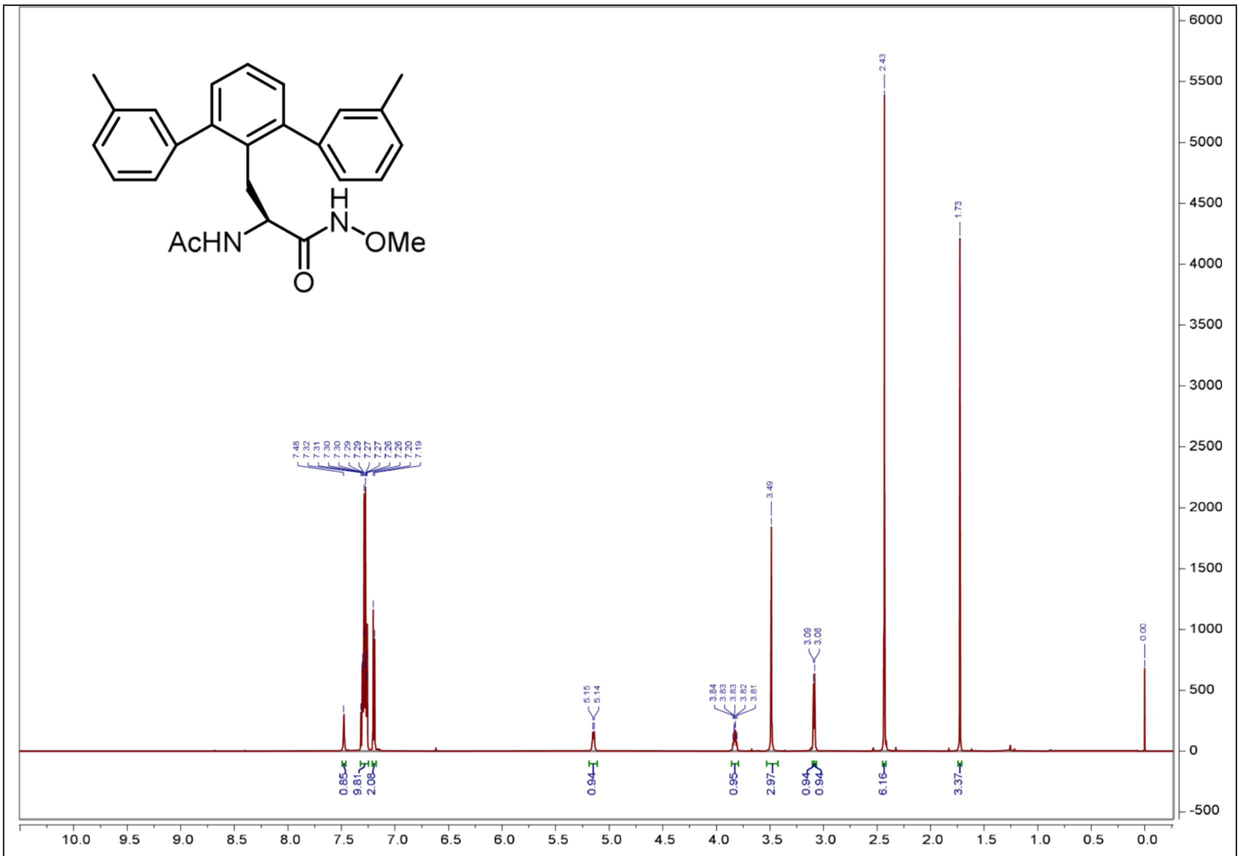
AcHN
H
N
OMe
O
7.48 7.32 7.31 7.30 7.30 7.29 7.29 7.27 7.27 7.26 7.26 7.20 7.19
5.15 5.14
3.84 3.83 3.83 3.82 3.81
3.49
3.09 3.08
2.43
1.73
0.00
0.85 9.81 2.08 0.94 0.95 2.97 0.94 0.94 6.16 3.37
10.0 9.5 9.0 8.5 8.0 7.5 7.0 6.5 6.0 5.5 5.0 4.5 4.0 3.5 3.0 2.5 2.0 1.5 1.0 0.5 0.0
6000 5500 5000 4500 4000 3500 3000 2500 2000 1500 1000 500 0 -500

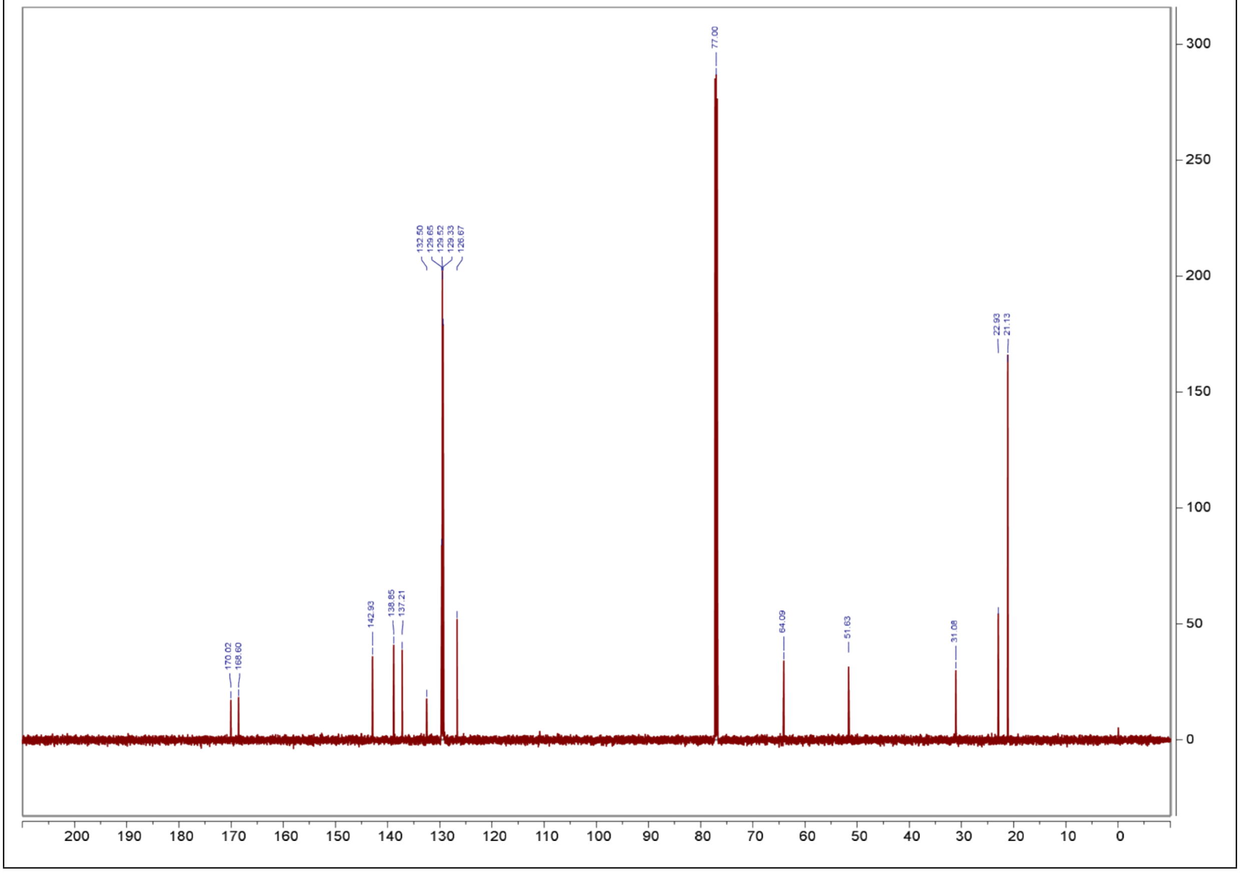
170.02 168.60
142.93
138.85 137.21
132.50 129.65 129.52 129.33 126.67
77.00
64.09
51.63
31.08
22.93 21.13
200 190 180 170 160 150 140 130 120 110 100 90 80 70 60 50 40 30 20 10 0
300 250 200 150 100 50 0

(*S*)-2-acetamido-3-(4,4''-dimethyl-[1,1':3',1''-terphenyl]-2'-yl)-Nmethoxypropanamide (L2)

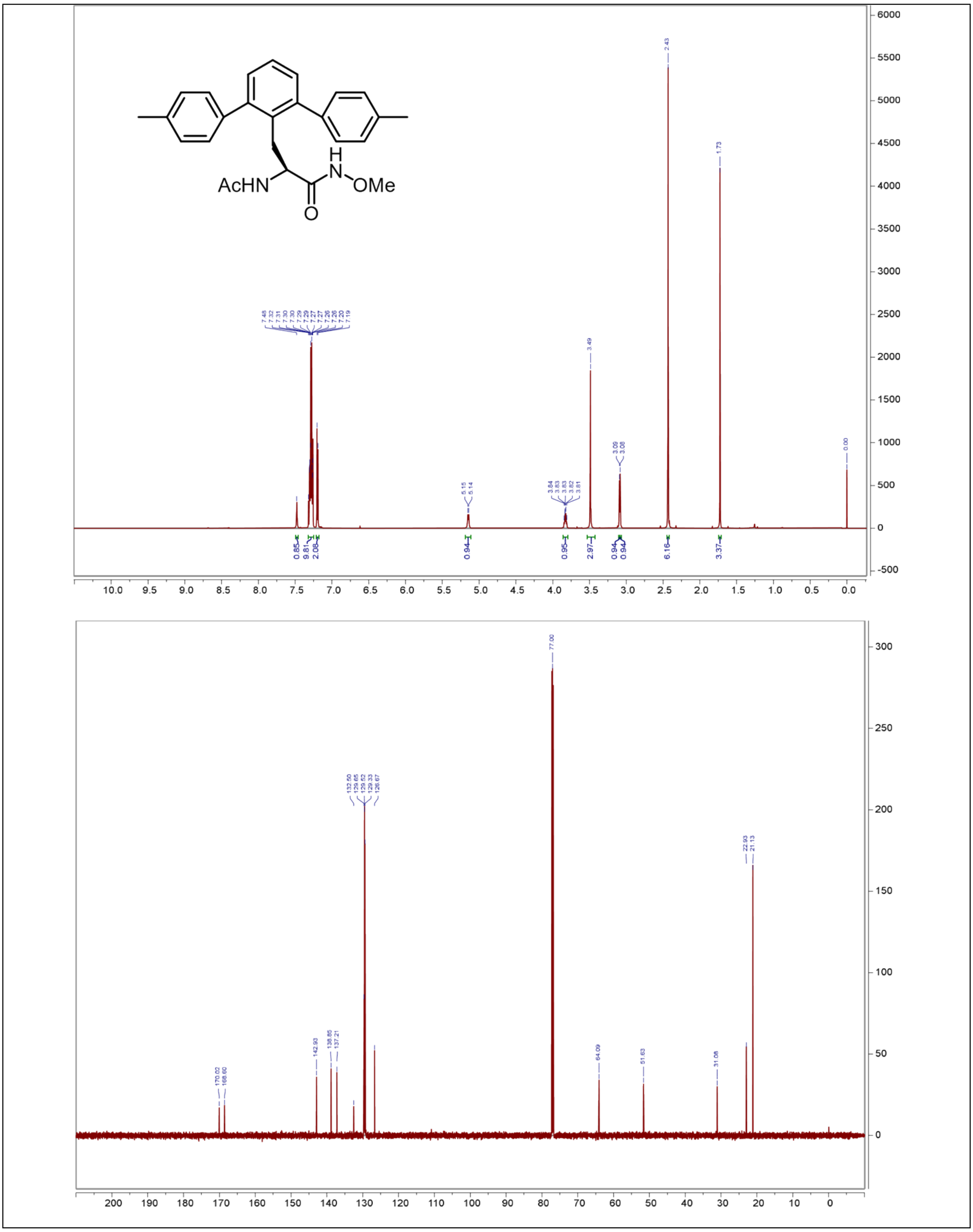

(*S*)-2-acetamido-3-(4,4''-diphenyl-[1,1':3',1''-terphenyl]-2'-yl)-Nmethoxypropanamide (L3)

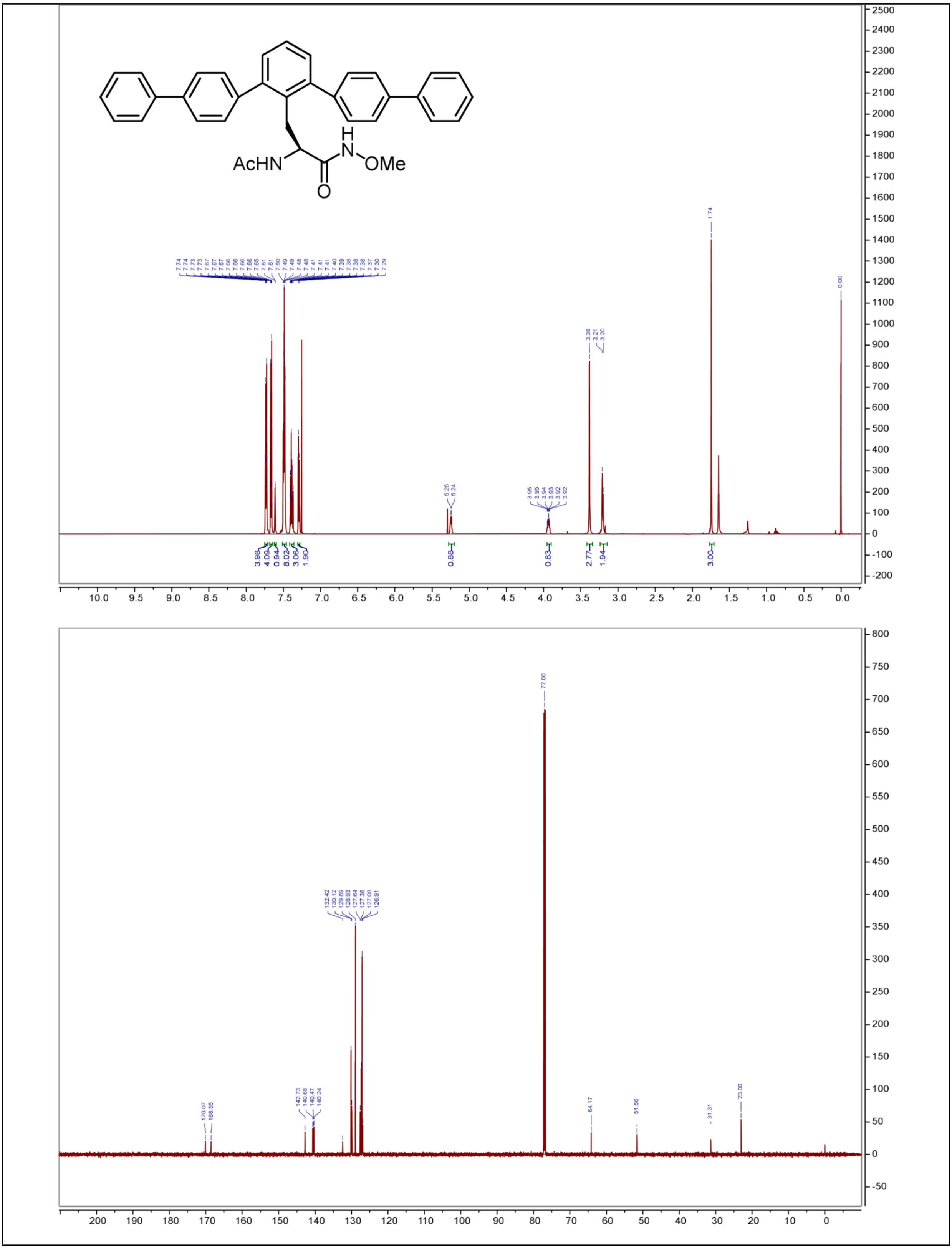

(*S*)-N-(1-(4,4''-di-tert-butyl-[1,1':3',1''-terphenyl]-2'-yl)-3-(ethylmethylamino)propan-2-yl)acetamide (L5)

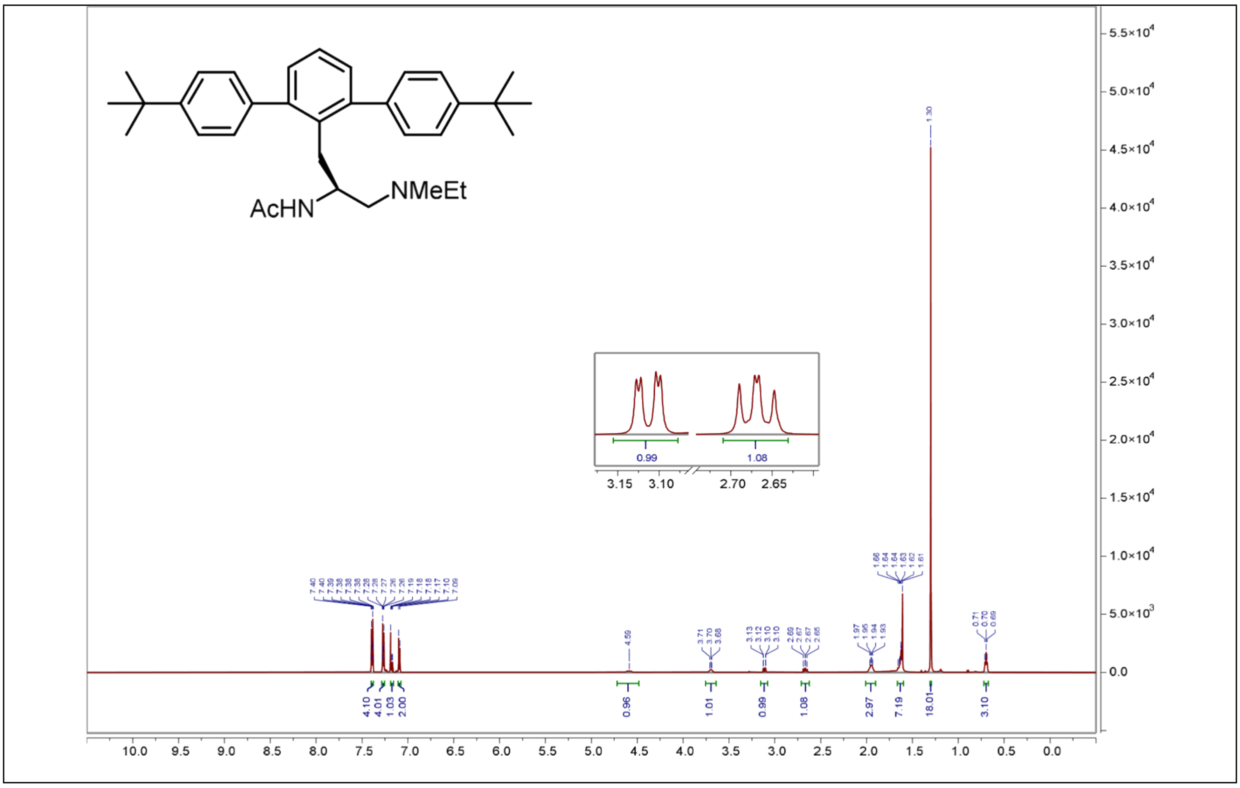

***N*-((*S*)-1-((R)-4-benzyl-4,5-dihydrooxazol-2-yl)-2,2-dimethylpropyl)acetamide (L6)**

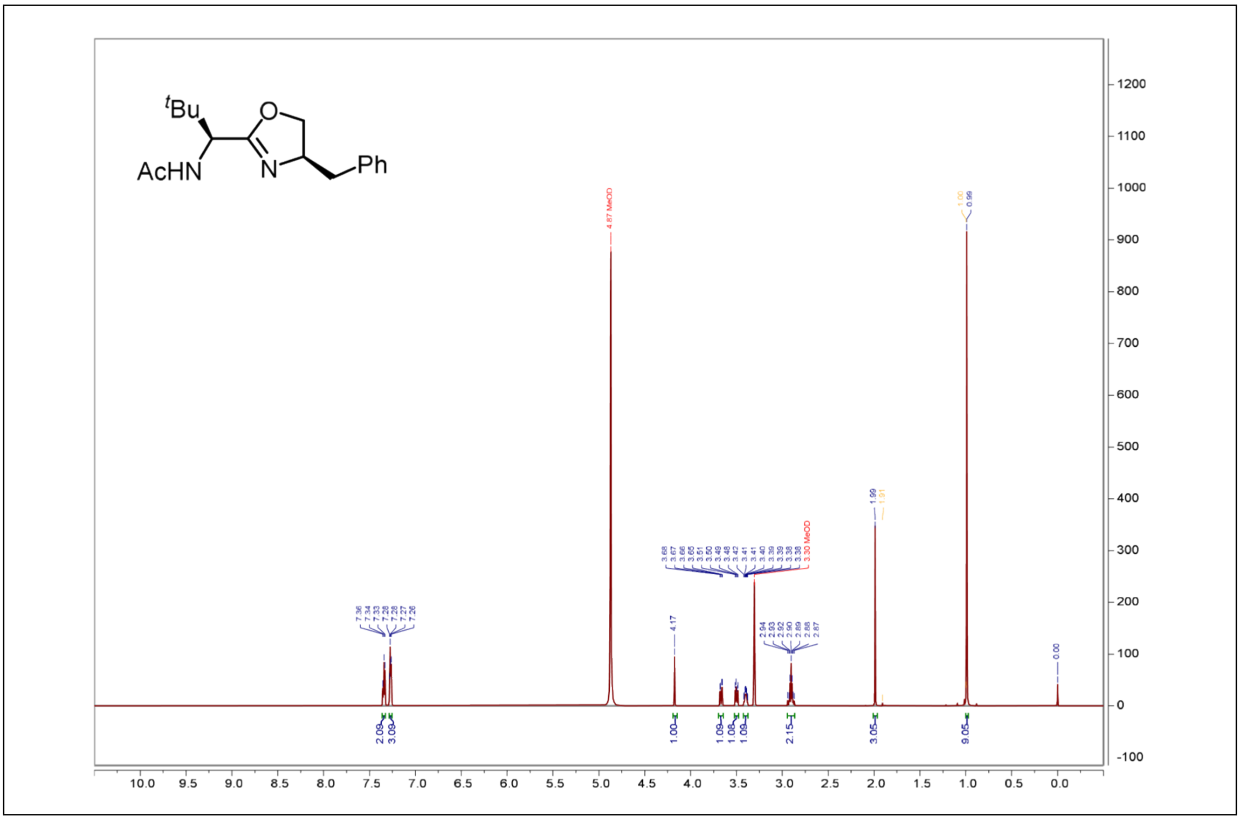

(1*R*,2*S*)-2-(3-chlorophenyl)cyclopropane-1-carboxylic acid (S13a)

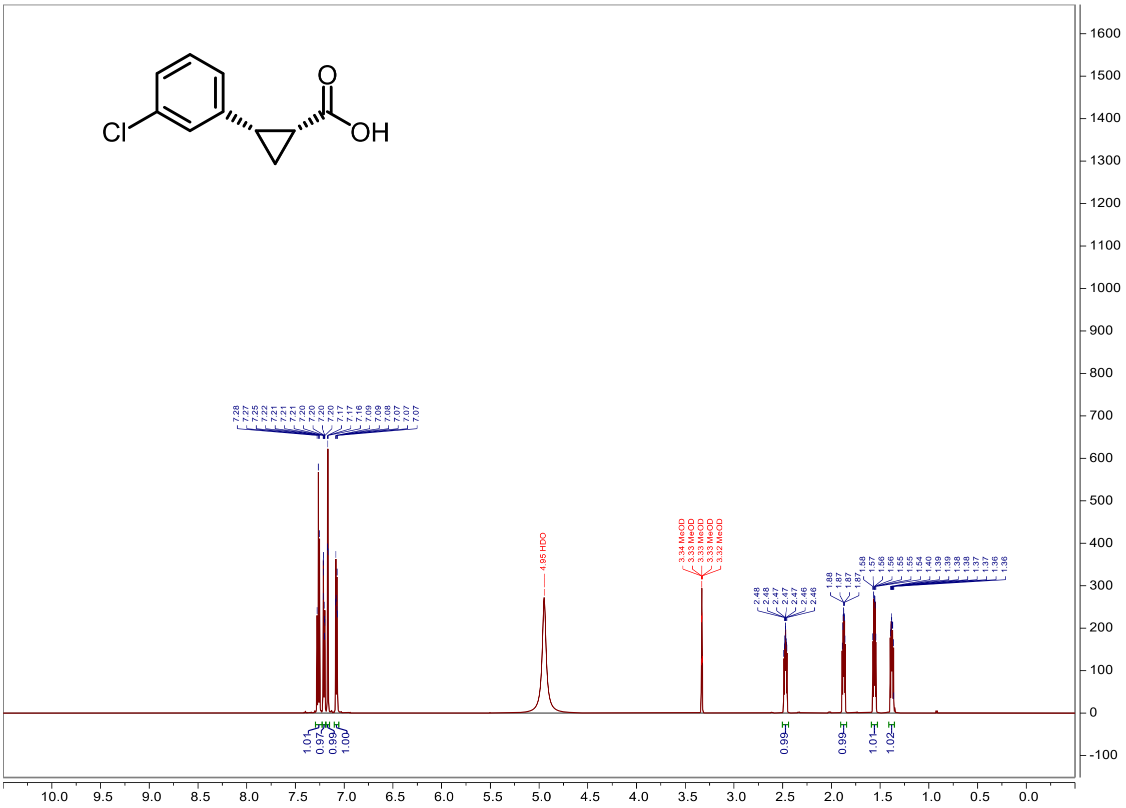

(1*R*,2*S*)-2-(3-methoxyphenyl)cyclopropane-1-carboxylic acid (S13b)

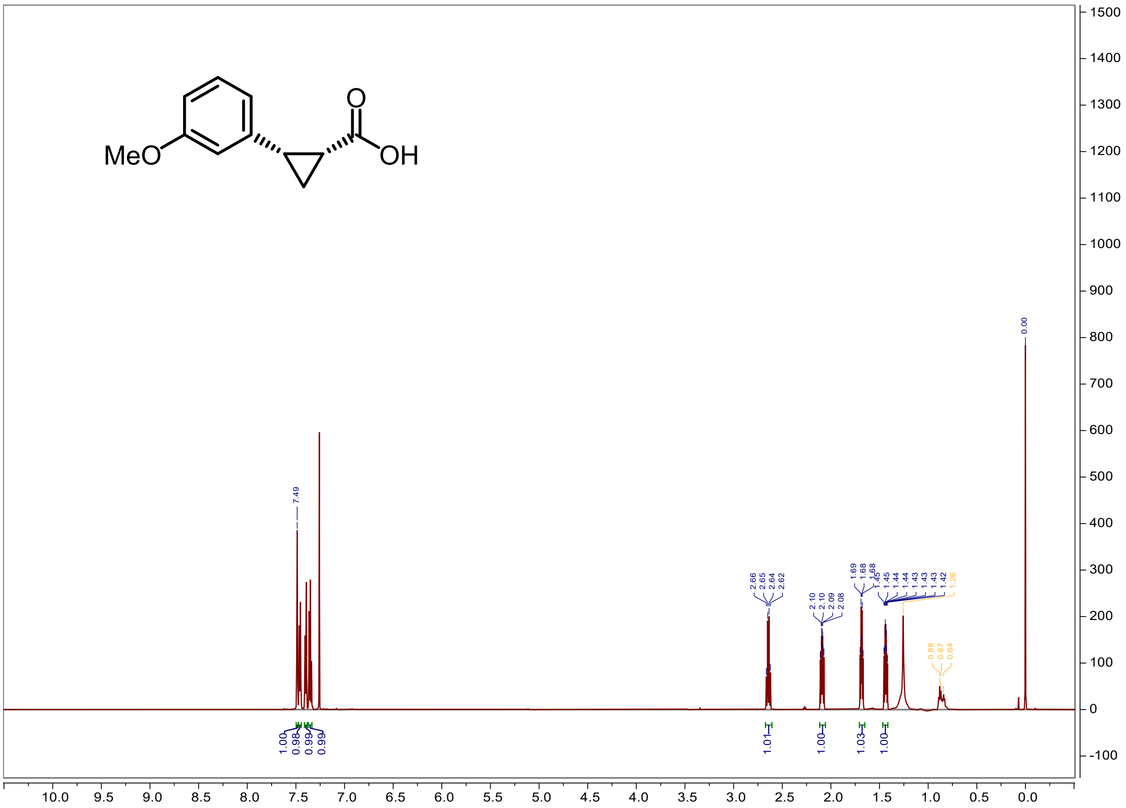

(1*R*,2*S*)-2-phenylcyclopropane-1-carboxylic acid (S13c)

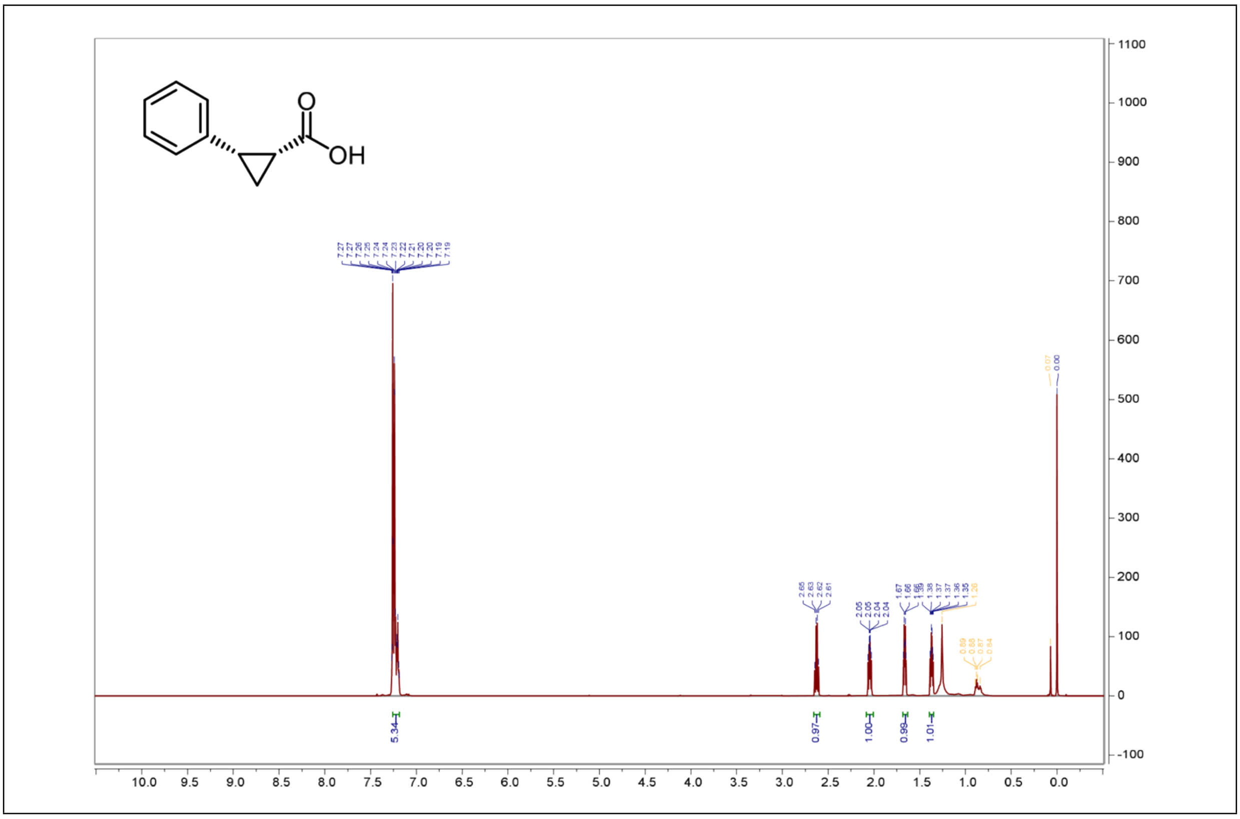

(1*R*,2*S*)-2-(4-fluorophenyl)cyclopropane-1-carboxylic acid (S13d)

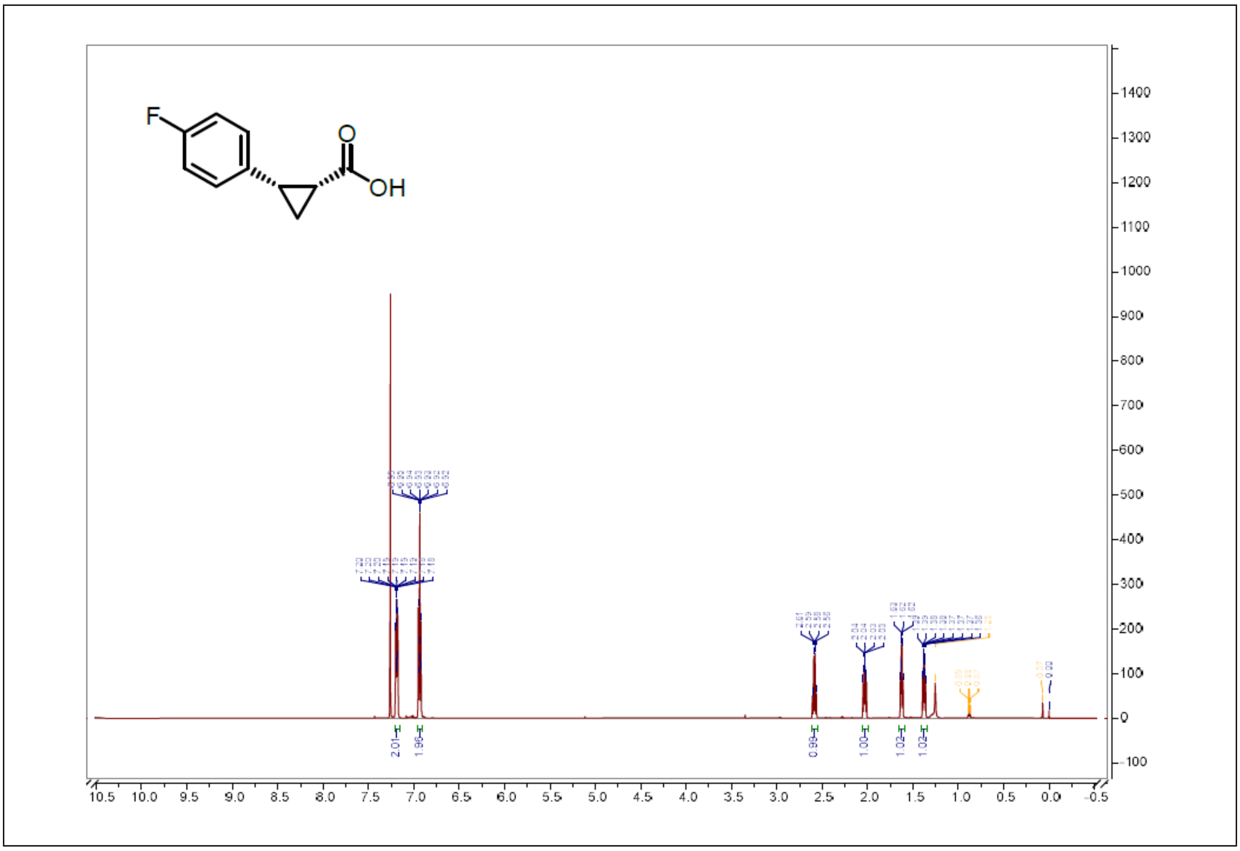

(1*R*,2*S*)-2-(3-trifluoromethylphenyl)cyclopropane-1-carboxylic acid (S13e)

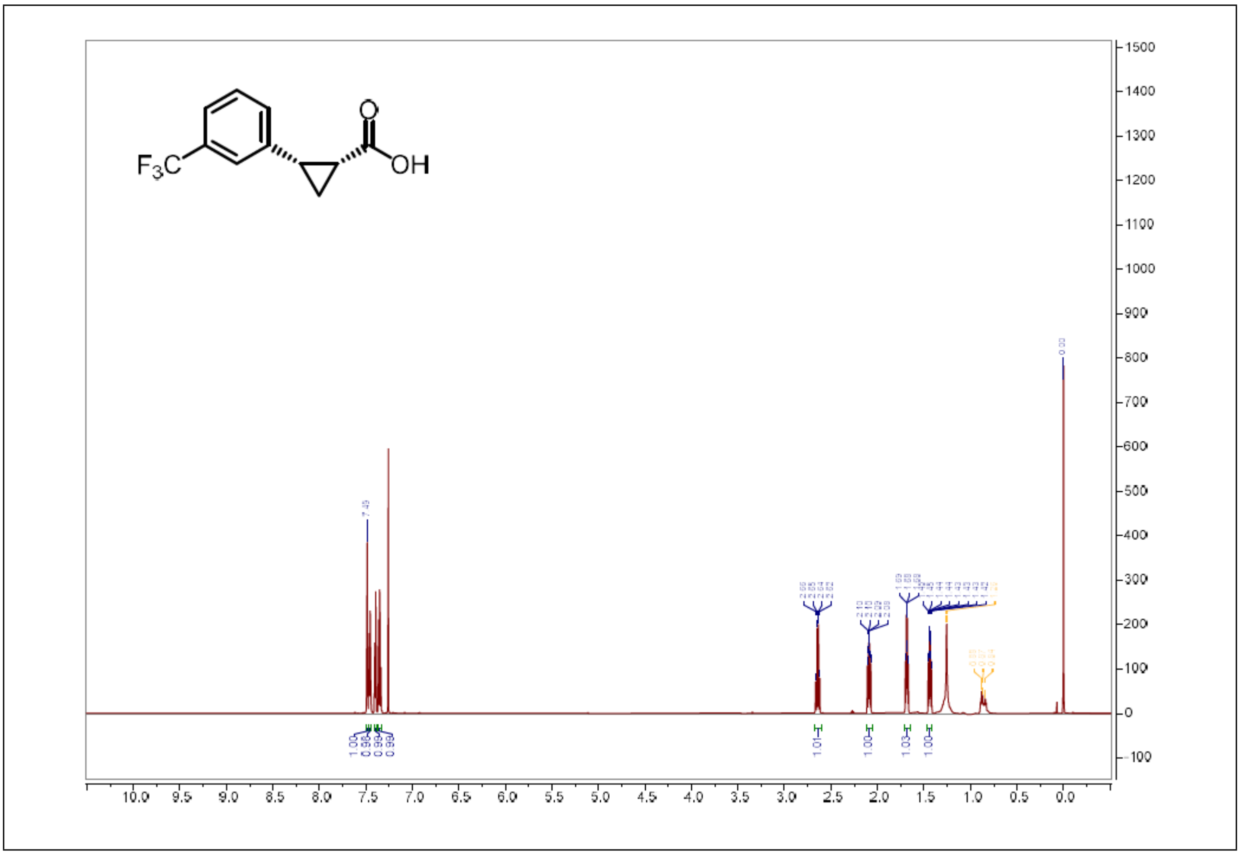

(1*R*,2*S*)-2-(3-(methoxycarbonyl)phenyl) cyclopropane-1-carboxylic acid (S13f)

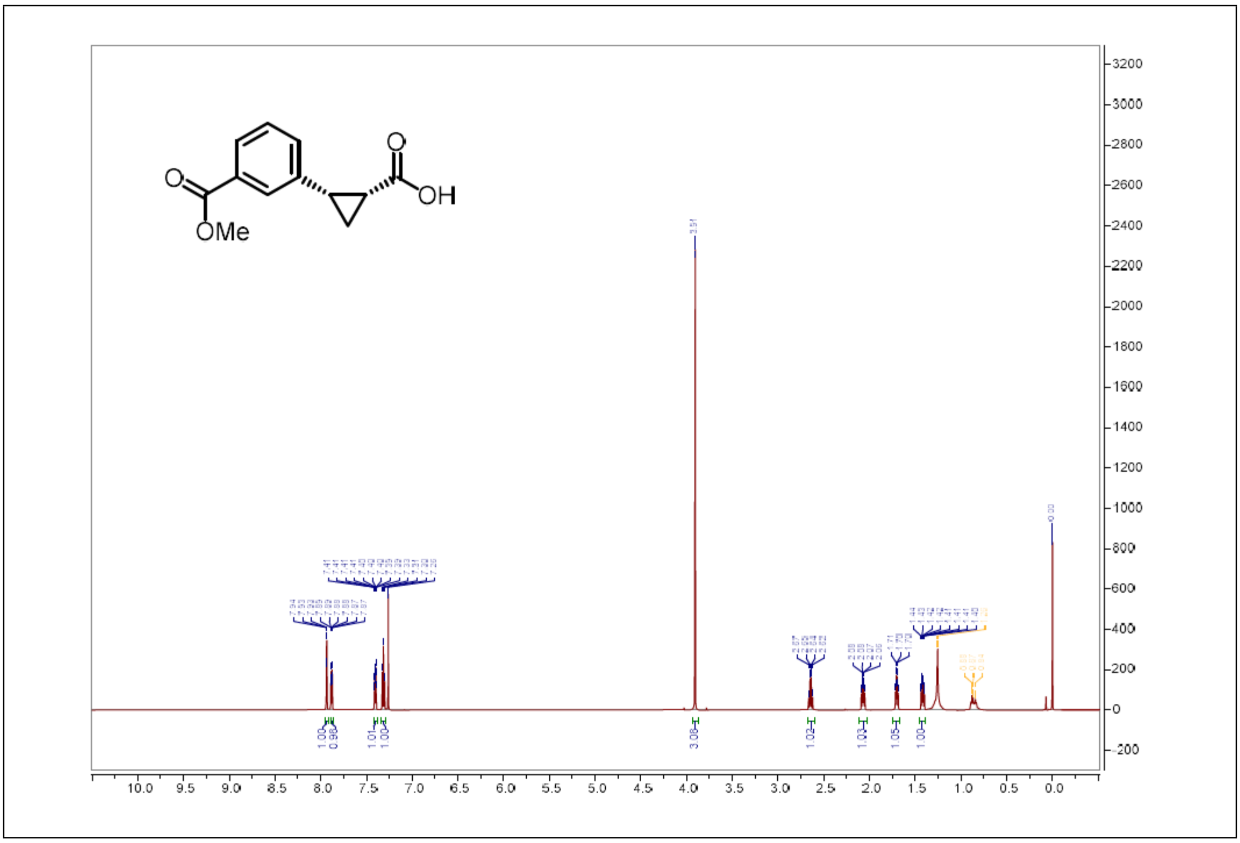

(1*R*,2*S*)-2-(4-chlorophenyl)cyclopropane-1-carboxylic acid (S13g)

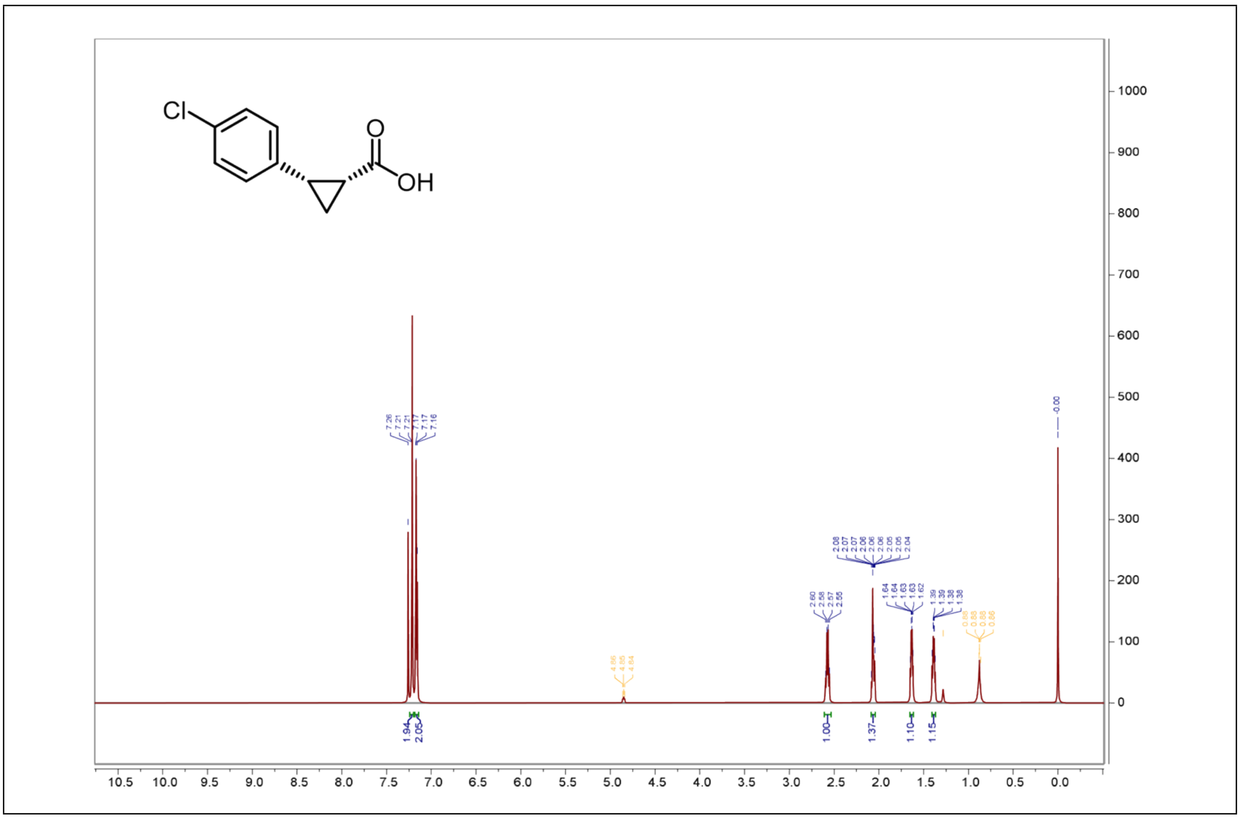

(1*R*,2*S*)-2-(4-trifluoromethoxy)cyclopropane-1-carboxylic acid (S13h)

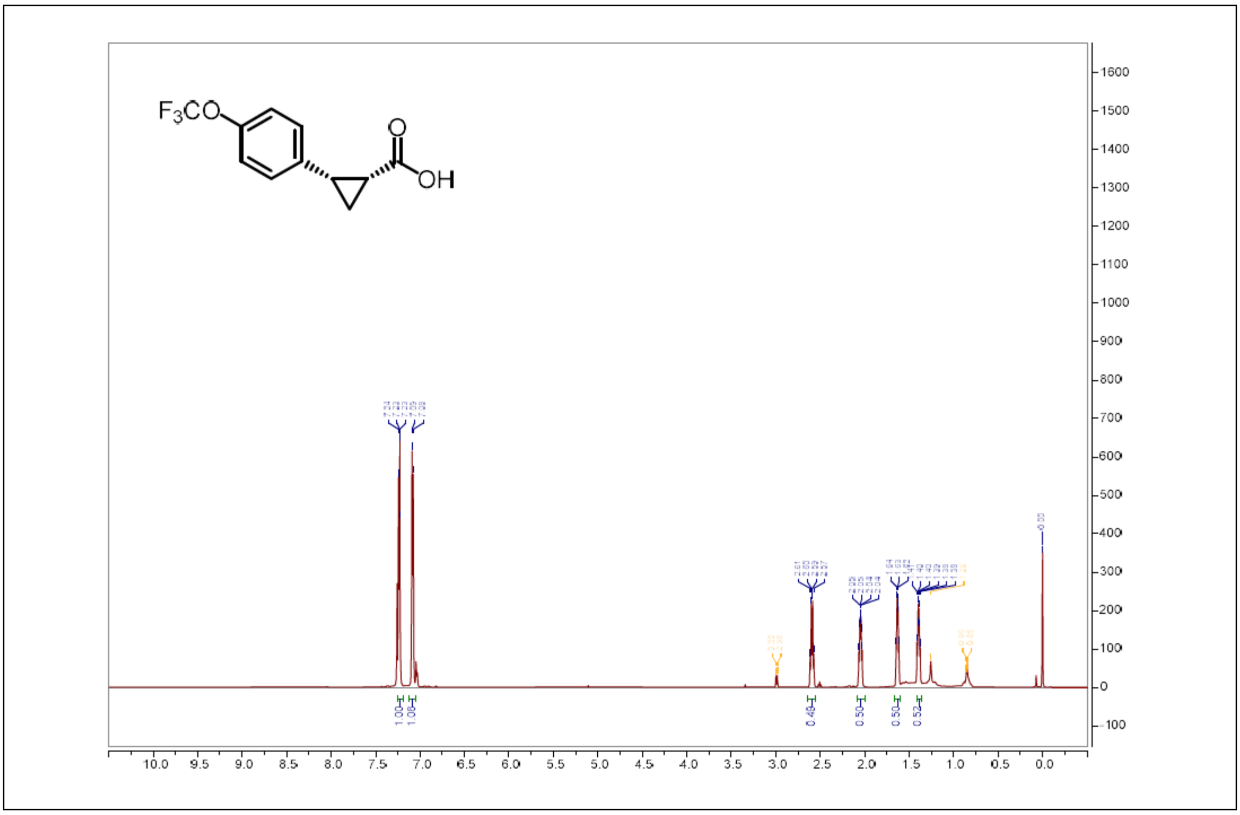

(1*R*,2*S*)-2-(4-methylphenyl)cyclopropane-1-carboxylic acid (S13i)

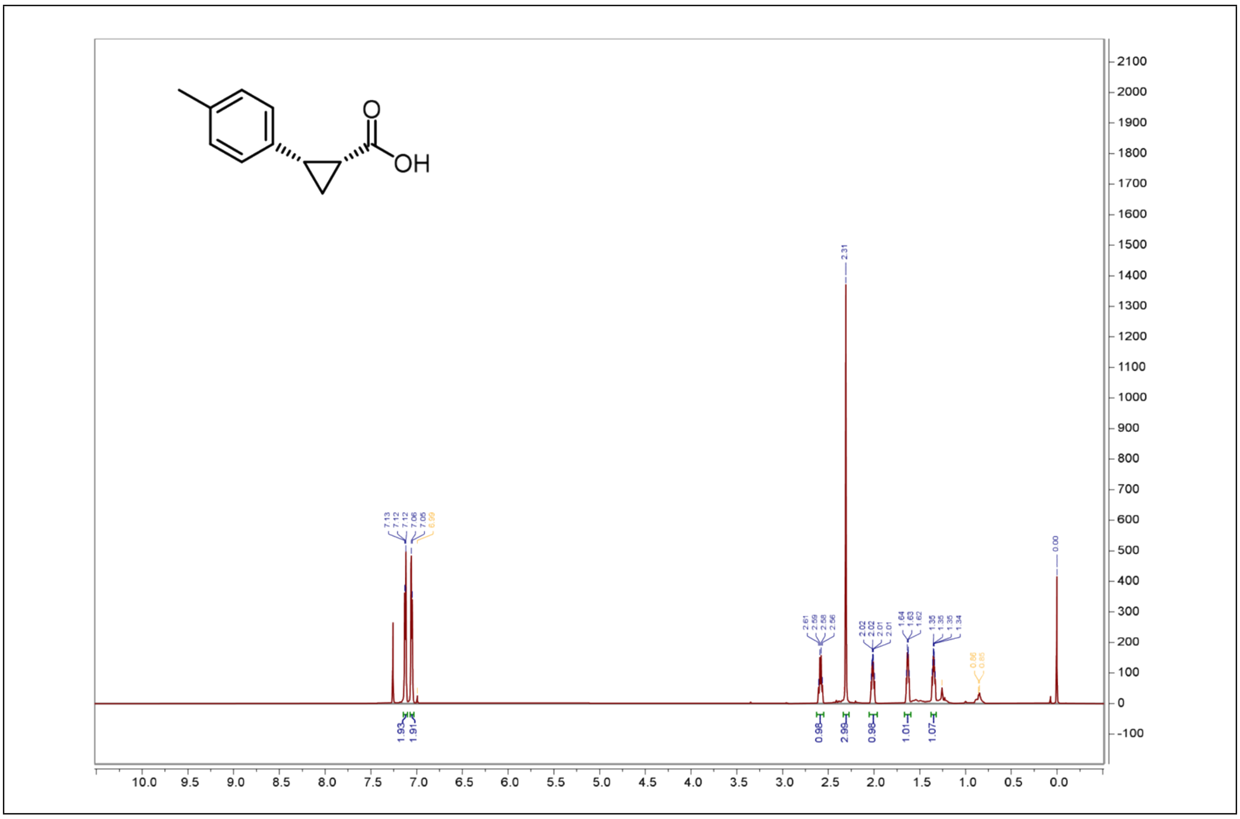

(1*R*,2*S*)-N-(2,3,5,6-tetrafluoro-4-(trifluoromethyl)phenyl)-2-(p-tolyl)cyclobutane-1 carboxamide (S13k)

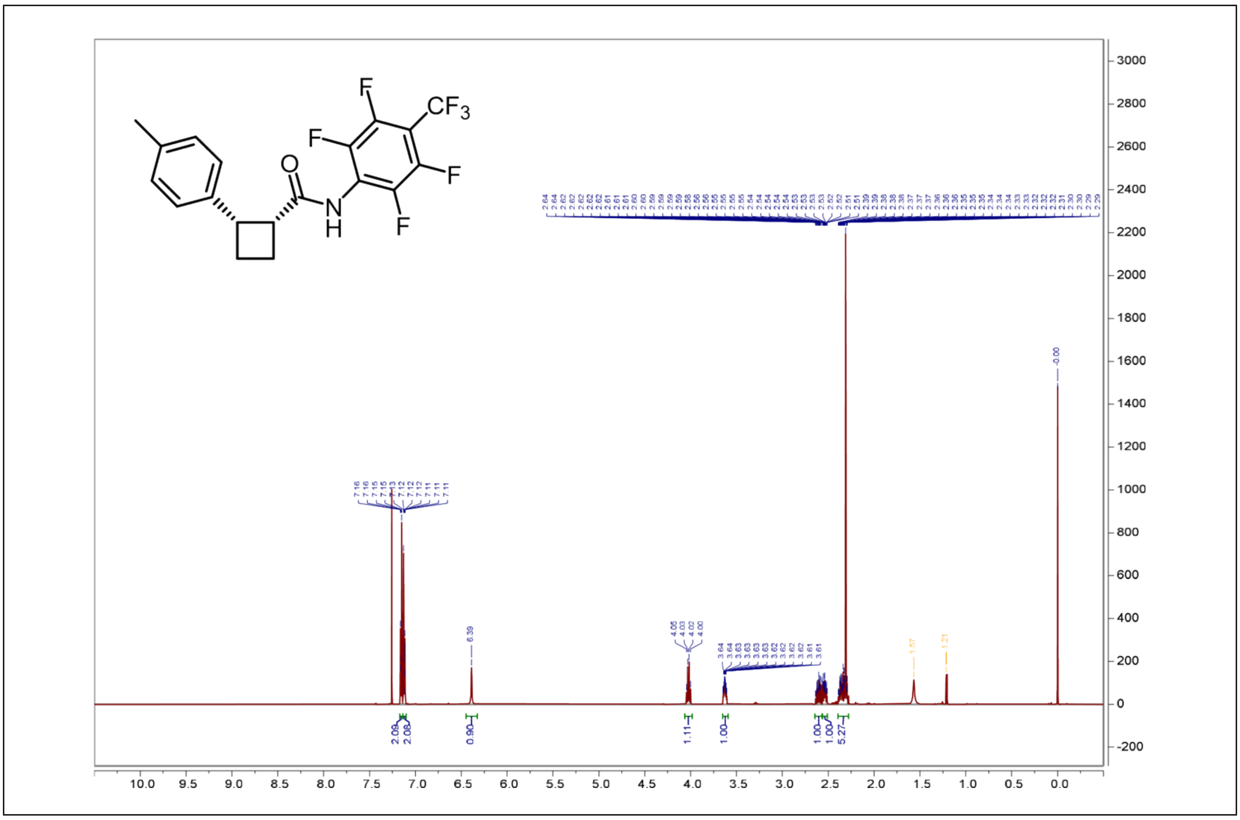

---End of file---
